# Electroacupuncture Modulates Multiple Pathways for Neuroprotection and Neurorepair in Ischemic Stroke

**DOI:** 10.1002/cns.70862

**Published:** 2026-04-07

**Authors:** Mingyue Zhao, Hangyu Shen, Xu Yan, Xiang Gao, Yuchun Liu, Shengjun Zhou, Jingxian Sun, Jie Sun, Yi Huang

**Affiliations:** ^1^ Ningbo Key Laboratory of Nervous System and Brain Function, Department of Neurosurgery The First Affiliated Hospital of Ningbo University Ningbo Zhejiang China; ^2^ Ningbo Key Laboratory of Nervous System and Brain Function, Cerebrovascular Disease Center The First Affiliated Hospital of Ningbo University Ningbo Zhejiang China; ^3^ Department of Traditional Chinese Medicine The First Affiliated Hospital of Ningbo University Ningbo Zhejiang China

**Keywords:** angiogenesis, electroacupuncture, ischemic stroke, neuroinflammation, neuroplasticity, neuroprotection

## Abstract

**Background:**

The complex pathophysiology of ischemic stroke (IS) involves initial injury followed by secondary cascades and endogenous repair. This review systematically explores how electroacupuncture (EA) precisely modulates these processes.

**Methods:**

We systematically searched and summarized all papers related to EA treatment for stroke using the China National Knowledge Infrastructure (CNKI) and PubMed databases.

**Results:**

We detail EA's key mechanisms in reducing initial and secondary damage, including the suppression of inflammatory responses (e.g., NF‐κB, inflammasomes, gut‐brain axis), mitigation of oxidative stress (Nrf2/HO‐1), attenuation of excitotoxicity (NMDAR, GLT‐1), and inhibition of diverse forms of regulated cell death (apoptosis, pyroptosis, ferroptosis). Critically, EA also actively promotes vital repair mechanisms by enhancing cerebral blood flow, protecting blood–brain barrier integrity, promoting angiogenesis (VEGF, HIF, Notch, Wnt pathways), and fostering extensive neural regeneration and plasticity (neurogenesis, neurotrophic factors, myelination, synaptic changes, network reorganization) at molecular, cellular, and network levels. These well‐documented mechanistic actions provide a strong scientific basis for the observed clinical benefits of EA in improving major poststroke sequelae, encompassing motor, cognitive, language, and emotional deficits.

**Conclusions:**

The review highlights the potential for enhanced outcomes through combined therapy approaches and identifies future research avenues leveraging advanced technologies like multi‐omics and AI for personalized precision EA. EA stands as a scientifically grounded, multi‐target therapy improving stroke recovery.

## Graphical abstract

EA exerts therapeutic effects in ischemic stroke through multiple mechanisms: it alleviates acute injury by suppressing neuroinflammation, excitotoxicity, oxidative stress, apoptosis, pyroptosis, ferroptosis, autophagy dysregulation, and blood–brain barrier disruption, while enhancing cerebral blood flow, angiogenesis, and neurogenesis to support neural repair and remodeling. Collectively, these integrated actions promote poststroke functional recovery, leading to significant improvements in cognition, learning and memory, language, swallowing, motor function, and poststroke depression.

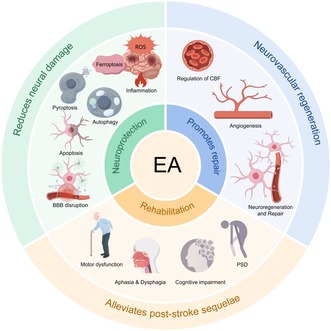

**Abbreviations**
5‐HT1A: 5‐hydroxytryptamine 1A
AAV: adeno‐associated virus
Abca1: ATP‐binding cassette transporter A1
ACSL4: acyl‐CoA synthetase long‐chain family member 4
ADAMTS1: A disintegrin and metalloproteinase with thrombospondin motifs 1
ADL: activities of daily living
AIS: acute ischemic stroke
AKT: protein kinase B
ALP: alkaline phosphatase
Ang1/2: angiopoietin‐1/2
AQP4/9: aquaporin‐4/9
ASC: apoptosis‐associated speck‐like protein containing a CARD
ATP: adenosine triphosphate
Aβ: beta‐amyloid
Bad: Bcl‐2‐associated death promoter
Bax: Bcl‐2‐associated X protein
BBB: blood–brain barrier
Bcl‐2: B‐cell lymphoma 2
BDNF: brain‐derived neurotrophic factor
Beclin1: beclin‐1 (autophagy‐related protein)
bFGF: basic fibroblast growth factor
BMSC: bone marrow stromal cell
CB1R: cannabinoid receptor 1
CBF: cerebral blood flow
CCAS3: cleaved caspase‐3
CD3^+^TCRγδ^+^CFSE^+^
: cluster of differentiation 3‐positive T‐cell receptor γδ‐positive carboxyfluorescein succinimidyl ester‐positive
CD81: CD81 molecule (exosomal marker)
CIRI: cerebral ischemia–reperfusion injury
COX: cyclooxygenase
CPA: cricopharyngeal achalasia
CREB: cAMP response element‐binding protein
CSF: cerebrospinal fluid
CST: corticospinal tract
CT: computed tomography
CuZnSOD: copper‐zinc superoxide dismutase
CX3CL1: C‐X3‐C motif chemokine ligand 1
CXCR4: C‐X‐C chemokine receptor type 4
CYLD: cylindromatosis
CYP24A: cytochrome P450 family 24 subfamily A
CYP27A1/B1: cytochrome P450 family 27 subfamily A1/B1
DMN: default mode network
Drp1: dynamin‐related protein 1
EA: electroacupuncture
EAA: excitatory amino acid
EC: endothelial cell
Efnb2: ephrin‐B2
eNOS: endothelial nitric oxide synthase
EPC: endothelial progenitor cell
EPO: erythropoietin
ERK: extracellular signal‐regulated kinase
ES: electrical stimulation
FCI: focal cerebral ischemia
FGF9: fibroblast growth factor 9
Fis1: mitochondrial fission 1 protein
FLK1: fetal liver kinase 1 (VEGFR2)
fMRI: functional magnetic resonance imaging
FOXO1: forkhead box O1
FOXP3+ Treg: forkhead box P3‐positive regulatory T cells
FTH1: ferritin heavy chain 1
GABA: gamma‐aminobutyric acid
GAP‐43: growth‐associated protein 43
GBD: Global Burden of Disease Report
GDNF: glial cell‐derived neurotrophic factor
GFAP: glial fibrillary acidic protein
GLT‐1: glutamate transporter‐1
Glu: glutamate
GluR2: glutamate receptor 2
GPX4: glutathione peroxidase 4
GR: glucocorticoid receptor
GSDMD: gasdermin D
GSH: glutathione
GSH‐Px: glutathione peroxidase
GSK‐3β: glycogen synthase kinase 3 beta
H3K27ace: histone H3 lysine 27 acetylation
H3K9ace: histone H3 lysine 9 acetylation
HA: head acupuncture
HIF‐1α: hypoxia‐inducible factor‐1 alpha
HMGB1: high mobility group box 1
HO‐1: heme oxygenase 1
HSP: hemiparetic shoulder pain
I/R: ischemia/reperfusion
ICA: independent component analysis
IL‐10: interleukin‐10
IL‐17: interleukin‐17
IL‐17A+ Th17: Interleukin‐17A‐positive T helper 17 cells
IL‐18: interleukin‐18
IL‐1β: interleukin‐1 beta
IL‐33: interleukin‐33
IL‐6: interleukin‐6
IL‐8: interleukin‐8
IPA: indole‐3‐propionic acid
iPSC‐EV: induced pluripotent stem cell‐derived extracellular vesicles
IS: ischemic stroke
JAK2: janus kinase 2
JNK: c‐Jun N‐terminal kinase
K81: lysine 81
Keap1: Kelch‐like ECH‐associated protein 1
Ki67: Ki‐67 protein (cell proliferation marker)
Kla: lactylation of lysine
LAMP1: lysosomal‐associated membrane protein 1
LC3‐II: microtubule‐associated protein 1 light chain 3‐II
LC3β: microtubule‐associated protein 1 light chain 3 beta
LIMK1: LIM domain kinase 1
LRRC4: leucine‐rich repeat‐containing 4
MA: manual acupuncture
MAP2: microtubule‐associated protein 2
MAPK: mitogen‐activated protein kinase
MCAO: middle cerebral artery occlusion
MCP‐1: monocyte chemoattractant protein‐1
MDA: malondialdehyde
MGBA: microbiome‐gut‐brain axis
miR‐142‐5p: microRNA‐142‐5p
miR‐146b: microRNA‐146b
miR‐210: microRNA‐210
miR‐34c‐5p: microRNA‐34c‐5p
miR‐381: microRNA‐381
MMP: mitochondrial membrane potential
MMP‐2/9: matrix metalloproteinase‐2/9
MnSOD: manganese superoxide dismutase
mNSS: modified neurological severity score
mTOR: mechanistic target of rapamycin
mTORC1: mammalian target of rapamycin complex 1
NBM: Meynert basal nucleus
NEK7: NIMA‐related kinase 7
NeuN: neuronal nuclear antigen
NF‐κB: nuclear factor kappa B
NG2: neuron‐glial antigen 2
NGF: nerve growth factor
NLRP3: NOD‐like receptor family pyrin domain containing 3
NMDA: N‐methyl‐D‐aspartate
NMDAR: N‐methyl‐D‐aspartate receptor
NMES: neuromuscular electrical stimulation
NO: nitric oxide
Nogo‐A: neurite outgrowth inhibitor A
Nrf2: nuclear factor erythroid 2‐related factor 2
NSCs: neural stem cells
NSE‐IR: neuron‐specific enolase‐immunoreactive
OGD/R: oxygen–glucose deprivation/reoxygenation
OM: oxygen therapy
OPA1: Optic atrophy 1
OTULIN: OTU deubiquitinase with linear linkage specificity
p38: p38 mitogen‐activated protein kinase
p53: tumor protein p53
Pak4: p21‐activated kinase 4
p‐Akt: phosphorylated protein kinase B
p‐Bad: phosphorylated Bcl‐2‐associated death promoter
PCD: programmed cell death
PDGF‐b: platelet‐derived growth factor subunit B
PET‐CT: positron emission tomography‐computed tomography
PGC‐1α: peroxisome proliferator‐activated receptor gamma coactivator 1‐alpha
PI3K: phosphoinositide 3‐kinase
PINK1: PTEN‐induced kinase 1
PIVD: postinfarction vascular dementia
PKA: protein kinase A
PP2A: protein phosphatase 2A
PPARγ: peroxisome proliferator‐activated receptor gamma
PSCI: poststroke cognitive impairment
PSD: poststroke depression
PTEN: phosphatase and tensin homolog
PTX3: pentraxin 3
PV: parvalbumin
RAGE: receptor for advanced glycation end products
RhoA: Ras homolog family member A
RORγt: retinoic acid‐related orphan receptor gamma t
ROS: reactive oxygen species
RSNs: resting‐state networks
RT: rehabilitation training
SCF: stem cell factor
SCFA: short‐chain fatty acid
SCN: scalp ring acupuncture
SDF‐1: stromal cell‐derived factor‐1
SIRT1/5: sirtuin 1/5
SLC7A11: solute carrier family 7 member 11
SLR: Shur language rehabilitation
SMES: specific pattern electroacupuncture stimulation
SMN: sensorimotor network
SN: salience network
SOD: superoxide dismutase
Src: proto‐oncogene tyrosine‐protein kinase Src
SSH1: slingshot homolog 1
ST2: suppression of tumorigenicity 2
STAT1/3/6: signal transducer and activator of transcription 1/3/6
SVZ: subventricular zone
SYP: synaptophysin
TE: tenuigenin
TERT: telomerase reverse transcriptase
Tf/TfR1: transferrin/transferrin receptor 1
TGF‐β: transforming growth factor‐beta
Th1/Th2: T helper 1/T helper 2
Th17: T helper 17
TIM17A: translocase of inner mitochondrial membrane 17A
TIMP1/2: tissue inhibitor of metalloproteinases 1/2
TJ: tight junction
TLR4: toll‐like receptor 4
TNF‐α: tumor necrosis factor‐alpha
TOM40: translocase of outer mitochondrial membrane 40
Treg: regulatory T cell
TrkA: tropomyosin receptor kinase A
TrkB: tropomyosin receptor kinase B
TRPV1/4: transient receptor potential vanilloid 1/4
TSG101: tumor susceptibility gene 101
TXNIP: thioredoxin‐interacting protein
ULMD: upper limb motor dysfunction
VaD: vascular dementia
VEGFA: vascular endothelial growth factor A
WCF: wet compress formula
Wnt/β‐catenin: Wnt/beta‐catenin signaling pathway
XNKQ: Xingnao Kaiqiao acupuncture technique
ZO‐1: zonula occludens‐1
γ‐GCS: gamma‐glutamylcysteine synthetase
γ‐GCSh: gamma‐glutamylcysteine synthetase heavy subunit
γ‐GCSl: gamma‐glutamylcysteine synthetase light subunit
γδ T: gamma delta T cells

## 1 Introduction

According to the Global Burden of Disease (GBD) 2021 report, stroke stands as the second leading cause of death and the third leading cause of disability and death globally among noncommunicable diseases [1]. Ischemic stroke (IS) constitutes the predominant subtype, accounting for 60%–70% of all stroke incidents worldwide [2]. IS is characterized as a clinical syndrome resulting from cerebral vascular abnormalities that impair cerebral blood flow (CBF). This rapid reduction in blood supply leads to localized ischemia, subsequent hypoxic necrosis of brain tissue, and consequently, functional deficits [3]. A key feature of acute cerebral ischemia is the existence of a central necrotic core. This core is enveloped by a potentially salvageable region known as the ischemic penumbra. Prompt restoration of blood flow within a critical time window, or the implementation of other effective therapeutic approaches, holds the potential to mitigate brain tissue damage, foster neuronal survival, and promote functional recovery. However, the process of reperfusion to the ischemic area can paradoxically intensify brain injury, a phenomenon widely recognized as cerebral ischemia–reperfusion injury (CIRI) [4]. Current principal therapeutic strategies for IS prioritize the rapid restoration of CBF within a defined treatment window. These approaches include pharmacological thrombolysis, mechanical thrombectomy, remote ischemic conditioning [5], and other interventional techniques aimed at recanalizing occluded vessels and reestablishing adequate blood supply to the ischemic region [2, 6]. Notwithstanding these advancements in vascular recanalization treatments, alongside neuroprotectants [7] and various symptomatic management strategies, there persists a critical and unmet need to investigate safe, effective, and holistic therapeutic strategies that can further promote neurological recovery and enhance long‐term prognosis.

**FIGURE 1:**
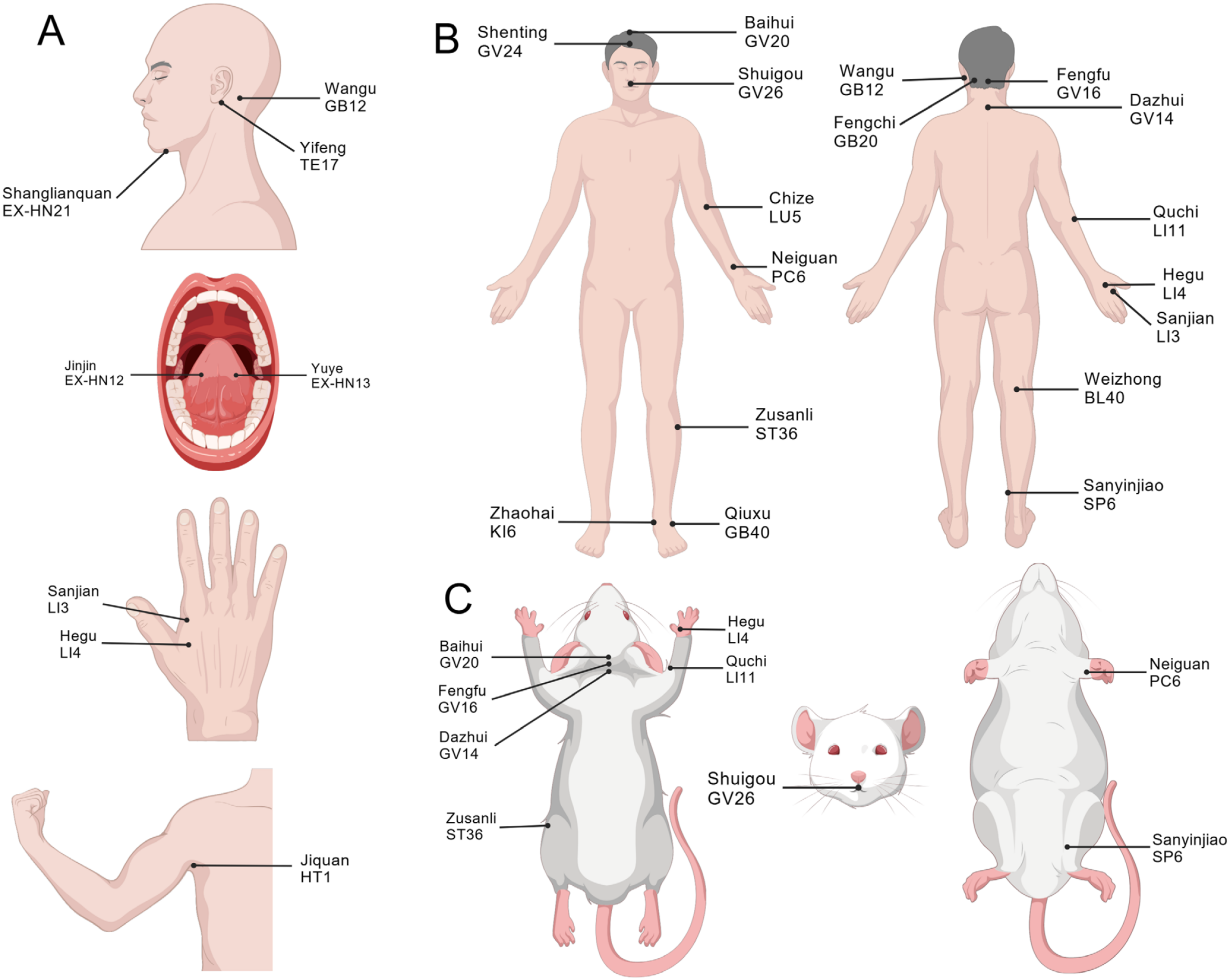
Common acupuncture points for EA treatment of IS. (A) Some acupuncture points on the human body. Wangu (GB12): In the head, in the depression posterior and inferior to the mastoid process. Yifeng (TE17): On the neck, in the depression located just in front of the lower end of the mastoid process, behind the earlobe. Shanglianquan (EX‐HN21): In the submandibular region, on the midline of the neck, 33.3 mm directly above the thyroid cartilage. Jinjin (EX‐HN12): On the lower part of the tongue, at the left side of the venous vessel beneath the frenulum of the tongue. Yuye (EX‐HN13): On the lower part of the tongue, at the right side of the venous vessel beneath the frenulum of the tongue. Sanjian (LI3): On the dorsum of the hand, at the radial side of the second metacarpal, in the depression posterior to the head of the metacarpal bone. Hegu (LI4): On the dorsum of the hand, at the midpoint of the radial side of the second metacarpal bone. Jiquan (HT1): In the axillary region, at the center of the axilla, where the pulsation of the axillary artery is felt. (B) Distribution of acupuncture points on the human body. Baihui (GV20): At the head, 166.5 mm directly above the midpoint of the forehead hairline. Shenting (DU24): Located on the head, 16.5 mm directly above the midpoint of the anterior. Shuigou (GV26): On the face, at the intersection of the upper one‐third and middle one‐third of the philtrum. Chize (LU5): In the elbow region, on the transverse crease of the elbow, in the depression at the radial side of the biceps brachii tendon. Neiguan (PC6): On the palm side of the forearm, 66.6 mm proximal to the distal transverse crease of the wrist, located between the tendon of the palmaris longus and the tendon of the flexor carpi radialis. Zusanli (ST36): On the lateral side of the lower leg, 100 mm below the knee (below the popliteal crease). Qiuxu (GB40): On the dorsum of the foot, just below the lateral malleolus, on the outer side of the tendon of the extensor hallucis longus, in the depression between the ankle joint and the heel. Zhaohai (KI6): On the medial side of the foot, in the depression just below the tip of the medial malleolus. Fengchi (GB20): In the posterior neck region, in the depression below the occipital bone, between the attachment of the sternocleidomastoid muscle and the upper end of the trapezius muscle. Fengfu (DU16): In the posterior neck region, 33.3 mm directly above the posterior hairline, below the external occipital protuberance, in the depression between the two sides of the trapezius muscles. Dazhui (GV14): In the spinal region, in the depression below the spinous process of the seventh cervical vertebra, along the posterior midline. Quchi (LI11): In the elbow region, at the midpoint of the line connecting the cubital crease (Chize) and the lateral epicondyle of the humerus. Weizhong (BL40): At the back of the knee, at the midpoint of the popliteal crease. Sanyinjiao (SP6): On the inner side of the lower leg, 100 mm above the medial malleolus, at the posterior edge of the tibia. (C) Distribution of acupuncture points on rats. Shuigou (GV26): At the midline, 1 mm below the tip of the nose. Baihui (GV20): The midline of the parietal bone. Fengfu (GV16): The depression located posterior to the occipital ridge, at the atlanto‐occipital joint. Dazhui (GV14): At the midpoint of the back, between the 7th cervical vertebra and the 1st thoracic vertebra. Hegu (LI4): Between the first and second metacarpal bones of the forelimb. Quchi (LI11): In the depression located on the anterolateral aspect of the proximal radius, distal to the radial head. Zusanli (ST36): On the posterolateral aspect of the knee joint, approximately 3 mm below the head of the fibula. Neiguan (PC6): On the medial aspect of the forelimb, approximately 3 mm from the wrist joint, within the radioulnar interspace. Sanyinjiao (SP6): On the hindlimb, 10 mm directly above the medial malleolus (created with BioGDP.com [12]).

Amidst the search for comprehensive therapeutic strategies, acupuncture has emerged as a prominent modality. As a traditional therapeutic method with a history spanning thousands of years in China [8, 9], acupuncture is widely applied in the management of stroke [10]. This technique involves the insertion of fine metallic needles into specific acupuncture points located on the body's surface to stimulate physiological responses. While needles are categorized into various types, the filiform needle is predominantly employed in the treatment of IS [11]. Conceptually, acupuncture for IS operates by stimulating these designated points along the body's meridian pathways (Figure 1). Stimulation through needling is believed to influence the flow of Qi and blood along these meridians, aiming to clear stagnation and restore physiological balance [8]. This mechanism is posited to address underlying pathological factors, leading to the alleviation of symptoms and contributing to disease treatment.

Among the various acupuncture approaches applied in stroke rehabilitation, the Xingnao Kaiqiao (XNKQ) technique [13] stands out as a widely recognized and extensively studied method in China. Developed in 1972 by Professor Shi Xuemin, an academician of the Chinese Academy of Engineering, this technique is grounded in extensive clinical experience and research [14]. The XNKQ technique has demonstrated notable efficacy and safety in the comprehensive management of IS, evidenced by its ability to significantly reduce disability rates, improve overall clinical outcomes, and enhance patients' functional independence in activities of daily living [15, 16, 17, 18]. Treatment with the XNKQ technique is individualized based on the patient's specific clinical presentation. It primarily emphasizes the stimulation of core points such as Neiguan (PC6), Shuigou (DU26) [19], and Sanyinjiao (SP6) [20] traditionally aimed at invigorating brain function and nourishing related organ systems. Additionally, auxiliary points, including Jiquan (HT1), Chize (LU5), and Weizhong (BL40), are utilized to further promote meridian flow and regulate the circulation of Qi and blood. Furthermore, tailored point combinations are employed to target specific poststroke symptoms. Tailored point combinations are employed to target specific poststroke symptoms. For example, Fengchi (GB20) [21], Wangu (GB12), and Yifeng (TE17) [22] are used for dysphagia, while Jinyin (EX‐HN12), Yuye (EX‐HN13), and Shanglianquan (EX‐HN21) are selected for bloodletting techniques aimed at speech disorders. Other combinations include Hegu (LI4) and Sanjian (LI3) to enhance finger grip strength, Qiuxu (GB40) and Zhaohai (KI6) for the correction of foot inversion, and Jianyu (LI15), Jianliao (TE14), and Jianzhen (SI9) to alleviate poststroke shoulder pain [23]. Point combinations like Baihui (GV20) and Sishencong (EX‐HN1) are indicated for improving symptoms of vascular dementia (VaD) [24]. Beyond XNKQ, other specialized acupuncture methods have shown promise. The “Temporal Three‐Needle” technique, for instance, developed by Professor Jin Rui, has demonstrated significant therapeutic effects in managing various postischemic stroke sequelae [25]. The Sanjiao acupuncture method has been linked to suppressing oxidative stress and improving cognitive function [26, 27], while the Tiaoshen Jieyu method has shown good efficacy in treating poststroke depression [28]. To support these therapeutic applications, the research team has contributed by systematically mapping and illustrating human acupoint localizations in a standardized anatomical atlas (Figure 1).

Building upon traditional manual acupuncture techniques, electroacupuncture (EA) represents a further development where electrical stimulation (ES) is applied to the inserted needles at specific acupuncture points. EA is frequently reported to offer enhanced therapeutic efficacy compared to manual acupuncture (MA) alone [29, 30]. Research has also delved into optimizing EA parameters, with one study investigating the impact of electrode configuration on therapeutic outcome for IS, suggesting that positioning the anode over the affected hemisphere might potentiate the neuroprotective effects [31]. Clinical evidence supporting the use of acupuncture for stroke is substantial (Table 1). Several multicenter clinical trials have rigorously confirmed the safety and efficacy of acupuncture intervention during both the acute and subacute phases of IS. These trials consistently demonstrate that acupuncture contributes to reducing long‐term mortality and disability rates, while significantly improving overall neurological function [177, 178, 179]. Furthermore, numerous randomized controlled trials have provided evidence that acupuncture is effective in promoting recovery from a range of poststroke sequelae, including but not limited to ataxia, muscle weakness, limb pain, dysphagia, aphasia, and disorders of consciousness. These trials also indicate a potential reduction in mortality rates associated with acupuncture treatment [23, 180]. At the mechanistic level, multiple systematic reviews and animal studies have illuminated how EA exerts its effects. Compared to control groups, EA has been shown to enhance critical biomarkers associated with crucial recovery processes such as synaptic function, neurogenesis (the creation of new neurons), angiogenesis (the formation of new blood vessels), and neurotrophic factor activity (proteins supporting neuronal survival and growth) [181]. Moreover, when compared to conventional neuroprotectants, acupuncture is posited to offer multi‐target neuroprotection. This is achieved through enhancing endogenous neuroprotective mechanisms, mitigating cerebral edema, and reducing the volume of cerebral infarction, all contributing to improved neurological recovery [182]. Collectively, these findings highlight acupuncture, and particularly EA, as a promising complementary therapy. Therefore, this review aims to comprehensively explore the role of acupuncture in IS treatment, critically evaluate its proposed therapeutic mechanisms, assess its potential clinical benefits, and discuss its feasibility as an adjunct to conventional medical management.

**TABLE 1:** List of recent studies on EA stimulation for IS.

References	Acupuncture point	Pathway	Conclusion	Combination therapy	Subjects	EA/MA	Parameters
Song Z et al. [15]	Shuigou (GV26) Neiguan (PC6) Sanyinjiao (SP6) Temporal three needles	—	Improve basic activities of daily living	—	Human	MA	—
Zhang W et al. [20]	Shuigou (GV26) Neiguan (PC6) Sanyinjiao (SP6)	VEGF/Notch signaling pathway	Promote angiogenesis	—	Wistar rats	EA	2/15 Hz distant dense wave, 1 mA, 20 min/day, 7 days
Chen D et al. [21]	Baihui (GV20) Fengchi (GB20)	Restore telomerase reverse transcriptase	Reduce infarct volume, attenuate neuronal apoptosis and senescence, inhibit excessive production of inflammatory cytokines, and alleviate oxidative stress. Mitigate motor dysfunction while improving spatial learning and memory capabilities	—	Sprague–Dawley rats	EA	2/15 Hz, 1–3 mA, 30 min/day, 14 days
Qing L et al. [22]	Main points: Lianquan (CV23) Yifeng (SJ17) Adjunct points: Fengchi (GB20) Fengfu (GV16) Wangu (GB12) Lieque (LU7) Jinjin (EX‐HN12) Yuye (EX‐HN13)	—	Improve dysphagia	—	Human	EA	15–20 Hz, 5 mA, 30 min/day, 6 days/week, 4 weeks
Cheng J et al. [24]	Baihui (GV20) Sishencong (EX‐HN1)	BDNF/TrkB signaling pathway	Reduce neuroinflammatory responses and promote the restoration of synaptic plasticity, thereby improving learning and memory functions	—	Sprague–Dawley rats	EA	2/10 Hz, 1 mA, 30 min/day, 7 days
Ding YJ et al. [30]	Jianyu (LI15) Jianliao (TE14) Quchi (LI11) Shousanli (LI10)	—	Alleviate shoulder pain	—	Human	EA	15 Hz continuous wave and 2/100 Hz disperse‐dense wave, 30 min/day, 5 days/week, 4 weeks
Liu CH et al. [31]	Tongtian (BL7) Luoque (BL8)	Glutamate‐GABA balance pathway	Promotes nerve repair	—	Sprague–Dawley rats	EA	15 Hz, 20 min/day, 3 days
Shin S et al. [23]	Hegu (LI4) Jianyu (LI15) Jianliao (TE14) Jianzhen (SI9) Tianzong (SI11) Jianjing (GB21)	—	Improves shoulder pain	—	Human	EA	30 Hz, 20 min/day, 3 days/week, 3 weeks
Wang JH et al. [32, 33, 34]	Anterior oblique line of vertex‐temporal (MS6)	Upregulates IκB expression, inhibits NF‐κB dissociation, upregulates IL‐10 expression, and reduces IL‐6, IL‐1β, and TNF‐α expression	Reduces inflammatory response	—	Sprague–Dawley rats	MA	
Zhang SY et al. [35]	Anterior oblique line of vertex‐temporal (MS6)	Regulates the balance between CYP27a1/b1 and CYP24a1, converts vitamin D into active vitamin D3, inhibits vitamin D3 degradation, and modulates Th1/Th2 balance	Alleviates inflammatory response	—	Sprague–Dawley rats	EA	2/100 Hz, 1 mA, 30 min/day, 7 days
Xie L et al. [36]	Baihui (GV20)	Inhibits the NF‐κB pathway and activates the STAT6 pathway	Inhibits inflammatory response and enhances recognition ability and memory	—	Sprague–Dawley rats	EA	20 Hz, 20 mA, 2–4 V, 30 min/day, 6 days/week, 3 weeks
Yuan B et al. [37]	Anterior oblique line of vertex‐temporal (MS6)	Downregulates RORγt expression and promotes Th17/Treg balance via IL‐17A/FOXP3 modulation	Reduces cerebral infarction volume and improves neurological function and behavioral responses	—	Sprague–Dawley rats	EA	2/100 Hz, 1 mA, 30 min/day, 7 days
Xu A et al. [38]	Baihui (GV20) Shuigou (GV26)	Inhibits STAT3 activation and suppresses NK cell infiltration and activation	Reduces inflammatory response	—	C57BL/6 mice	EA	4/20 Hz sparse/dense wave, 1–3 V, 1–3 mA, 20 min/day, 3 days
Rao R et al. [39]	Baihui (GV20) Shuigou (GV26) Neiguan (PC6) Sanyinjiao (SP6)	PI3K/AKT/NF‐κB signaling pathway	Reduces pro‐inflammatory cytokine expression and promotes anti‐inflammatory factors	—	Sprague–Dawley rats	EA	2 Hz, 1 mA, 30 min/day, 7 days
Ye T et al. [40]	Baihui (DU20)	TLR4/NF‐κB/TXNIP/NLRP3 signaling pathway	Alleviates neurological deficits and neuronal apoptosis, suppresses inflammation, and maintains blood–brain barrier integrity	—	Sprague–Dawley rats	EA	2/15 Hz, 1 mA, 30 min/day, 6 day/week, 2 weeks
Ding H et al. [41]	Baihui (GV20) Fengfu (GV16) Neiguan (PC6) Xinshu (BL15)	HMGB1/TLR4 signaling pathway	Suppresses inflammatory injury and ameliorates neurological dysfunction	—	Sprague–Dawley rats	EA	5/100 Hz disperse‐dense wave, 0.5 mA, 20 min/day, 7 days
Wang M et al. [42]	Baihui (GV20) Fengfu (GV16) Fengchi (GB20)	—	Reduces cerebral infarction volume, improves regional cerebral blood flow and vascular integrity, restores blood–brain barrier function, and modulates neuroinflammation.	—	Sprague–Dawley rats	EA	3 Hz, 1 mA, 30 min/day, 3 days
Pan XR et al. [43]	Baihui (DU20)	Inhibits glycolysis, downregulates lactate production, and suppresses the formation of lactate‐derived protein KLA	Increases neuronal survival rate, inhibits astrocyte activation, and reduces cerebral infarction volume	—	C57BL/6J mice	EA	2/15 Hz, 1.5 mA, 30 min/day, 5 days
Lin X et al. [44]	Baihui (GV20) Hegu (LI4)	Upregulates CYLD, inhibits NLRP3 inflammasome activation, and modulates the CX3CL1/CX3CR1 axis	Exerts anti‐inflammatory effects and provides neuroprotection	—	Sprague–Dawley rats	EA	20 Hz, 1 mA, 5 min; 2 Hz, 30 min.qd
Xu H et al. [45]	Baihui (GV20) Hegu (LI4) Taichong (LR3)	Upregulates OTULIN expression and inhibits the NF‐κB signaling pathway	Alleviates brain injury and suppresses glial cell activation	—	Sprague–Dawley rats	EA	2/20 Hz, 1 mA, 30 min/day, 7 days
Ren X et al. [46]	Baihui (GV20) Shuigou (GV26)	Inhibits the TRPV4 channel	Inhibits neuroinflammation	—	C57BL/6J mice	EA	4/20 Hz, 1–3 mA, 1–3 V, 20 min/day, 3 days
Chen S et al. [47]	Shuigou (GV26)	Lnc826‐mediated Hippo pathway	Modulates microglial polarization	—	Sprague–Dawley rats	EA	4/20 Hz, 1–3 mA, 1–3 V, 30 min
Liao YS et al. [48]	Baihui (GV20) Zusanli (ST36)	Enhances ABCA1‐mediated exocytosis in M2‐polarized microglia	Inhibits microglial activation, promotes M2 polarization, and enhances phagocytic capacity for damaged neurons	—	C57BL/6J mice	EA	2/15 Hz, 1 mA, 20 min/day, 5 days
Jiao BQ et al. [49]	Baihui (GV20) Dazhui (GV14)	—	Upregulates M2 microglial markers, increases anti‐inflammatory factors, reduces pro‐inflammatory cytokines, and alleviates inflammation	—	C57BL/6 mice	EA	2 Hz, 0.5 mA, 20 min/day, 7 days
Yao Z et al. [50]	Chize (LU5) Hegu (LI4) Sanyinjiao (SP6) Zusanli (ST36)	STAT6/PPARγ pathway	Alleviates microglia‐associated neuroinflammation	—	Sprague–Dawley rats	EA	5 Hz, 2 mA, alternating sparse and dense waveforms (1.5 s each), 20 min/day, 7 days
Chen Y et al. [51]	Baihui (GV20)	Enhances SCFA‐mediated Foxp3 acetylation in Treg cells	Alleviates poststroke brain and gut inflammatory injury	—	Sprague–Dawley rats	EA	2/15 Hz, 1 mA, 30 min/day, 4 days
Zhang Q et al. [52]	Baihui (GV20) Zusanli (ST36)	Modulates gut immunity through the MGBA	Reduces postischemic brain and colonic damage and counteracts inflammation	Induced pluripotent stem cells (iPSCs)	C57BL/6 mice	EA	2 Hz, 1 mA, 30 min/day, 4 days
Li S et al. [53]	Baihui (GV20) Dazhui (GV14) Zusanli (ST36)	The gut microbiota activates melatonin receptors by modulating IPA	Exerts antioxidant effects and inhibits apoptosis		C57BL/6 mice	EA	2–15 Hz, 2 mA, 30 min/day, 5 days
Wang Y et al. [54]	Baihui (GV20)	Regulation of Tregs and γδ T Cells in the ischemic brain and small intestine	Inhibition of inflammation	—	Sprague–Dawley rats	EA	2/15 Hz, 1 mA, 20 min/day, 4 days
Wang YL et al. [55]	Baihui (GV20)	Regulation of Treg/γδ T cell polarization shift	Improvement of intestinal barrier integrity and resistance to inflammatory injury	—	Sprague–Dawley rats	EA	2/15 Hz, 1 mA, 20 min/day, 2 days
Zhang TS et al. [56]	Baihui (GV20) Shuigou (GV26)	Reduction of Glu and Asp levels in the cerebral infarct area	Reduction of excitatory amino acid neurotoxicity	—	Sprague–Dawley rats	EA	2 Hz, 3.5 mA, 60 min
Zhang Y et al. [57]	Baihui (GV20) Shenting (GV24)	Inhibition of Glu neurotoxicity and suppression of Ca^2+^ influx via downregulation of NMDAR2B expression	Reduction of infarct volume and improvement of cognitive impairment	—	Sprague–Dawley rats	EA	1–20 Hz, 30 min/day, 7 days
Wang J et al. [58]	Jianyu (LI15) Waiguan (TE5) Qinglengyuan (TE11) Lingdao (HT4) Midpoint of Tianquan (PC2) and Quze (PC3) Midpoint of PC3 and Xiagmen (PC4)	Reduction of serum NO levels, plasma ET, and brain Ca^2+^ elevation	—	—	Sprague–Dawley rats	EA	10 Hz, 0.6 ms, 1.5–3 V, 10 min/day, 3 days
Liu Z et al. [59]	Baihui (GV20)	Upregulation of GluR2 via cannabinoid CB1R and reduction of Bax/Bcl‐2 ratio	Inhibition of neuronal apoptosis	—	C57BL/6 mice	EA	2/15 Hz, 1 mA, 30 min
Guo Z et al. [60]	Baihui (GV20)	Promotion of GLT‐1 expression and inhibition of Glu release	Neuroprotection	—	Sprague–Dawley rats	EA	2/15 Hz, 30 min/day, 5 days
Liu J et al. [61]	Shuigou (GV26)	Maintenance of endogenous GABA inhibitory activity and reduction of excessive Glu release	Alleviation of brain injury	—	Sprague–Dawley rats	MA	
Gan P et al. [62]	Baihui (GV20) Shuigou (GV26)	Upregulation of GABA expression	Reduction of ischemic injury area	—	Sprague–Dawley rats	EA	3.58–6.25 Hz, 1.4–2.0 mA, EA stimulation started 15 min after IS: 30 min on, 10 min off, then 30 min on again (total 60 min)
Zhang X et al. [26]	Shanzhong (CV17) Zhongwan (CV12) Qihai (CV6) Xuehai (SP10) Zusanli (ST36)	Enhanced SOD activity (total, CuZnSOD, MnSOD), reduced oxidative markers (MDA, superoxide anion), improved mitochondrial redox status (GSH/GSSG ratio), and boosted bioenergetic efficiency (RCI, P/O ratio, respiratory enzyme activity) in MID rats	Increase in cerebral blood flow, improvement of mitochondrial dysfunction induced by ischemia and endogenous cerebral oxidative stress system, and enhancement of cognitive function	—	Wistar rats	MA	
Su X et al. [63]	Baihui (GV20) Dazhui (GV14) Shuigou (GV26) Fengfu (GV16)	Inhibition of TOMM40 and TIMM17A function suppresses mitochondrial Aβ accumulation, blocks pro‐oxidative factors (NO/iNOS), and restores ATP, SOD, and COX production	Improvement of cognitive impairment	—	Sprague–Dawley rats	MA	
Liu CZ et al. [27]	Shanzhong (CV17) Zhongwan (CV12) Qihai (CV6) Xuehai (SP10) Zusanli (ST36)	Increase in SOD and GSH‐PxActivity, and enhancement of CuZnSOD mRNA and protein expression	Improvement of oxidative damage and enhancement of memory impairment	—	Wistar rats	MA	
Shen MH et al. [64]	Baihui (GV20) Dazhui (GV14)	Upregulation of γ‐GCS Protein, γ‐GCS_h mRNA, and γ‐GCSI mRNA expression levels	Elimination of excessive ROS and protection of brain cells	—	Sprague–Dawley rats	EA	3 Hz, 1–3 mA, 30 min
Ni C et al. [65]	Baihui (GV20)	Activation of the Keap1‐independent GSK‐3β/Nrf2 signaling pathway	Antioxidant stress	—	C57BL/6J WT mice	EA	2/15 Hz, 1 mA, 30 min/day, 7 days
Xue X et al. [66]	Zusanli (ST36) Quchi (LI11)	Activation of the PI3K/Akt signaling pathway. Increased expression of PI3K, p‐Akt, p‐Bad, and Bcl‐2 at the protein level, and increased expression of Bcl‐2 at the mRNA level. Inhibition of Bax and cleavage of Caspase‐3 positive expression	Inhibition of cell apoptosis	—	Sprague–Dawley rats	EA	Disperse wave of 4 and 20 Hz, 30min/day, 3 days
Xing Y et al. [67]	Zusanli (ST36) Quchi (LI11)	Upregulation of Midkine (MK) and ERK/JNK/p38 signaling pathway, increased Bcl‐2, and decreased caspase‐3 and Bim.	Inhibition of neuronal apoptosis	—	Sprague–Dawley rats	EA	4/20 Hz, 1 mA, 6 V, 30 min/day, 3 days
Wang T et al. [68]	Shanzhong (CV17) Zhongwan (CV12) Qihai (CV6) Zusanli (ST36) Xuehai (SP10)	Increased Bcl‐2 and decreased Bax	Anti‐apoptotic	—	Wistar rats	MA	
Chen A et al. [69]	Zusanli (ST36) Quchi (LI11)	Increase in serum secretion levels of PI3K activators BDNF and GDNF, upregulation of the PI3K/Akt signaling pathway, and upregulation of the Bcl‐2/Bax ratio	Inhibition of brain cell apoptosis	—	Sprague–Dawley rats	EA	Disperse wave of 1 and 20 Hz, 30 min/day
Liu F et al. [70]	Baihui (GV20) Shenting (GV24)	Significant increase in the Bcl‐2/Bax ratio	Improvement of cognitive function	—	Sprague–Dawley rats	EA	1–20 Hz, 6 mA, 30 min/day, 14 days
Wang B et al. [71]	Baihui (GV20) Shenshu (BL23) Sanyinjiao (SP6)	Inhibition of p53 and caspase‐3 expression	Resistance to neuronal apoptosis	—	Sprague–Dawley rats	EA	Disperse‐dense wave, 2 Hz/100 Hz, 1 mA, 10 min on, 5 min off, then repeated four times (total 60 min)
Zhang Y et al. [72]	Baihui (GV20) Sishencong (EX‐HN1)	Increase in Bcl‐2 expression levels, downregulation of Bax, Caspase‐3, and Caspase‐9 expression levels, and increase in GDNF and BDNF expression levels	Inhibit apoptosis and protect nerves	—	Sprague–Dawley rats	MA	
Meng L et al. [73]	Baihui (GV20)	By enhancing the differential enrichment of histone acetylation (H3K9ac/H3K27ac) at gene promoter regions, bidirectional regulation of apoptosis‐related gene expression occurs. The occupancy of H3K9ac/H3K27ac at the Bcl‐2 promoter region is increased, leading to elevated Bcl‐2 expression; while the enrichment of H3K9ac/H3K27ac at the Caspase‐3 and Bax promoter regions is reduced, resulting in decreased Caspase‐3/Bax expression	Inhibition of cell apoptosis	—	Sprague–Dawley rats	EA	2/15 Hz, 1 mA, 30 min/day
Long M et al. [74]	Baihui (GV20) Shenshu (BL23) Sanyinjiao (SP6)	By downregulating TRPV1 expression, inhibiting the NF‐κB pathway, reducing Bax and Caspase‐3, and increasing Bcl‐2	Anti‐apoptotic, anti‐inflammatory, and neuroprotective effects	—	Sprague–Dawley rats	EA	2/100 Hz, 1 mA, 10 min on, 5 min off, then repeated four times (total 60 min)
Fang H et al. [75]	Baihui (GV20) Dazhui (GV14)	Inhibition of the RhoA/Pyrin/GSDMD signaling pathway	Inhibition of microglial pyroptosis	—	Sprague–Dawley rats	EA	3 Hz, 1–2 V, 30 min/day, 7 days
Tang B et al. [76]	Baihui (GV20) Zusanli (ST36)	Inhibition of the NLRP3/ASC/Caspase‐1/GSDMD signaling pathway and reduction of IL‐1β expression	Inhibition of pyroptosis and neuroprotection	—	Sprague–Dawley rats	EA	20 Hz, 30 min/day, 7 days
Zhang X et al. [77]	Baihui (GV20) Neiguan (PC6)	By activating the SIRT1/FOXO1 signaling pathway, promoting Beclin‐1 and SOD, and inhibiting ROS and MDA	Promotion of autophagy, antioxidant effects, inhibition of oxidative stress and neuronal damage, and improvement of cognitive function	—	Sprague–Dawley rats	EA	2/100 Hz, 1 mA, 30 min/day, 5 days
Wu Z et al. [78]	Baihui (GV20)	Reduction of LC3‐IIH and Beclin 1, inhibition of excessive autophagy	Regulation of autophagy levels and improvement of neurological function	—	Sprague–Dawley rats	EA	2/15 Hz, 1 mA, 30 min/day, 5 days
Ting Z et al. [79]	Baihui (GV20) Mingmen (GV4) Zusanli (ST36)	Downregulate LC3‐II and Beclin1, upregulate mTOR, and inhibit excessive autophagy. Upregulate SOD, downregulate MDA and IL‐6, exerting anti‐inflammatory and antioxidant effects	Inhibit excessive autophagy, improve mitochondrial function, and reduce neuronal death	—	Sprague–Dawley rats	EA	40–50 Hz
Mei ZG et al. [80]	Baihui (GV20) Zusanli (ST36) Quchi (LI11)	Activate the SIRT1‐FOXO1 signaling pathway and inhibit Atg7 and LC3‐II	Inhibit excessive autophagy and protect nerves	—	Sprague–Dawley rats	EA	2/15 Hz, 1 mA, 30 min/day, 5 days
Liu W et al. [81]	Zusanli (ST36) Quchi (LI11)	Suppress the mTORC1‐ULK complex‐Beclin1 pathway	Inhibit autophagosome formation and autophagy	—	Sprague–Dawley rats	EA	1–20 Hz, 0.2 mA, 30 min/day, 3 days
Tang H et al. [82]	Baihui (GV20) Dazhui (GV14) Shuigou (GV26)	Activate the class III PI3K/Beclin‐1 pathway, upregulate Beclin‐1, LC3B‐II, and Lamp2, and downregulate P62	Regulate autophagy and protect nerves	—	Sprague–Dawley rats	MA	
Lu XY et al. [83]	Baihui (GV20) Dazhui (GV14) Shuigou (GV26)	Upregulate miR‐34c‐5p expression	Promote neuronal autophagy and anti‐apoptosis	—	Sprague–Dawley rats	MA	
Yashuo F et al. [84]	Zusanli (ST36)	Reduce FIS1 and Drp1 expression levels and decrease inflammatory factors (IL‐1β, TNF‐α, IL‐6, and IL‐8)	Improve motor dysfunction and alleviate neuronal mitochondrial damage	—	C57BL/6J mice	EA	15 Hz, 15 min/day, 3 days
Xing Y et al. [85]	Baihui (GV20) Zusanli (ST36)	Inhibit LC3‐II, Beclin1, and CCAS3, while promoting LAMP1, SIRT1, and ERK	Regulate autophagy, resist apoptosis, and reduce cerebral edema and neuronal death	—	Sprague–Dawley rats	EA	4/20 Hz, 4 V, 30 min
Chen C et al. [86]	Baihui (GV20)	Dynamically regulate autophagy via the Wnt/GSK3β‐mTOR axis. Wnt pathway activation induces GSK3β phosphorylation (inactivation) and mTOR activation, upregulating β‐catenin and LC3‐II while downregulating p62	Maintain autophagy balance and protect nerves	—	Sprague–Dawley rats	EA	2–15 Hz, 1 mA, 30 min
Wang H et al. [87]	Baihui (GV20) Zusanli (ST36)	Inhibit peroxynitrite (ONOO^−^)‐induced mitochondrial permeability transition pore (mPTP) opening and promote Pink1/Parkin‐mediated mitophagy	Regulate autophagy, clear damaged mitochondria, and improve neuronal injury	—	Sprague–Dawley rats	EA	20 Hz, 30 min
Wang GL et al. [88]	Baihui (GV20) Shuigou (GV26)	Reduce ACSL4 and TFR1 expression while increasing GPX4 levels	Inhibit ferroptosis	—	Sprague–Dawley rats	EA	3.85 Hz/6.25 Hz, 0.8–1.3 mA, 1.28 s/2.08 s, 30 min/day, 3 days
Zhu W et al. [89]	Baihui (GV20) Shuigou (GV26) Neiguan (PC6) Sanyinjiao (SP6)	Promote Nrf2 nuclear translocation and increase SLC7A11 and GPX4 protein expression	Inhibit ferroptosis	—	Sprague–Dawley rats	EA	2/100 Hz, 0.5–1.5 mA, 2–4 V, 30 min/day, 7 days
Yang XC et al. [90]	Baihui (GV20) Dazhui (GV14) Quchi (LI11) Neiguan (PC6)	Facilitate Nrf2 nuclear translocation, activate the Nrf2/SLC7A11/GPX4 pathway, upregulate SOD and FTH1, and downregulate ACSL4, ROS, and MDA	Inhibit ferroptosis	—	Sprague–Dawley rats	EA	2 Hz, 2 mA, 30 min/day, 7 days
Li N et al. [91]	Baihui (GV20) Shuigou (GV26) Zusanli (ST36) Neiguan (PC6)	Upregulate xCT expression and activate the system Xc/GSH/GPX4 axis	Inhibit ferroptosis	—	Sprague–Dawley rats	EA	Sparse‐dense waves, 1–2 mA, 20 times/min, 15 min/day, 7 days
Ma L et al. [92]	Baihui (GV20) Dazhui (GV14)	Activate SIRT5 to induce NEK7 protein desuccinylation at the K81 site, thereby downregulating NEK7 expression	Inhibit neuronal pyroptosis	—	C57BL/6 mice	EA	Continuous wave, 2 Hz, 1 mA, 30 min/day, 3 days
Pu Y et al. [93]	Baihui (GV20) Dazhui (GV14) Shuigou (GV26)	Inhibit Keap1 to promote Nrf2 nuclear translocation, suppress ROS, LPO, pro‐inflammatory factors, Fe^2+^, and NLRP3, and enhance HO‐1, NQO1, and GPX4	Suppress oxidative stress, inflammatory response, ferroptosis, pyroptosis, and apoptosis	—	Sprague–Dawley rats	EA	2/15 Hz, 1 mA, 30 min/day, 5 days
Li G et al. [94]	Baihui (GV20) Shuigou (GV26) Neiguan (PC6) Sanyinjiao (SP6)	Promote GPX4 and FTH1 expression, increase SOD and GSH, and inhibit MDA, Fe^2+^, and TfR1	Improve motor function, reduce cerebral infarction volume, regulate oxidative stress and iron metabolism, and protect nerves	—	Sprague–Dawley rats	EA	2/100 Hz, 2–4 V, 30 min/day, 7 days
Jung YS et al. [95]	Baihui (GV20) Dazhui (GV14)	Suppress the NOX4/ROS pathway, upregulate tight junction proteins (ZO‐1, claudin‐5), and downregulate AQP4, Evans blue leakage, brain water content, ROS, and NOX4	Protect blood–brain barrier integrity and alleviate cerebral edema	—	C57BL/6J mice	EA	The intensity was maintained just below the level that induced visible muscle contraction, 20 min/day, 3 days
Zou R et al. [96]	Baihui (GV20)	Inhibit p‐caveolin‐1 and activate Akt signaling	Reduce blood–brain barrier permeability and cerebral edema	—	Sprague–Dawley rats	EA	2/15 Hz, 1 mA, 30 min/day, 5 days
Yao XQ et al. [97]	Anterior oblique line of vertex‐temporal (MS6)	Upregulate striatal PTX3, ZO‐1 mRNA, and Occludin mRNA expression, inhibit IL‐1β	Alleviate blood–brain barrier damage and neurological deficits	—	Sprague–Dawley rats	MA	
Lin R et al. [98]	Baihui (GV20) Shenting (GV24)	Suppress MMP‐2/MMP‐9 expression	Mitigate blood–brain barrier disruption and neuronal ultrastructural damage, improve neurological function, cerebral infarction volume, and learning‐memory ability, protecting nerves	—	Sprague–Dawley rats	EA	1/20 Hz, 1–3 mA, 30 min/day, 7 days
Yonglin C et al. [99]	Baihui (GV20)	Regulate histone acetylation (H3K9ac/H3K27ac) to downregulate MMP‐9 and upregulate TIMP‐2 gene expression	Alleviate blood–brain barrier disruption and improve neurological function	—	Sprague–Dawley rats	EA	2/15 Hz, 1 mA, 30 min/day, 3 days
Zhang H et al. [100]	Baihui (GV20) Shuigou (GV26)	Reduce pericyte apoptosis and migration, enhance pericyte viability, and improve oxygen–glucose deprivation/reoxygenation (OGD/R). Maintain brain microvascular endothelial cell‐pericyte interaction and barrier function	Ameliorate blood–brain barrier injury	—	Sprague–Dawley rats	EA	3.85/6.25 Hz, 1 mA, 30 min/day, 3 days
Liu H et al. [101]	Shuigou (GV26) Neiguan (PC6)	Upregulate TIMP1/downregulate MMP9 to inhibit GSDMD‐mediated pyroptosis (reducing IL‐1β/IL‐18 release)	Protect the blood–brain barrier and inhibit pyroptosis	—	Sprague–Dawley rats	EA	2/15 Hz, 1 mA, 30 min
Qian K et al. [102]	Baihui (GV20) Shuigou (GV26)	Specific mode electroacupuncture stimulation (SMES) dynamically regulates BBB permeability via the “p65‐VEGFA‐tight junction” axis, activating p65 phosphorylation to promote VEGFA release, thereby downregulating tight junction proteins (Occludin/ZO‐1) and reversibly opening the BBB in the infarct border zone to enhance exogenous drug (e.g., NGF) delivery	Regulate blood–brain barrier permeability and provide a novel targeted drug delivery strategy	—	Sprague–Dawley rats	EA	2/100 Hz, 3 mA, 6 min on, 6 min off (total 40 min)
Du Y et al. [103]	Shuigou (GV26)	—	Promote angiogenesis	—	Wistar rats	EA	15 Hz, 0.1 mA, 20 min/day, 2/3/7/12 days
Shi L et al. [104]	Shuigou (GV26)	Upregulate von Willebrand factor (vWF) and the cell proliferation marker Ki67	Facilitate collateral circulation and angiogenesis, improving neurological function	—	Wistar rats	EA	15 Hz, 1 mA, 5 min/day, 1/3/7/12 days
Li J et al. [105]	Shuigou (GV26)	Inhibit the AT1R/Gq/CaM pathway, activate the AT2R pathway, and increase DAG/IP3	Reduce vasoconstriction and improve cerebral blood flow	—	Wistar rats	EA	15 Hz, 1 mA, 20 min
Zheng HZ et al. [106]	Shuigou (GV26) Neiguan (PC6)	Regulate specific miRNAs (rno‐miR‐206‐3p, 3473, 6216, 494‐3p) to significantly activate the VEGF signaling pathway	Enhance cerebral blood perfusion and neurological recovery	—	Wistar rats	EA	2 Hz, 3 mA, 1 min
Li C et al. [107]	Central region of quadriceps muscle	Upregulate HIF‐1α expression	Reduce cerebral infarction volume and improve neurological deficits	—	Sprague–Dawley rats	EA	50 Hz, 3 mA
Zhao Y et al. [108]	Baihui (GV20)	Activate the notch‐HIF‐1α signaling axis	Protect nerves	—	Sprague–Dawley rats	EA	2/15 Hz, 1 mA, 30 min/day, 5 days
Zan XC et al. [109]	Baihui (GV20) Shuigou (GV26) Neiguan (PC6)	Increase CD34+ microvessel density and upregulate pro‐angiogenic factors (VEGF, VEGFR2, bFGF)	Promote angiogenesis and restore cerebral blood flow	Rehabilitation training	Sprague–Dawley rats	EA	2/20 Hz, 3–5 V, 20 min/day, 14 days
Wang L et al. [110]	Baihui (GV20) Shuigou (GV26)	Activate the basal forebrain cholinergic pathway‐leptomeningeal collateral circulation axis, stimulate cholinergic neurons in the nucleus basalis of Meynert (NBM), and promote acetylcholine release	Enhance leptomeningeal collateral circulation and promote cerebral blood flow recovery	—	Sprague–Dawley rats	EA	—
Mao QJ et al. [111]	Baihui (GV20) Shuigou (GV26) Zusanli (ST36)	Ameliorate ultrastructural damage in ischemic cortical microvessels and upregulate VEGF mRNA in the ischemic area	Stimulate capillary angiogenesis and functional reconstruction of damaged microvasculature	—	Sprague–Dawley rats	EA	2/15 Hz, 1 mA, 30 min/day, 1/2/4/8 days
Xie C et al. [112]	Baihui (GV20) Hegu (LI4) Taichong (LR3) Siguan points (bilateral Hegu + Taichong)	Activate the SDF‐1α/EPCs axis, increase endothelial progenitor cell (EPC) counts in bone marrow (BM) and peripheral blood (PB), reshape the SDF‐1α concentration gradient (decreased in BM, increased in PB), and drive EPC homing to ischemic regions	Promote angiogenesis and improve neurological function	—	Sprague–Dawley rats	EA	2/20 Hz, 1 mA, 30 min/day, 1/2/3/7 days
Jia LY et al. [113]	Shuigou (GV26)	Upregulate angiogenic factors (bFGF, Ang‐1, Ang‐2, PDGF‐b) and activate multiple pathways (bFGF/Ang/PDGF‐b)	Vascular regeneration and collateral circulation reconstruction	—	Wistar rats	EA	15 Hz, 2 mA, 20 min/day, 1/3/7/12 days
Yang YY et al. [114]	Baihui (GV20) Zusanli (ST36)	Activate the HIF‐VEGF‐Notch pathway	Neuroprotection and vascular repair	—	Sprague–Dawley rats	EA	1/20 Hz, 1–2 mA, 30 min/day, 7 days
Cao L et al. [115]	Zusanli (ST36)	Neurons secrete irisin to activate VEGF/Akt/eNOS	Blood flow reconstruction and neurological recovery	—	Sprague–Dawley rats	EA	3/15 Hz, 30 min/day, 1/3/7/14 days
Si SH et al. [116]	Shuigou (GV26)	Epigenetically regulate (via miR‐142‐5p) to inhibit ADAMTS1, thereby activating the VEGF‐PI3K/AKT‐eNOS pro‐angiogenic pathway	Promote angiogenesis	—	Sprague–Dawley rats	EA	4/20 Hz, 30 min/day, 4 days
Shi S et al. [117]	A 1 cm diameter circle centered at Baihui (GV20), pierced by five needles at equidistant points with tips facing the center	Activate Wnt/β‐catenin signaling to promote VEGF and other angiogenic factors	Blood flow recanalization and neuroprotection	—	Wistar rats	EA	3–15 Hz, 2–4 mA, 30 min/day, 7 days
Xu SY et al. [118, 119]	Baihui (GV20) Shuigou (GV26)	Upregulate exosomal miR‐210 to activate the HIF‐1α/VEGF/Notch1 pathway	Promote neovascularization	—	Sprague–Dawley rats	EA	Density wave was applied with a sparse‐wave frequency of 3.85 Hz (duration: 1.28 s) and a dense‐wave frequency of 6.25 Hz (duration: 2.08 s), 0.8–1.3 mA, 30 min/day, 3 days
Wang L et al. [120]	Shuigou (GV26)	Promote EPO release, activate Src phosphorylation, upregulate p‐Src, and enhance VEGF expression	Facilitate vascular remodeling	—	Wistar rats	EA	15 Hz, 1 mA, 20 min
Li GD et al. [121]	Baihui (GV20) Shuigou (GV26)	Activate Wnt/β‐catenin to upregulate VEGF, NeuN, and GFAP	Enhance neuroprotection and vascular remodeling	—	Sprague–Dawley rats	EA	2/100 Hz, 2–4 V, 30 min/day, 21 days
Kim YR et al. [122]	Baihui (GV20) Dazhui (GV14)	Activate the BDNF/VEGF‐PI3K pathway to promote neural stem cell (NSC) proliferation and differentiation	Promote nerve regeneration and repair	—	C57BL/6 mice	EA	2 Hz, 2 V, 20 min/day, 10 days
Chen C et al. [123]	Tianquan (PC2) Quze (PC3) Neiguan (PC6) Daling (PC7) Tianfu (LU3) Chize (LU5) Lieque (LU7) Taiyuan (LU9)	Modulate the NGF/Nogo‐A pathway, upregulate serum and brain NGF, and downregulate serum Nogo‐A	Stimulate nerve regeneration	—	Sprague–Dawley rats	EA	20 Hz, 2–4 V, 30 min, 0/6/24/48/72 h
Luo D et al. [124]	Shuigou (GV26)	Increase Nestin/BrdU(+) cells and regulate the GSK‐3β/PP2A signaling pathway	Improve blood flow and promote endogenous nerve regeneration	—	Sprague–Dawley rats	MA	
Dong W et al. [125, 126]	Baihui (GV20) Zusanli (ST36)	Activate the Nogo‐A/NgR‐MBP pathway, increase MBP expression, downregulate Nogo‐A and NgR levels, and suppress Nogo‐A/NgR expression	Enhance myelin regeneration	—	Sprague–Dawley rats	EA	2/10 Hz, 1 mA, 30 min/day (2:00 PM), 2 weeks
Zhang S et al. [127]	Hegu (LI4) Zusanli (ST36)	Promote NeuroD1‐mediated neural stem cell differentiation via miR‐146b	Ameliorate nerve injury	—	Sprague–Dawley rats	EA	1/20 Hz, 1 mA, 30 min/day, 21 days
Song X et al. [128]	Baihui (GV20) Dazhui (GV14)	Upregulate miR‐381, inhibit LRRC4 expression, relieve suppression of the SDF‐1/CXCR4 pathway, and activate ERK1 signaling	Reduce inflammation and promote nerve repair	—	Sprague–Dawley rats	EA	2/10 Hz, 1 mA, 30 min/day, 14 days
Nie Z et al. [129]	Baihui (GV20) Zusanli (ST36)	Suppress the HMGB1/RAGE/p‐JNK signaling pathway	Alleviate neuroinflammation, reduce apoptosis, provide neuroprotection, and improve motor function	—	Sprague–Dawley rats	EA	2 Hz, 1 mA, 30 min/day, 14 days
Lee HJ et al. [130]	Sishencong (EX‐HN1)	Activate the BDNF/TrkB pathway to promote GSK3β phosphorylation and enhance TGF‐β/NT‐3 and NG2 cell differentiation	Improve motor dysfunction	—	C57BL/6 mice	EA	2 Hz, 1 mA/3 mA, 20 min/2 days, 3 or 6 sessions
Zhang Y et al. [131]	Neiguan (PC6) Zusanli (ST36)	Activate the mTOR pathway, upregulate phosphorylation of AKT, mTOR, S6, and PTEN, and enhance GAP‐43 and SYN expression	Facilitate corticospinal tract axonal sprouting at contralateral cortical and spinal (C1–C4) levels to induce endogenous neural remodeling, aiding nerve recovery	—	Sprague–Dawley rats	EA	2 Hz, 1 mA, 20 min/day, 14 days
Chen B et al. [132]	Quchi (LI11) Zusanli (ST36)	Promote SSH1 protein expression, inhibit LIMK1 expression, suppress cofilin rod formation, and reduce neuronal apoptosis	Improve neurological function	—	Sprague–Dawley rats	EA	4/20 Hz, 0.2 mA, 30 min/day, 7 days
Yin L et al. [133]	Quchi (LI11) Zusanli (ST36)	—	Enhance cortico‐striatal network function	Resting‐state functional magnetic resonance imaging (fMRI)	Sprague–Dawley rats	EA	1/20 Hz, 2 mA, 6 V, 30 min/day, 7 days
Yao LL et al. [134]	Baihui (GV20) Dazhui (GV14)	S1‐M1 neural circuit	Promote neuronal activity and functional connectivity	—	C57BL/6J mice	EA	Sparse wave: 2 Hz, dense wave: 10 Hz, 1 mA, 15 min
Xiao Y et al. [135]	Baihui (GV20) Shenting (GV24)	Activate the Nrf2/HO‐1 signaling pathway	Induce M2 polarization of microglia/macrophages and enhance learning‐memory function	—	Sprague–Dawley rats	EA	1–20 Hz, 6 V, 30 min/day, 14 days
Chen L et al. [136]	Baihui (GV20) Shenting (GV24)	Regulate the SIRT1/PGC‐1α—OPA1/DRP1 axis to enhance SIRT1, PGC‐1α, and OPA1 expression, inhibit DRP1, and promote mitochondrial fusion while blocking fission	Improve cognitive impairment and learning‐memory ability	—	Rats	EA	1–20 Hz, 6 V, 30 min/day, 14 days
Lin B et al. [137]	Baihui (GV20) Shenting (GV24)	Inhibit P2X7R expression, upregulate Nrf2, downregulate NLRP3, and promote microglial M2 polarization	Reduce inflammation and oxidative stress, ameliorating memory impairment	—	Sprague–Dawley rats	EA	2/20 Hz, 0.2 mA, 6 V, 30 min/day, 7 days
Wen Q et al. [138]	Baihui (GV20) Neiguan (PC6) Yongquan (KI1)	Suppress p38 MAPK phosphorylation in microglia	Improve learning and memory deficits	—	Sprague–Dawley rats	EA	1–20 Hz, 2 mA, 6 V, 30 min/day, 7 days
Su K et al. [139]	Baihui (GV20) Shenting (GV24)	Modulate the Pten/Akt pathway to increase Pak4, Akt3, and Efnb2 expression	Alleviate cognitive deficits	—	Sprague–Dawley rats	EA	1–20 Hz, 1–3 mA, 30 min/day, 7/14 days
Lin R et al. [140]	Baihui (GV20) Shenting (GV24)	Inhibit the NF‐κB signaling pathway	Enhance learning‐memory ability	—	Sprague–Dawley rats	EA	0.05 Hz, 6 V, 30 min/day, 7 days
Wang HL et al. [141]	Baihui (GV20) Shenting (GV24)	Activate the PI3K/Akt pathway, enhance Beclin‐1, mTOR, and PI3K expression, and reduce p53 levels	Enhance learning‐memory ability	—	Sprague–Dawley rats	EA	1–20 Hz, 2 mA, 6 V, 30 min/day, 8 days
Wang Z et al. [142]	Baihui (GV20) Shenting (GV24)	Activate the hippocampal 5‐HT1A receptor‐PKA pathway to enhance NMDA receptor function	Enhance learning‐memory ability	—	Sprague–Dawley rats	EA	2/20 Hz, 0.2 mA, 6 V, 30 min/day, 2 weeks
Lun X et al. [143]	Main points: Unilateral scalp reflex zone (CT‐localized) Supplementary points: Hegu (LI4) Zusanli (ST36) Ganshu (BL18) Shenshu (BL23)	—	Ameliorate vascular dementia	Traditional Chinese herbal medicine treatment	Human	EA	Dense‐disperse wave, frequency 14–26 times/min, 30 min/day. Two months constitute one treatment course (rest on Sundays), with a 10 day break before proceeding to the next course. A total of 2courses are administered.
Zhang W et al. [144]	Fengchi (GB20) Shuigou (GV26) Lianquan (CV23) Neiguan (PC6) Sanyinjiao (SP6) Yifeng (SJ17) Jinjin (EX‐HN12) Yuye (EX‐HN13)	—	Improve cerebral microcirculation and conduction velocity, enhance swallowing muscle motor function, and promote swallowing recovery	Motor‐swallowing rehabilitation	Human	EA	Continuous wave, 2 Hz, 30 min/day, 4 weeks
Zhang SY et al. [145]	EA: Fengchi (GB20) Jinjin (EX‐HN12) Yuye (EX‐HN13) MA: Lianquan (CV23) Tiantu (CV22)	—	Alleviate dysphagia and improve daily living ability	Swallowing rehabilitation training	Human	EA and MA	Waveform: biphasic square wave, Pulse width: 700 ms, charge: 1000 Ω, Amplitude: 0–25 mA (±10%), Stimulation mode: continuous contraction, Stimulation frequency: 1000 Hz, 30 min/day, 6 times/week, for 4 weeks (4 treatment courses)
Qi YJ et al. [146]	Fengchi (GB20) Tianzhu (BL10) Wangu (GB12) Lianquan (CV23) Pang Lianquan (Extra) Bloodletting: Jinjin (EX‐HN12) Yuye (EX‐HN13)	Increased levels of BDNF, NGF, and IGF‐1	Improve swallowing and respiratory function	—	Human	MA	
Wu Y et al. [147]	Xiaguan (ST7) Jiache (ST6) Chengjiang (CV24) Lianquan (CV23) Jinjin (EX‐HN12) Fengchi (GB20) Yifeng (TE17)	—	Restore swallowing function and significantly reduce complications such as aspiration, fever, and malnutrition	Postural control	Human	EA	Continuous wave, 5 Hz, 1 mA, 30 min per session, BID
Li T et al. [148]	Fengchi (GB20) Wangu (GB12) Yifeng (TE17) Three Tongue points (Extra) Neiguan (PC6) Tongli (HT5) Zusanli (ST36) Sanyinjiao (SP6)	—	Relieve dysphagia and reduce aspiration	Catheter balloon dilatation	Human	EA	5–8 Hz, 30 min/day, 5 times per week
Yu J et al. [149]	Lower 2/5 of anterior/posterior oblique lines of parietal and temporal regions Fengchi (GB20) Yiming (EX‐HN14) Gong point (Extra) Zhiqiang (Extra) Duanyan (Extra)	—	Improve swallowing function and quality of life	Neuromuscular electrical stimulation (NMES) and rehabilitation training	Human	MA	
Cai W et al. [150, 151]	Baihui (GV20) Sishencong (EX‐HN1) Sanyinjiao (SP6) Taichong (LR3) Ganshu (BL18)	—	Alleviate depressive symptoms and improve neurological function and daily living activity	—	Human	EA	2/100 Hz, 30 min
Yang Y et al. [152]	Baihui (GV20) Zusanli (ST36)	Increase VEGF and HIF‐1αexpression	Mitigate neurological deficits and improve muscle strength, sensorimotor function, postural symmetry recovery, and neurovascular regeneration	—	Sprague–Dawley rats	EA	2 Hz, 1 mA
Zhang K et al. [153]	Zusanli (ST36) Yinlingquan (SP9)	—	Normalize motor behavior	—	Gerbils	EA	20 Hz, 20 min
Tang X et al. [154]	Dazhui (GV14)	Bidirectionally modulate pyramidal neuron and PV neuron activity to restore NMDAR function	Restore motor cortex excitation–inhibition balance and improve sensorimotor function	—	C57BL/6J mice	EA	2/50/100 Hz, 1.0 mA, 30 min/day, 7 days
Hou Z et al. [155]	Hegu (LI4) Waiguan (TE5) Quchi (LI11) Jianyu (LI15) Taichong (LR3) Jiexi (ST41) Shangjuxu (ST37) Zusanli (ST36) Xuehai (SP10) Liangqiu (ST34) Futu (ST32) Biguan (ST31)	—	Improve motor dysfunction	Wet compress formula (WCF)	Human	EA	2 Hz, 30 min/day, 6 times per week, 4 weeks
Li SS et al. [156]	Quchi (LI11) Zusanli (ST36)	Alter functional resting‐state networks (RSNs), including the sensorimotor network (SMN), interoceptive network (IN), default mode network (DMN), and salience network (SN)	Facilitate motor function recovery	—	Sprague–Dawley rats	EA	2/15 Hz, 30 min/day, 7 days
Chen B et al. [157]	Baihui (GV20) Shenting (GV24)	Regulate mitochondrial translocation of cofilin	Reduce ischemic brain injury and apoptosis, improving motor and cognitive function	—	Sprague–Dawley rats	EA	4/20 Hz, 1 mA, 30 min
Wang D et al. [158]	Baihui (GV20) Zusanli (ST36)	Increase expression of Nogo‐A, P75NTR, NGF, BDNF, and VEGF	Promote motor function recovery	Constraint‐induced movement therapy	Sprague–Dawley rats	EA	100 Hz, 1 mA, 30 min/day, 14 days
Qing P et al. [159]	Quchi (LI11) Zusanli (ST36)	Enhance GAP‐43 and SYP expression in the hippocampal CA3 region	Enhance motor function recovery	Rehabilitation training	Sprague–Dawley rats	EA	5/10 Hz, 2 mA, 30 min/day, 2 weeks (rest on Sundays)
Duc Nguyen M et al. [160]	Baihui (GV20) Fengchi (GB20) Jianyu (LI15) Quchi (LI11) Hegu (LI4) Fengshi (GB31) Yanglingquan (GB34) Zusanli (ST36)	—	Improve muscle contraction and facilitate motor recovery	Bicycle training	Human	EA	50–100 Hz, 30 min/day, 5 times per week, 6 weeks
Sun X et al. [161]	Baihui (GV20) Qubin (GB7)	Suppress the NF‐κB/NLRP3 pathway and modulate the gut‐brain axis by increasing intestinal n‐propyl acetate and butyrate levels	Alleviate poststroke spasticity	—	Sprague–Dawley rats	EA	2–20 Hz, 30 min/day, 3 days
Ni SM et al. [28]	Baihui (GV20) Yintang (GV24) Neiguan (PC6) Taichong (LR3)	—	Improve depression severity, neurological function, daily living activity, and sleep quality in poststroke depression patients, with superior clinical efficacy to sertraline alone and reduced drug‐induced side effects	Sertraline hydrochloride tablets	Human	EA	2/100 Hz, 30 min/day, 6 times per week, 8 weeks
Cai W et al. [162]	Baihui (GV20) Yintang (GV24)	Activate the Shh pathway to reduce IL6 and TNFα, increase GSH, upregulate 5HT, and slightly decrease IL1β and malondialdehyde	Suppress inflammation and oxidative stress, effectively alleviating depressive‐like behaviors in poststroke depression (PSD)	—	Sprague–Dawley rats	EA	2 Hz, 30 min/day, 5 times per week, 28 days
Hu G et al. [163]	Baihui (GV20)	Activate CB1R and upregulate CB1R, NRF1, and TFAM expression	Promote mitochondrial biogenesis and improve depressive‐like behaviors and cognitive dysfunction	—	C57Bl6j mice	EA	2/15 Hz, 1 mA, 30 min/day, 14 days
Gao J et al. [164]	Hegu (LI4) Taichong (LR3)	Inhibit prefrontal cortical neuronal ferroptosis by reducing iron deposition, lipid peroxidation, and enhancing antioxidation	Reduce neurological deficits and enhance spontaneous activity/exploratory behavior in rats, improving depressive‐like behaviors	—	Sprague–Dawley rats	EA	2/100 Hz, 1 mA, 30 min, performed once for 2 weeks
Wang H et al. [165]	Benshen (GB13) Shenmen (HT7)	—	The combination of EA and PI may be an effective and safe therapeutic option for PSD	Psychological intervention (PI)	Human	EA	30 min/day, 5 times per week
Bu Y et al. [166]	Baihui (GV20) Zusanli (ST36)	Upregulate Bcl‐2 and downregulate Bax	Ameliorate cerebral ischemia	Oxygen therapy (OM)	Sprague–Dawley rats	EA	100 Hz, 3.5 mA, 30 min/day, 4 days
Li WQ et al. [167]	Baihui (GV20) Yintang (GV24) Quchi (LI11) Hegu (LI4) Zusanli (ST36) Sanyinjiao (SP6)	Decreased IL‐17 and increased IL‐10	Improve neurological function and daily living activities	Conventional Western medicine treatment	Human	EA	2/15 Hz, 30 min/day, 10 days
Dai M et al. [168]	Baihui (GV20) Shuigou (GV26)	—	Provide clinical evidence for the efficacy and safety of specific mode electroacupuncture stimulation (SMES) combined with NGF in stroke patients	Nerve growth factor (NGF)	Human	EA	2/100 Hz, 3 mA, 40 min
Dai M et al. [169]	Baihui (GV20) Shuigou (GV26)	Facilitate NGF delivery to the brain via the p65‐VEGFA‐TJs pathway	Enhance cognitive function	—	Sprague–Dawley rats	EA	2/100 Hz, 3 mA, 40 min (6 s on/off)
Wu F et al. [170]	Quchi (LI11) Hegu (LI4)	Downregulate Nogo‐A and NgR expression	Improve neurological dysfunction	Gastrodin	Sprague–Dawley rats	EA	2 Hz, 2 V, 1 ms, 30 min/day, 14 days
Li HB et al. [171]	Quchi (LI11) Hegu (LI4)	Increase Nestin and NSE‐IR‐positive cells in hippocampal CA1/CA3 regions, and reduce GFAP‐positive cells	Promote hippocampal mature neuron differentiation and proliferation, improving brain function	Gastrodin	Sprague–Dawley rats	EA	2 Hz, 2 V, 1 ms, 30 min/day, 14 days
Miao HC et al. [172]	Baihui (GV20) Zusanli (ST36)	Upregulate Nestin and stem cell factor (SCF) expression in the hippocampal DG	Facilitate neurological repair	Gastrodia polysaccharide (PGB)	Sprague–Dawley rats	EA	2 Hz, 3 V, 30 min/day, 2 weeks
Lee JH et al. [173]	Baihui (GV20) Dazhui (GV14)	—	Enhance hMSC survival, migration, and differentiation into neural lineages while suppressing astrocyte formation	Tenuigenin (TE), human mesenchymal stem cells (hMSCs)	C57BL/6 mice	EA	2 Hz, 2 V, 20 min/day, 22 days
Sun Z et al. [174]	Baihui (GV20) Dazhui (GV14)	—	Improve recovery from neurological deficits	Bone marrow stromal cells (BMSCs)	Sprague–Dawley rats	EA	3 Hz, 1 V, 15 min/day, 1 week
Deng P et al. [175]	Baihui (GV20) Zusanli (ST36)	Modulate the IL‐33/ST2 axis and suppress microglial/astrocytic activation	Neuroprotective effects	Induced pluripotent stem cell‐derived small extracellular vesicles (iPSC‐EVs)	C57BL/6 mice	EA	2 Hz, 1 mA, 30 min per session, 0/24/48 h
Li G et al. [176]	Zhigou (TE8)	Activate the superior and middle frontal gyrus (SMFG)	Improve aphasia	—	Human	EA	Bipolar electrical stimulation 2 Hz, each stimulation/rest cycle lasted 45 s

## 2 Exploring the Mechanisms of Electroacupuncture in Treating Ischemic Stroke

### 2.1 Spatiotemporal Framework of Electroacupuncture Mechanisms: Linking Pathophysiology and Neural Repair

Brain injury and repair following IS constitute a highly dynamic, multistage, and continuous process, in which the core pathophysiological features undergo fundamental shifts over time [183]. As a multimodal and multitarget intervention, the therapeutic mechanisms of EA are neither singular nor static. Instead, they closely align with the evolving pathological demands across different stages of stroke, exhibiting a strategic transition from early central and systemic neuroprotection to the promotion of endogenous neural repair and functional reorganization during the subacute and chronic phases.

#### 2.1.1 Dual Modes of Action: Central Neural Regulation and Systemic Modulation

The therapeutic effects of EA arise from the synergy and integration of central neural regulation and systemic modulation.

##### 2.1.1.1 Central Neural Regulation

Acupuncture‐evoked sensory signals are transmitted to the brainstem, thalamus, and cortical regions through somatosensory afferent pathways, such as the spinothalamic tract [184, 185]. EA directly intervenes in the ischemic core and penumbral regions through several mechanisms. These include modulating neuronal excitability [154], suppressing the excessive activation of glial cells (microglia and astrocytes) [186], and regulating the release of local neurotransmitters and neurotrophic factors [122, 130, 187], EA directly intervenes in the ischemic core and penumbral regions. For example, EA attenuates excitotoxicity by regulating N‐methyl‐D‐aspartate receptor function [56, 57] and alleviates local neuroinflammation by inhibiting the nuclear factor‐κB/nucleotide‐binding oligomerization domain‐like receptor protein 3 (NF‐κB/NLRP3) inflammasome axis [32, 33, 34], representing typical manifestations of its direct central neuroprotective effects.

##### 2.1.1.2 Systemic Modulation

EA also exerts systemic effects by regulating autonomic nervous system activity [188], the neuroendocrine–immune axis—such as the hypothalamic–pituitary–adrenal axis [189]—and the functional status of distal organs, including the gut. These regulatory effects alter peripheral immune cell phenotypes and recruitment (e.g., promoting regulatory T‐cell expansion [37]), reshape systemic inflammatory cytokine profiles, and influence the central immune microenvironment via the gut–brain axis [51, 52]. For instance, EA reduces neuroinflammation by increasing intestinal short‐chain fatty acid levels [51] and modulating the migration of gut γδ T cells [54, 55], exemplifying its systemic immunomodulatory actions. The relative contributions of these two modes vary across poststroke stages, and their dynamic crosstalk forms the foundation of EA's multidimensional therapeutic effects.

#### 2.1.2 Stage‐Dependent Evolution of Mechanisms and Clinical Implications

Aligned with the temporal trajectory of poststroke pathophysiological evolution, the dominant mechanisms of EA display pronounced stage‐specific characteristics, which are critical for guiding precise clinical application.

##### 2.1.2.1 Acute Phase (Hours to Days)

This phase is dominated by the explosive activation of ischemic cascades [183], including excitotoxicity, oxidative stress, inflammatory initiation, and multiple forms of programmed cell death (apoptosis, pyroptosis, and ferroptosis). Accordingly, the primary objective of EA at this stage is neuroprotection, with mechanisms focused on the rapid suppression of injury‐associated pathways. For example, EA activates the nuclear factor erythroid 2‐related factor 2/heme oxygenase‐1 antioxidant pathway [93], inhibits NF‐κB–mediated pro‐inflammatory signaling [32, 33, 34], and modulates the balance of B‐cell lymphoma‐2 family proteins to suppress apoptosis [66, 67, 68]. These effects aim to maximally preserve neurons within the ischemic penumbra, thereby laying the foundation for subsequent repair. Interventions at this stage emphasize the concept of a critical therapeutic time window.

##### 2.1.2.2 Subacute Phase (Days to Weeks)

As inflammatory responses reach their peak and begin to resolve, the brain enters a stage characterized by the clearance of injury‐associated debris and the initiation of reparative processes [190]. During this phase, the core objective of EA shifts toward the promotion and support of neural repair [191], with mechanistic emphasis transitioning from “damage suppression” to the “activation [122] of endogenous reparative programs.” These include the upregulation of vascular endothelial growth factor and associated signaling pathways—such as hypoxia‐inducible factor/vascular endothelial growth factor/Notch and Wnt/β‐catenin—to promote angiogenesis and vascular remodeling [111, 113]; enhancement of neurotrophic signaling pathways, including brain‐derived neurotrophic factor/tropomyosin receptor kinase B, to support neurogenesis, synaptogenesis, and remyelination [122, 125, 126, 130]; and the regulation of autophagy/mitophagy pathways to eliminate damaged organelles [87]. Concurrently, sustained systemic immunomodulation [190] and gut–brain axis regulation [51] by EA help establish a permissive systemic and local microenvironment for repair.

##### 2.1.2.3 Chronic Phase (Weeks to Months or Longer)

At this stage, lesion size stabilizes, and functional recovery relies predominantly on neural network remodeling and compensation [192, 193]. The primary goal of EA becomes the enhancement of neuroplasticity and functional reorganization, with mechanisms focusing on the modulation of brain‐network‐level functional connectivity and excitation–inhibition balance. Evidence indicates that EA enhances intra‐ and inter‐network connectivity efficiency within key resting‐state networks, such as the sensorimotor network and the default mode network [194, 195], facilitates compensatory activation of the contralesional hemisphere or ipsilesional spared regions [196], and modulates corticospinal tract excitability [131]. Moreover, through sustained neurotrophic support and anti‐inflammatory effects, EA provides the necessary molecular and cellular substrates for long‐term synaptic remodeling [197, 198] and circuit reorganization [133, 134].

**FIGURE 2:**
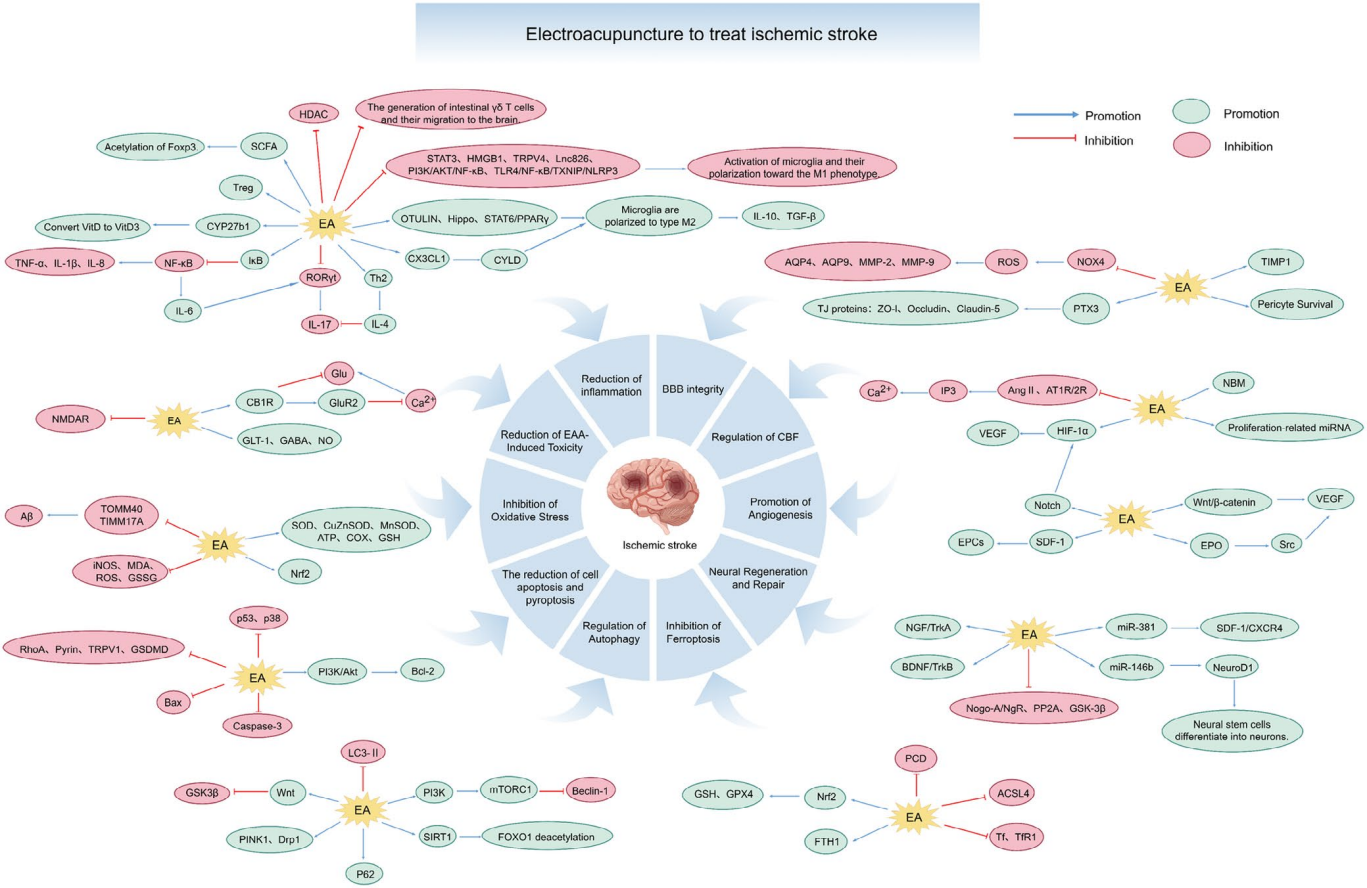
Electroacupuncture to treat IS. EA protects against IS by mitigating neuroinflammation, excitotoxicity, and oxidative damage while inhibiting multiple cell death pathways (apoptosis, pyroptosis, ferroptosis), regulating autophagy, preserving BBB integrity, improving CBF, and stimulating neurovascular repair.

Collectively, the mechanisms underlying EA treatment for IS constitute a dynamically evolving network that is tightly coupled to the natural disease course. During the acute phase, EA is dominated by central and systemic anti‐injury and pro‐survival effects. In the subacute and chronic phases, it transitions toward repair‐ and remodeling‐oriented strategies centered on angiogenesis, neurogenesis, and network reorganization. Recognizing this spatiotemporal heterogeneity in EA mechanisms is fundamentally important for the design of stage‐specific, individualized, and precision‐based therapeutic protocols, as well as for interpreting seemingly divergent findings—such as the bidirectional regulation of autophagy. Within this framework, the following sections will systematically delineate the molecular and cellular mechanisms by which EA engages specific pathological processes across different stages of IS (Figure 2).

### 2.2 Electroacupuncture Exerts Neuroprotective Effects in Ischemic Stroke

#### 2.2.1 Reduction of Inflammation

**FIGURE 3:**
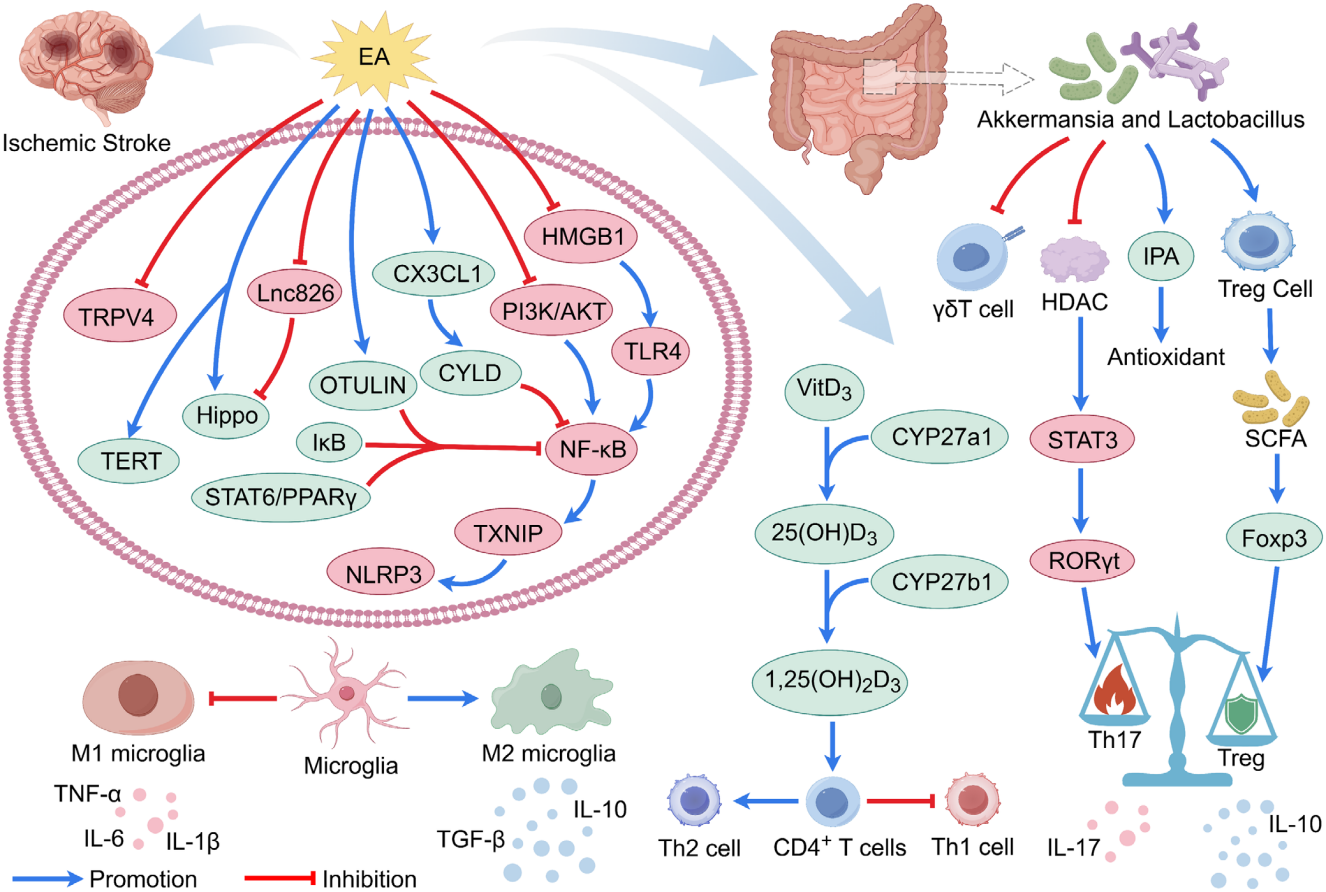
EA mitigates inflammatory responses in IS. EA promotes anti‐inflammatory M2 microglial polarization, increases the proportion of Th1 and Tregs, and modulates cytokine release by suppressing pro‐inflammatory factors while enhancing anti‐inflammatory mediators, thereby ameliorating neuroinflammation. Furthermore, EA demonstrates the capacity to mitigate neuroinflammatory responses via gut‐brain axis regulation.

One of the pivotal mechanisms through which EA exerts its therapeutic effects in IS is the modulation of inflammatory responses (Figure 3). Neuroinflammation is a critical component of secondary brain injury following IS, contributing significantly to tissue damage and functional deficits. Dysregulated clearance of apoptotic cells and the associated reprogramming of signaling pathways after IS drive neuroinflammation and tissue injury [199]. EA has been shown to effectively mitigate this inflammatory cascade through multiple pathways. A key target is the modulation of cytokine profiles. Acupuncture may reduce inflammation by inhibiting the activation of nuclear factor kappa B (NF‐κB) in brain tissue or peripheral blood. This inhibition leads to a decrease in the synthesis and secretion of major pro‐inflammatory cytokines, including interleukin 6 (IL‐6), interleukin 8 (IL‐8), interleukin 1 beta (IL‐1β), and tumor necrosis factor‐alpha (TNF‐α), while simultaneously promoting the secretion of the anti‐inflammatory cytokine interleukin 10 (IL‐10) [32, 33, 34]. Beyond systemic or broad pathway effects, EA also influences specific cellular components of the inflammatory response. For instance, EA suppresses the activation of glial cells, including microglia and astrocytes, and encourages the polarization of microglia toward the M2 phenotype, which is associated with tissue repair and anti‐inflammatory effects, thus reducing the detrimental overactivation of these cells and macrophages [36]. Meanwhile, ES may attenuate neuroinflammation and exert neuroprotective effects on neuronal cells [42].

Several studies have pinpointed specific molecular pathways involved in EA‐mediated microglial polarization and inflammatory regulation. EA has been shown to promote the polarization of microglia from the M1 (pro‐inflammatory) to the M2 (anti‐inflammatory) phenotype through the inhibition of the Phosphoinositide 3‐kinase (PI3K)/Protein kinase B (AKT)/NF‐κB signaling pathway [39]. EA preconditioning, particularly at GV20, may facilitate microglial polarization via the Toll‐like receptor 4 (TLR4)/NF‐κB/Thioredoxin‐interacting protein (TXNIP)/NOD‐like receptor family pyrin domain containing 3 (NLRP3) signaling pathway, leading to an increased population of M2‐type microglia and consequent mitigation of neurological impairments and neuronal apoptosis [40]. EA inhibits nuclear translocation of NF‐κB and enhances neuronal expression of C‐X3‐C motif chemokine ligand 1 (CX3CL1), suppressing microglial activation through upregulation of cylindromatosis (CYLD) in the ischemic cortex [44]. EA‐induced CYLD upregulation inhibits pro‐inflammatory M1‐like microglia and promotes anti‐inflammatory M2‐like microglia, contributing to neuroprotection and improved inflammatory balance [44]. Furthermore, EA increases the endogenous expression of OTU deubiquitinase with linear linkage specificity (OTULIN), which inhibits the activation of the NF‐κB pathway and reduces the secretion of TNF‐α, IL‐1β, and IL‐6, thereby suppressing microglia and astrocyte activation and exerting anti‐inflammatory and neuroprotective effects [45]. Other pathways implicated include the Signal transducer and activator of transcription 3 (STAT3) pathway, where EA stimulation at GV26 and GV20 mitigates postischemic brain infiltration and Natural killer (NK) cell activation by modulating STAT3 signaling [38]. EA may attenuate inflammatory injury by inhibiting High mobility group box 1 (HMGB1)/TLR4 signaling and downregulating pro‐inflammatory cytokine levels in models of focal cerebral ischemia (FCI) [41]. EA also activates the Signal transducer and activator of transcription 6 (STAT6)/Peroxisome proliferator‐activated receptor gamma (PPARγ) pathway and inhibits NF‐κB activity, further facilitating M2 microglial polarization and enhancing anti‐inflammatory cytokine secretion while suppressing M1 activation and pro‐inflammatory production [50]. Specific acupoint combinations also show unique effects; for instance, EA at GV26 and GV20 may attenuate neuroinflammation following cerebral ischemia–reperfusion injury (CIRI) by inhibiting microglial and macrophage activation and M1 polarization, associated with the downregulation of transient receptor potential vanilloid 4 (TRPV4) expression [46]. EA regulates microglial activation and cytokine production by inhibiting the upregulation of Lnc826 and modulating the Hippo signaling pathway, suppressing M1 polarization [47]. EA treatment may also alleviate neuronal cell death and excessive inflammation caused by CIRI through regulating exocytosis, potentially mediated by upregulation of ATP‐binding cassette transporter A1 (Abca1), which influences microglial activation, M2 polarization, and phagocytic capacity toward damaged neurons [48]. Pretreatment with EA at GV20 and Dazhui (GV14) has been shown to enhance M2 macrophage markers and increase levels of anti‐inflammatory factors like IL‐10 and transforming growth factor‐beta (TGF‐β), while reducing pro‐inflammatory factors such as serum IL‐1β [49]. Scalp EA approaches have also shown promise, potentially mitigating inflammatory responses by balancing Cytochrome P450 family 27 subfamily A1/B1 (CYP27A1/B1) and Cytochrome P450 family 24 subfamily A (CYP24A), enhancing active vitamin D3 conversion and modulating the T helper 1 (Th1)/T helper 2 (Th2) balance [35], and by downregulating Retinoic acid‐related orphan receptor gamma t (RORγt) to promote a balanced ratio of Interleukin 17A‐positive T helper 17 (IL‐17A+ Th17) cells to Forkhead box P3‐positive regulatory T (FOXP3+ Treg) cells [37]. Furthermore, EA stimulation at GV20 and GB20 may exert protective anti‐inflammatory effects by restoring telomerase reverse transcriptase (TERT) function, inhibiting excessive inflammatory activation [21]. EA pretreatment at GV20 may mitigate ischemic brain injury in mice with IS by inhibiting lactate production and the formation of lactylation of lysine (Kla) in derived proteins [43]. While diverse mechanisms are implicated, the overall effect is a significant reduction in the detrimental inflammatory cascade following IS.

Beyond direct effects within the central nervous system, accumulating evidence suggests that EA can also alleviate neuroinflammation by regulating the intricate communication along the gut‐brain axis. The metabolites produced by gut microbiota are increasingly recognized for their pivotal role in maintaining the functional homeostasis of the microbiota‐gut‐brain axis (MGBA), which in turn influences neuroprotection and neural tissue regeneration [200]. EA intervention has been shown to modulate this axis in ways that contribute to reduced inflammation. For example, stimulation of GV20 has been found to increase the number of regulatory T cells (Tregs) in the gut and enhance short‐chain fatty acid (SCFA)‐mediated Foxp3 acetylation. Concurrently, it inhibits histone deacetylase (HDAC) activity and suppresses the generation and migration of intestinal gamma delta T (γδ T) cells to the brain, thereby contributing to reduced inflammation in both the brain and the gut following IS [51]. Furthermore, studies investigating combined therapies, such as EA alongside induced pluripotent stem cell‐derived extracellular vesicles (iPSC‐EVs), have demonstrated modulation of gut immunity via the MGBA, characterized by downregulation of IL‐17 expression and upregulation of IL‐10 levels. This modulation correlates with significant improvements in neurological function and reduced neuronal and intestinal damage in both brain and colon tissues following CIRI [52]. EA's influence on the gut microbiota composition itself has also been observed; in middle cerebral artery occlusion (MCAO) mice models, EA treatment mitigated IS potentially by altering gut bacterial populations and affecting levels of metabolites like indole‐3‐propionic acid (IPA) [53]. Overall, EA exerts its anti‐inflammatory effects via the gut‐brain axis. This is achieved, in part, by enhancing the proliferation and activity of Tregs in both the ischemic brain and the small intestine, while concurrently reducing the levels of γδ T cells. This specific immunomodulatory effect inhibits the migration and infiltration of intestinal T cells into the brain, leading to a reduction in the proportion of cluster of differentiation 3‐positive T‐cell receptor gamma delta‐positive carboxyfluorescein succinimidyl ester‐positive (CD3^+^TCRγδ^+^CFSE^+^) cells within the cerebral tissue. Through these mechanisms, EA effectively attenuates neuroinflammation and supports the recovery process following ischemic injury [54, 55].

#### 2.2.2 Reduction of Excitatory Amino Acid (EAA)‐Induced Toxicity

**FIGURE 4:**
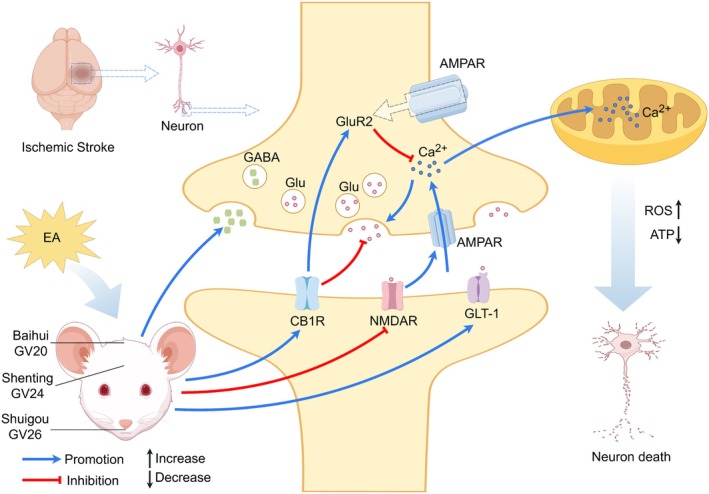
EA attenuates EAA‐induced neurotoxicity in IS. EA protects against excitotoxicity by maintaining glutamate/GABA balance and reducing NMDA receptor overactivation.

EAA‐induced neurotoxicity, primarily mediated by excessive glutamate release and subsequent overactivation of glutamate receptors, represents a major contributor to neuronal damage in IS. EA has been shown to effectively attenuate this detrimental process (Figure 4). A key mechanism involves the modulation of glutamate receptor function and extracellular glutamate levels. For instance, stimulation at specific acupoints such as GV20 and DU26 has been found to mitigate ischemic reperfusion injury in rats. This protective effect is associated with alterations in the expression of the N‐methyl‐D‐aspartate receptor (NMDAR) in the hippocampus and a reduction in intracellular calcium influx (Ca^2+^) [56, 57]. Such modulation directly impacts the downstream signaling cascades triggered by glutamate binding, thereby reducing excitotoxicity. Furthermore, EA can influence the concentration of excitatory neurotransmitters in the ischemic region. Studies have demonstrated that EA pretreatment at GV14 and GV20 leads to increased expression of glutamate transporter‐1 (GLT‐1) in the striatum of rats after MCAO followed by reperfusion. GLT‐1 is crucial for clearing extracellular glutamate from the synaptic cleft, and its upregulation by EA helps decrease the local glutamate (Glu) concentration, thus mitigating its neurotoxic effects [60].

In counterpoint to excitatory signaling, gamma‐aminobutyric acid (GABA)‐mediated inhibition plays a vital role in maintaining neuronal homeostasis. Evidence indicates that EA stimulation effectively diminishes ischemia‐induced excessive glutamate release while concurrently preserving or even enhancing the intrinsic inhibitory activity of GABA [61, 62]. This rebalancing of excitatory and inhibitory neurotransmission is a significant factor in the attenuation of excitotoxicity observed with EA. Additionally, research has explored the impact of EA on other factors influenced by ischemia. For example, EA has been shown to effectively mitigate the initial decrease in serum nitric oxide (NO) and the increase in plasma endothelin (ET) and brain calcium (Ca^2+^) levels caused by cerebral ischemia–reperfusion in rats [58]. While the direct link of NO and ET to EAA toxicity is complex, the reduction in brain Ca^2+^ influx directly ties into the mechanisms of excitotoxicity. Moreover, administering EA pretreatment prior to global cerebral ischemia has been reported to enhance glutamate receptor 2 (GluR2) expression in brain tissue through the activation of cannabinoid receptor 1 (CB1R). GluR2 is a subunit of AMPA receptors, another class of ionotropic glutamate receptors. This intervention enhanced neurological outcomes by promoting cell survival and inhibiting neuronal apoptosis [59], suggesting broader neuroprotective effects potentially related to modulating glutamate receptor signaling beyond NMDARs. Collectively, these findings highlight EA's multifaceted approach to reducing EAA‐induced toxicity through regulating neurotransmitter levels, receptor function, and ion homeostasis.

#### 2.2.3 Inhibition of Oxidative Stress

**FIGURE 5:**
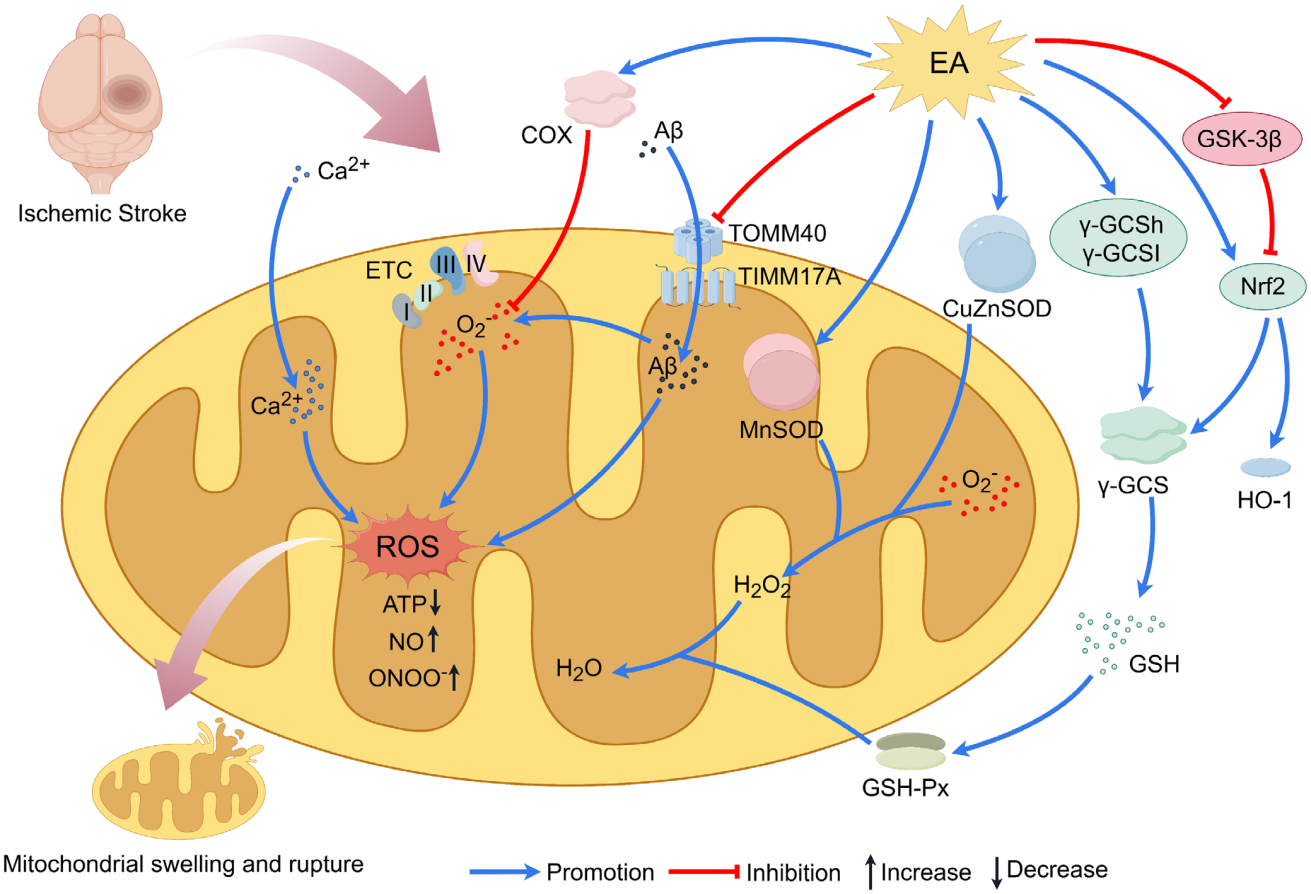
EA attenuation of oxidative stress in IS. EA activates endogenous antioxidant enzymes to scavenge ROS, enhances mitochondrial function, and upregulates the GSK‐3β/Nrf2/HO‐1 signaling pathway to mitigate oxidative stress and inflammation.

Oxidative stress is a critical contributor to neuronal injury following IS, resulting from an imbalance between the production of reactive oxygen species (ROS) and the capacity of endogenous antioxidant defense systems. EA has been extensively studied for its ability to attenuate oxidative stress in IS (Figure 5). A primary mechanism involves the activation and enhancement of the endogenous antioxidant enzyme system. This activation leads to a significant reduction in excessive ROS production, suggesting EA's potential in mitigating oxidative damage caused by cerebral ischemia and thereby contributing to its overall neuroprotective effects [64]. Studies using acupuncture and EA have consistently demonstrated the enhancement of activity of essential antioxidant enzymes, including superoxide dismutase (SOD), copper‐zinc superoxide dismutase (CuZnSOD), and manganese superoxide dismutase (MnSOD) [26, 27]. Furthermore, acupuncture and EA treatment can encourage the production of components related to cellular energy metabolism and antioxidant status, such as adenosine triphosphate (ATP), cyclooxygenase (COX), and reduced glutathione (GSH) [26]. For instance, EA stimulation at GV20 and GV14 has been shown to enhance the expression of γ‐glutamylcysteine synthetase (γ‐GCS) protein, as well as its heavy (γ‐GCSh) and light (γ‐GCSI) subunit mRNAs in the cortical parietotemporal regions of rats after cerebral ischemia–reperfusion. γ‐GCS is the rate‐limiting enzyme in GSH synthesis, and its upregulation by EA helps protect cortical cells from damage by facilitating the elimination of excess oxygen free radicals. Acupuncture treatment has also been reported to increase glutathione peroxidase (GSH‐Px) activity in the hippocampus, further bolstering the antioxidant defenses [27].

Specific signaling pathways are also modulated by EA to combat oxidative stress. The Nuclear factor erythroid 2‐related factor 2 (Nrf2)/Heme oxygenase 1 (HO‐1) pathway is a major regulator of antioxidant responses [93]. Acupuncture has been shown to enhance this pathway, elevating HO‐1 levels and consequently fostering both anti‐inflammatory and antioxidant effects [91]. Similarly, EA can mitigate oxidative stress induced by cerebral ischemia–reperfusion injury (CIRI) through the activation of the Glycogen synthase kinase 3 beta (GSK‐3β)/Nrf2 signaling pathway, operating in a Keap1‐independent manner [65]. While research highlights the interplay between NRF2 and NF‐κB pathways in regulating antioxidant and inflammatory responses in stroke [201], evidence strongly supports EA's positive impact on Nrf2‐mediated antioxidant defenses.

Beyond enzymatic defenses, acupuncture has also been implicated in protecting mitochondrial function, which is crucial for managing oxidative stress. It has been shown to inhibit the synthesis of mitochondrial outer membrane translocase 40 (TOM40) and mitochondrial inner membrane translocase 17A (TIM17A) and prevent the accumulation of β‐amyloid (Aβ) within the mitochondria [63]. Protecting mitochondrial integrity and function is vital as mitochondria are both major producers of ROS and targets of oxidative damage. These diverse mechanisms, from enhancing antioxidant enzymes and activating key signaling pathways to preserving mitochondrial health, collectively underscore EA's significant potential in inhibiting oxidative stress following IS.

#### 2.2.4 Modulation of Cellular Apoptosis and Pyroptosis

**FIGURE 6:**
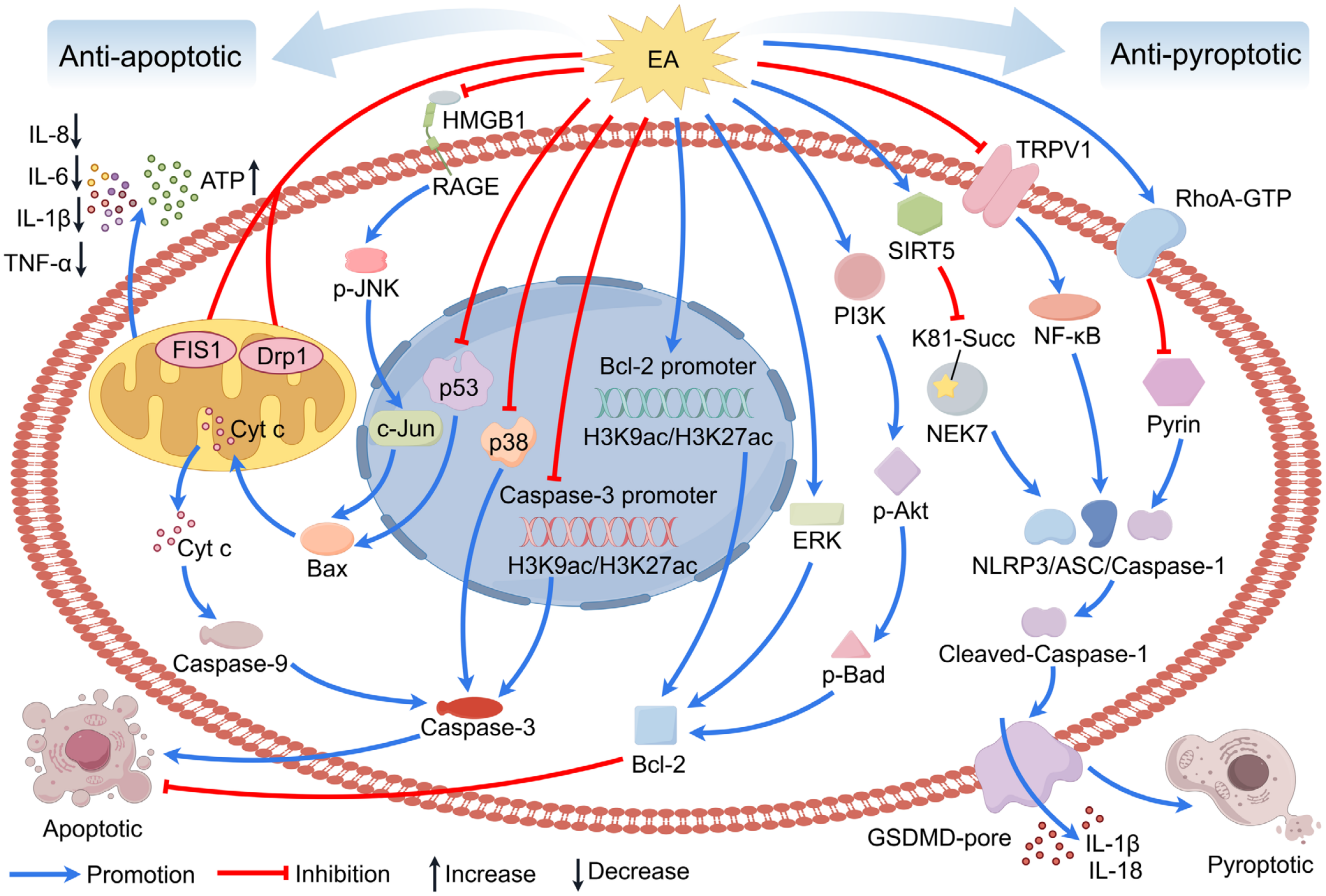
EA attenuates cellular apoptosis and pyroptosis in IS. EA concurrently suppresses apoptotic and pyroptotic cell death pathways, significantly reducing the release of pro‐inflammatory cytokines.

Beyond mitigating inflammation and excitotoxicity, EA also exerts neuroprotective effects by directly modulating programmed cell death pathways, particularly apoptosis and pyroptosis (Figure 6).

##### 2.2.4.1 Apoptosis

Apoptosis, or programmed cell death, is a significant process contributing to delayed neuronal loss and tissue damage following IS. EA has been demonstrated to effectively attenuate this cellular apoptosis through influencing various signaling pathways and key regulatory molecules. Stimulation at specific acupoints like Zusanli (ST36) and Quchi (LI11) has been shown to reduce neuronal apoptosis. This effect is mediated, in part, by activating signaling pathways such as the PI3K/Akt and Extracellular signal‐regulated kinase (ERK)/c‐Jun N‐terminal kinase (JNK)/p38 pathways [66, 67]. Activation of these pathways leads to increased expression of anti‐apoptotic proteins, including Phosphorylated protein kinase B (p‐Akt), Phosphorylated B‐cell lymphoma 2 (Bcl‐2)‐associated death promoter (p‐Bad), and Bcl‐2, while simultaneously decreasing the levels of proapoptotic proteins such as Bcl‐2‐associated death promoter (Bad) and Bcl‐2‐associated X protein (Bax) [66, 67]. This results in an elevated Bcl‐2/Bax ratio, a critical determinant of cell fate, thereby suppressing the activation of effector caspases like Cysteine‐aspartic protease 3 (Caspase‐3) [66, 67]. The ultimate outcome is reduced neuronal apoptosis, a decrease in infarct volume, and enhanced neurological function in experimental stroke models [66, 67].

Further studies reinforce EA's impact on the Bcl‐2 family and caspases. EA stimulation at ST36 and LI11 specifically enhances the Bcl‐2/Bax ratio in ischemic brain tissue by modulating the gene expression of Bcl‐2 and Bax [68, 69]. Similarly, EA applied at GV20 and Shenting (GV24) has been shown to mitigate cell apoptosis in cerebral ischemia by upregulating the anti‐apoptotic protein Bcl‐2 and downregulating proapoptotic factors such as Bax, Caspase‐3, and Cysteine‐aspartic protease 9 (Caspase‐9) [70]. EA pretreatment may also protect against neuronal apoptosis by downregulating the expression of the tumor suppressor protein p53 and Caspase‐3, thereby mitigating ischemic injury in rat brain tissue [71]. Beyond protein expression, EA can influence apoptosis at the epigenetic level. EA enhances neuroprotection in ischemia–reperfusion injury by modulating histone acetylation at key gene promoters. Specifically, EA increases H3K9 acetylation (H3K9ace) and H3K27 acetylation (H3K27ace) at the *Bcl‐2* promoter, leading to elevated mRNA and protein expression of Bcl‐2 in ischemic brain tissue. Concurrently, it reduces H3K9ace and H3K27ace enrichment at the *caspase‐3* promoter, thereby downregulating the protein expression of Bax and cleaved Caspase‐3 [73]. EA preconditioning at GV20, bilateral Shenshu (BL23), and SP6 has been shown to confer neuroprotection against CIRI in rats with MCAO by modulating apoptotic signaling pathways. This protective effect is notably linked to the inhibition of the NF‐κB signaling pathway, which leads to reduced transcriptional activity of NF‐κB (p65). Consequently, EA preconditioning downregulated the expression of the proapoptotic proteins Bax and cleaved Caspase‐3 while enhancing the expression of the anti‐apoptotic protein Bcl‐2. Additionally, it suppressed the expression of transient receptor potential vanilloid 1 (TRPV1), suggesting that EA may protect neuronal integrity through the coordinated regulation of apoptosis‐related proteins and potentially related inflammatory pathways [74]. Furthermore, EA has been found to reduce the expression levels of mitochondrial fission‐related proteins (Fis1) and dynamin‐related protein 1 (Drp1), which are implicated in mitochondrial dysfunction and apoptosis. This reduction correlates with improved neuronal mitochondrial damage and reduced neurocyte apoptosis in MCAO mice, as well as alleviated motor dysfunction and reduced infarct volume [84]. Acupuncture, generally, shows potential in reducing cell apoptosis, a benefit that is also associated with its ability to enhance the expression of neurotrophic factors like glial cell‐derived neurotrophic factor (GDNF) and brain‐derived neurotrophic factor (BDNF) [72], which support neuronal survival.

##### 2.2.4.2 Pyroptosis

Distinct from apoptosis, pyroptosis is a highly inflammatory form of programmed cell death, often triggered by inflammasome activation, particularly in microglia and other immune cells. EA exerts significant neuroprotective effects by inhibiting microglial pyroptosis following brain ischemia–reperfusion injury. This inhibition occurs, in part, through the suppression of the Ras homolog family member A (RhoA)/Pyrin/Gasdermin D (GSDMD) signaling pathway [75]. Modulating this pathway helps to alleviate the overall brain damage caused by ischemia–reperfusion, contributing to reduced inflammation and preserved neural integrity [75]. EA has been shown to significantly downregulate the expression of key components of the NLRP3 inflammasome pathway, including Apoptosis‐associated speck‐like protein containing a CARD (ASC), Cysteine‐aspartic protease 1 (Caspase‐1), and GSDMD, as well as the downstream inflammatory cytokine IL‐1β. This demonstrates that EA can effectively regulate the NOD‐like receptor family pyrin domain containing 3 (NLRP3)/ASC/Caspase‐1‐mediated pyroptosis pathway [76]. This regulation is crucial for the alleviation of neuronal damage observed during cerebral ischemia–reperfusion [76]. Moreover, EA can influence other pathways involved in regulating pyroptosis. For example, EA has been shown to enhance the expression of Sirtuin 5 (SIRT5) and inhibit the succinylation modification of NIMA‐related kinase 7 (NEK7) at the lysine 81 (K81) site [92]. This modulation suppresses both brain ischemia–reperfusion injury and neuronal pyroptosis [92], highlighting another specific molecular target for EA's anti‐pyroptotic effects.

EA exerts dual‐strategic advantages in ischemic brain injury by concurrently inhibiting apoptosis and pyroptosis, thereby reducing overall cell loss and mitigating secondary damage. Notably, although apoptosis and pyroptosis represent two distinct forms of programmed cell death with divergent characteristics, emerging evidence suggests a complex interplay between these pathways in the ischemic brain. Whether this interaction constitutes a key mechanism underlying the neuroprotective effects of EA represents an important direction for future research. In‐depth investigation into the regulatory effects of EA on the apoptosis‐pyroptosis crosstalk will contribute to a more comprehensive elucidation of the molecular basis for its therapeutic efficacy in IS.

#### 2.2.5 Regulation of Autophagy

**FIGURE 7:**
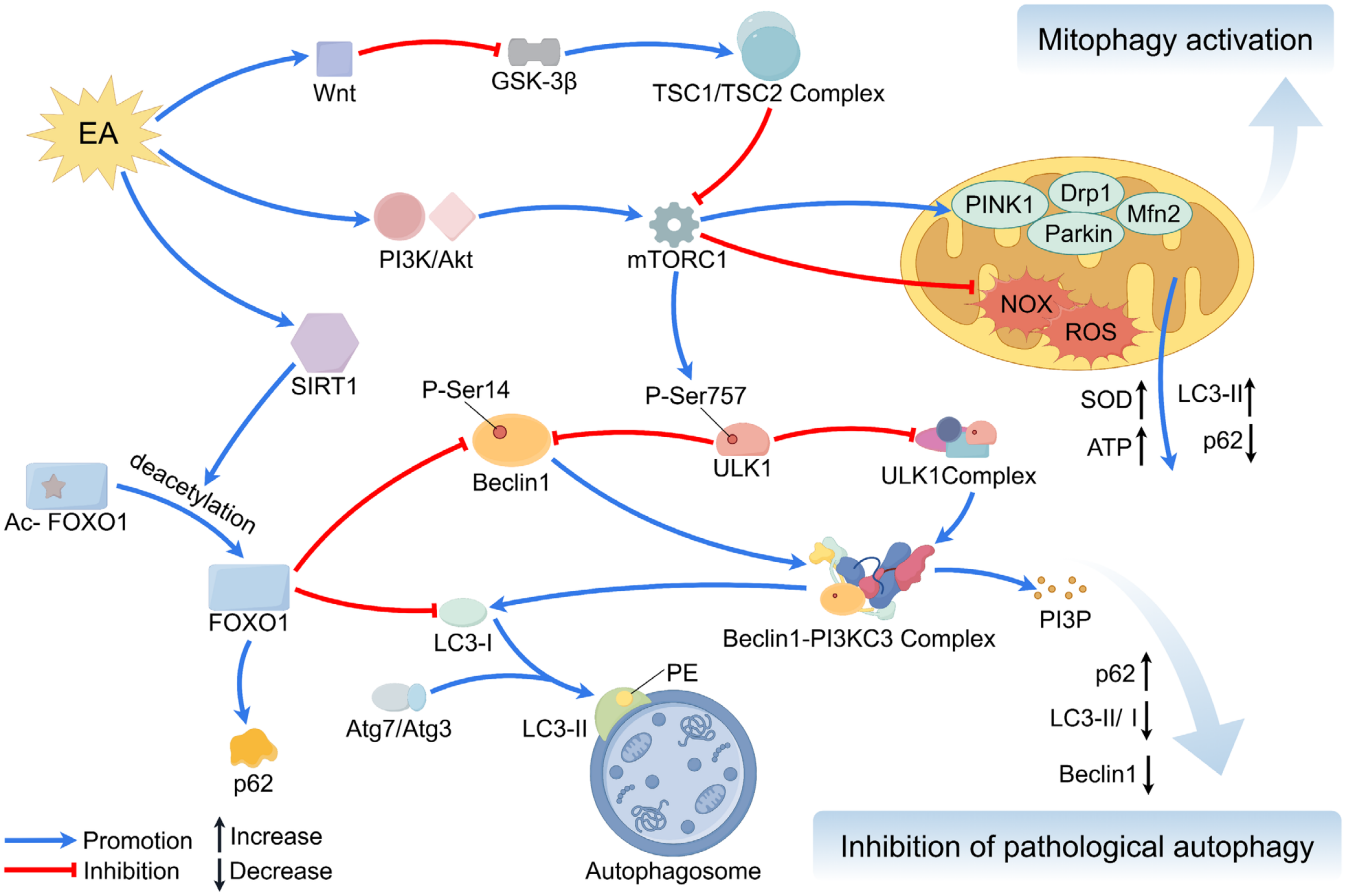
EA modulates autophagy in IS. EA suppresses pathological autophagy while enhancing mitophagy to eliminate damaged mitochondria.

Autophagy, a fundamental cellular process involving the degradation and recycling of damaged organelles and protein aggregates, plays a complex and context‐dependent role following IS. While excessive autophagy can be detrimental during the acute phase, timely and appropriate autophagy, particularly mitochondrial autophagy (mitophagy), is crucial for clearing damaged components and promoting recovery during reperfusion. EA has been shown to modulate autophagy in IS (Figure 7), although the direction of this modulation appears to vary across studies, potentially depending on the intervention timing, acupoint selection, and specific stroke model.

Some studies suggest that EA primarily acts by inhibiting excessive autophagy, particularly during the initial phase of ischemia–reperfusion. Following EA pretreatment at GV20, researchers observed decreased levels of canonical autophagy marker proteins, such as Microtubule‐associated protein 1 light chain 3‐II (LC3‐II) and Beclin‐1, 12 h after cerebral ischemia/reperfusion (I/R) in rats. EA reduced autophagy marker expression and autophagosome numbers in cortical tissue, thus protecting the brain from ischemia/reperfusion injury by inhibiting autophagy [78]. Similarly, EA has been proposed to reduce neuronal damage during cerebral ischemia–reperfusion by lowering the expression of autophagy‐related proteins like Beclin1 and LC3, thereby mitigating what is considered excessive autophagy in neurons during reperfusion [79]. EA pretreatment at GV20, LI11, and ST36 has also been reported to offer neuroprotection against CIRI by activating the Sirtuin 1 (SIRT1)/Forkhead box O1 (FOXO1) signaling pathway, leading to the suppression of autophagy [80]. Further evidence supporting autophagy inhibition comes from studies showing that EA at LI11 and ST36 decreases the number of autophagosomes, autolysosomes, and lysosomes. This effect correlates with reduced expression of autophagosome membrane markers, including microtubule‐associated protein 1 light chain 3 beta (LC3β) II/I and Beclin1, while the expression of Mammalian target of rapamycin complex 1 (mTORC1), a key negative regulator of autophagy initiation, is elevated. These findings suggest that EA stimulation may prevent IS by inhibiting autophagosome formation and autophagy via the mTORC1‐ULK complex‐Beclin1 pathway [81]. Additionally, EA preconditioning has been found to mitigate ischemic brain injury predominantly through activation of the Wnt/GSK3β signaling pathway within the ischemic penumbra, thereby leading to a reduction in infarct volume, attenuation of neurological deficits, and an associated inhibition of autophagy [86].

However, other research indicates that EA may instead promote or enhance specific aspects of autophagy, particularly those related to clearance and repair. EA treatment during the acute phase of IS has been shown to regulate autophagy markers in a manner suggesting modulated flux rather than simple inhibition. This includes increasing the expression of SIRT1, Phosphorylated extracellular signal‐regulated kinase 1/2 (p‐ERK1/2), Phosphorylated c‐Jun N‐terminal kinase (p‐JNK), Sequestosome 1 (P62), and Lysosomal‐associated membrane protein 1 (LAMP1), while reducing the expression of Cleaved caspase‐3 (CCAS3), LC3II/I, and Beclin1 [85]. The increase in P62 and LAMP1, markers associated with autophagosome cargo recruitment and lysosomal fusion respectively, alongside decreased initiation markers (LC3, Beclin1), might suggest enhanced autophagic flux or a shift toward specific types of autophagy. Furthermore, a study on elderly rats showed that EA pretreatment mitigated oxidative stress and *boosted* autophagy via the SIRT1/FOXO1 pathway, leading to enhanced postoperative cognitive function [77]. This finding appears to contradict the inhibitory effect linked to SIRT1/FOXO1 in [80], suggesting context‐dependent or complex pathway modulation.

More specifically, EA appears to positively regulate mitophagy, the selective autophagy of damaged mitochondria. EA alleviates mitochondrial dysfunction caused by nitrosative/oxidative stress by promoting Pink1/Parkin‐mediated mitochondrial autophagy. This selective clearance reduces the accumulation of damaged mitochondria, which are major sources of ROS and drivers of cell death. This process enhances mitochondrial membrane potential (MMP) and ATP levels, helps correct alkaline phosphatase (ALP) dysfunction, and improves mitochondrial autophagic clearance, thereby robustly protecting neurons from I/R‐induced damage [87]. Acupuncture at GV20, DU26, and GV14 has also been proposed to enhance hippocampal neuronal autophagy and prevent apoptosis by modulating the type III PI3K/Beclin‐1 pathway and increasing miR‐34c‐5p expression [82, 83]. Given the context of neuronal protection and mitochondrial health, this “enhancement” may specifically relate to beneficial forms of autophagy like mitophagy.

##### 2.2.5.1 In‐Depth Analysis of the Divergent Effects of Electroacupuncture on Autophagy

These seemingly contradictory findings in fact highlight the precision and complexity of EA in modulating autophagy. The observed discrepancies may arise from several key factors.

###### 2.2.5.1.1 Intervention Timing and Disease Stage Dependence

This is arguably the most critical determinant of inconsistent outcomes across studies. Autophagy plays distinct—and sometimes opposing—roles at different phases of IS. During ischemia and the early reperfusion period, energy depletion and oxidative stress can trigger excessive, nonselective autophagy, potentially culminating in autophagic cell death; under such conditions, autophagy inhibition may be protective [78, 79, 80, 81, 86]. By contrast, during the later reperfusion phase or the recovery period, a moderate increase in autophagy—particularly mitophagy targeting damaged mitochondria—facilitates the clearance of toxic intracellular debris, provides metabolic substrates, and promotes neuronal remodeling and functional recovery [82, 83, 87]. This time‐dependent duality resembles a “U‐shaped” dose–response relationship, in which both insufficient and excessive autophagy are detrimental, whereas an intermediate “optimal window” is most conducive to cell survival. EA may function by dynamically adjusting autophagy toward this optimum: suppressing autophagy when it is excessive (e.g., the acute ischemic phase), while enhancing autophagy when it is insufficient or when specific forms of autophagy are required for cellular cleanup and repair (e.g., the recovery phase). For example, EA pretreatment (as a prophylactic‐like paradigm) may shift basal autophagy to a more appropriate set point before ischemia occurs, thereby manifesting as an inhibitory effect in acute injury models [78, 80, 86]. In contrast, interventions initiated 24 h after reperfusion may coincide with a greater demand for reparative autophagy, resulting in changes in markers consistent with enhanced autophagic flux [85].

###### 2.2.5.1.2 Acupoint Specificity

Different acupoint combinations may engage distinct neural circuits and downstream signaling cascades. For instance, protocols using only limb acupoints (e.g., LI11 and ST36) primarily activate somatosensory pathways and have been more frequently associated with autophagy suppression [81]. Conversely, prescriptions including cranial points along the Governor Vessel (e.g., GV20, GV26, and GV14) may more directly influence the central nervous system and have been reported to promote autophagy [82, 83]. These observations suggest that differences in acupoint selection may critically determine which signaling pathways are recruited and, consequently, the direction of autophagy modulation. Future studies should systematically compare the acupoint‐specific regulation of autophagy and its upstream pathways.

###### 2.2.5.1.3 Limitations of Autophagy Assessment Methods and Markers

Most studies rely primarily on static markers of autophagosome formation, such as LC3‐II and Beclin1, which reflect autophagosome abundance but do not directly indicate autophagic flux (i.e., completion of the degradative process) [78, 79, 80, 81, 83, 86]. Increased autophagosome numbers can result either from enhanced autophagy induction or from impaired autophagosome–lysosome degradation. In one study, decreases in LC3‐II/I and Beclin1 were accompanied by increases in p62 and LAMP1 [85]. Although p62 accumulation typically suggests impaired flux, p62 can also increase due to transcriptional upregulation even when lysosomal function is enhanced. When interpreted together with elevated LAMP1 (a lysosomal marker), these results more likely reflect a dynamic change in autophagic flux: reduced autophagosome formation rate combined with increased lysosome abundance and/or activity, such that overall clearance efficiency (flux) is maintained or even improved. Therefore, conclusions based solely on static markers may be incomplete or misleading. Future work should incorporate flux assays (e.g., LC3‐II turnover assays, tandem fluorescent LC3 reporters) to more accurately characterize the effects of EA on autophagy.

###### 2.2.5.1.4 Differences in Stimulation Parameters and Experimental Models

Even among studies using EA pretreatment and similar frequencies (2/15 Hz) [78, 80, 86], subtle differences remain in stimulation intensity (e.g., 1 mA), treatment duration, and course. More importantly, heterogeneity in animal models (e.g., the four‐vessel occlusion global cerebral ischemia model [79] versus the more commonly used MCAO focal ischemia model) and in sampling time points (ranging from 12 h to several days after reperfusion) directly affects baseline injury severity and autophagy status, complicating cross‐study comparisons. For example, when EA was initiated as early as 1 h after reperfusion and repeated every 12 h until 48 h [79], the predominant observation was autophagy inhibition, consistent with the hypothesis that early intervention mainly suppresses excessive autophagy. In contrast, when a single EA session was delivered starting 24 h after reperfusion [85], the effects appeared more consistent with modulation of autophagic flux, further underscoring the importance of intervention timing.

In summary, EA regulation of autophagy after stroke is unlikely to be a simple binary “on/off” phenomenon. Rather, it represents a finely tuned, dynamic process determined by pathological stage, acupoint prescription, stimulation parameters, and autophagy subtype. Future investigations should move beyond the dichotomy of “inhibition versus activation” and instead address the following questions: How does EA dynamically regulate autophagic flux across different poststroke phases? How can parameters (timing, acupoints, frequency, intensity) be optimized to precisely target specific forms of autophagy (e.g., mitophagy)? Can more advanced techniques enable in vivo and longitudinal monitoring of autophagic flux after EA? Addressing these issues will be essential for elucidating the complex mechanisms of EA and for providing a stronger theoretical foundation for clinical translation.

#### 2.2.6 Inhibition of Ferroptosis

**FIGURE 8:**
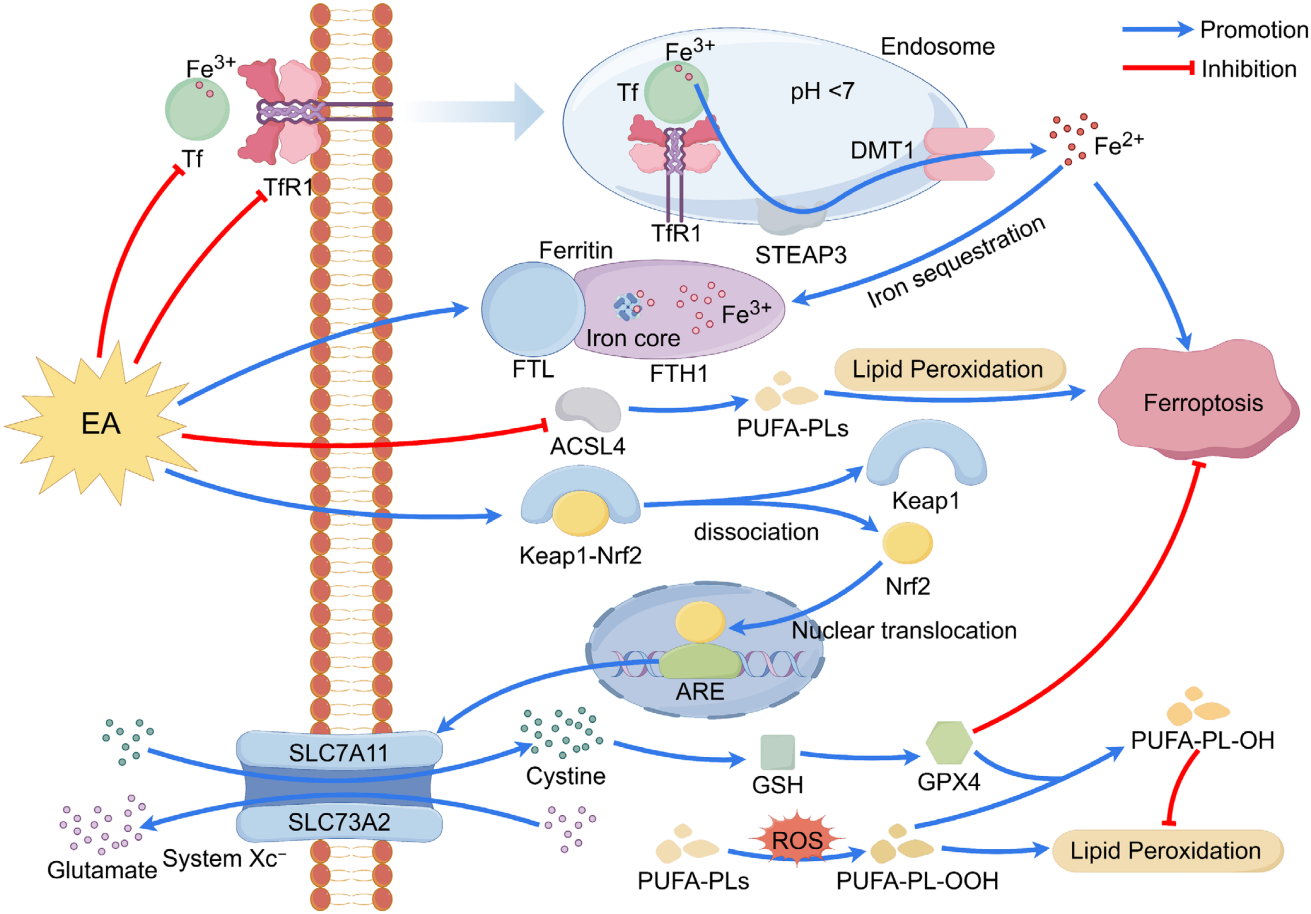
EA inhibits ferroptosis in IS. EA treatment enhances Nrf2‐mediated GPX4 transcription, downregulates iron import, and increases iron storage, collectively preventing ferroptosis.

Ferroptosis is a recently recognized form of regulated cell death characterized by iron‐dependent lipid peroxidation. It plays a significant role in neuronal injury following IS, distinct from apoptosis and necroptosis. EA has emerged as a promising intervention to inhibit ferroptosis in the context of IS (Figure 8). EA can mitigate IS‐induced brain injury not only by targeting apoptosis, necroptosis, and autophagy, but also specifically by suppressing ferroptosis.

Multiple studies point toward the Nrf2/Solute carrier family 7‐member 11 (SLC7A11)/glutathione peroxidase 4 (GPX4) pathway as a key mediator of EA's anti‐ferroptotic effects. EA treatment promotes the nuclear translocation of Nrf2, a master regulator of antioxidant responses. Activating Nrf2 enhances the expression of its downstream targets, including SLC7A11 and GPX4 [89, 90]. SLC7A11 is a component of the cystine/glutamate antiporter system (xc−), crucial for importing cystine needed for glutathione synthesis. GPX4 is a pivotal enzyme that detoxifies lipid peroxides, preventing the lipid peroxidation that is the hallmark of ferroptosis. By upregulating SLC7A11 and GPX4, EA bolsters the cell's capacity to synthesize glutathione and detoxify lipid peroxides, thereby directly inhibiting ferroptosis [89, 90]. EA treatment has also been shown to upregulate ferritin heavy chain 1 (FTH1) expression [90, 94]. FTH1 is involved in iron storage, and its increase can reduce the pool of free intracellular iron available for lipid peroxidation, further contributing to ferroptosis inhibition [90, 94]. EA preconditioning has been demonstrated to activate Nrf2 and reduce the expression of its negative regulator, Kelch‐like ECH‐associated protein 1 (Keap1), to inhibit ferroptosis and delay the progression of IS [93].

Beyond specific pathways, EA's anti‐ferroptotic effects are also linked to its known ability to reduce oxidative stress and modulate iron metabolism [94]. EA intervention in ischemic brain tissue decreases levels of malondialdehyde (MDA), a marker of lipid peroxidation, and total iron. Concurrently, it increases the activity of antioxidant enzymes, including SOD and GSH, which combat general oxidative stress. More specifically related to ferroptosis, EA upregulates the expression of GPX4 and FTH1, key regulators of iron metabolism and defense against lipid peroxidation, while downregulating transferrin (Tf), transferrin receptor 1 (TfR1), and acyl‐CoA synthetase long‐chain family member 4 (ACSL4) [88], thereby reducing intracellular iron accumulation and subsequent lipid peroxidation. Furthermore, EA promotes the restoration of mitochondrial morphology [89, 94], suggesting that preserving mitochondrial integrity, which is severely compromised during ferroptosis, may also contribute to its protective effects. These cumulative findings strongly indicate that EA exerts neuroprotective effects in ischemic brain injury by comprehensively modulating oxidative stress and iron‐associated proteins, thus effectively suppressing ferroptosis [94].

#### 2.2.7 Preservation of Blood–Brain Barrier Integrity

**FIGURE 9:**
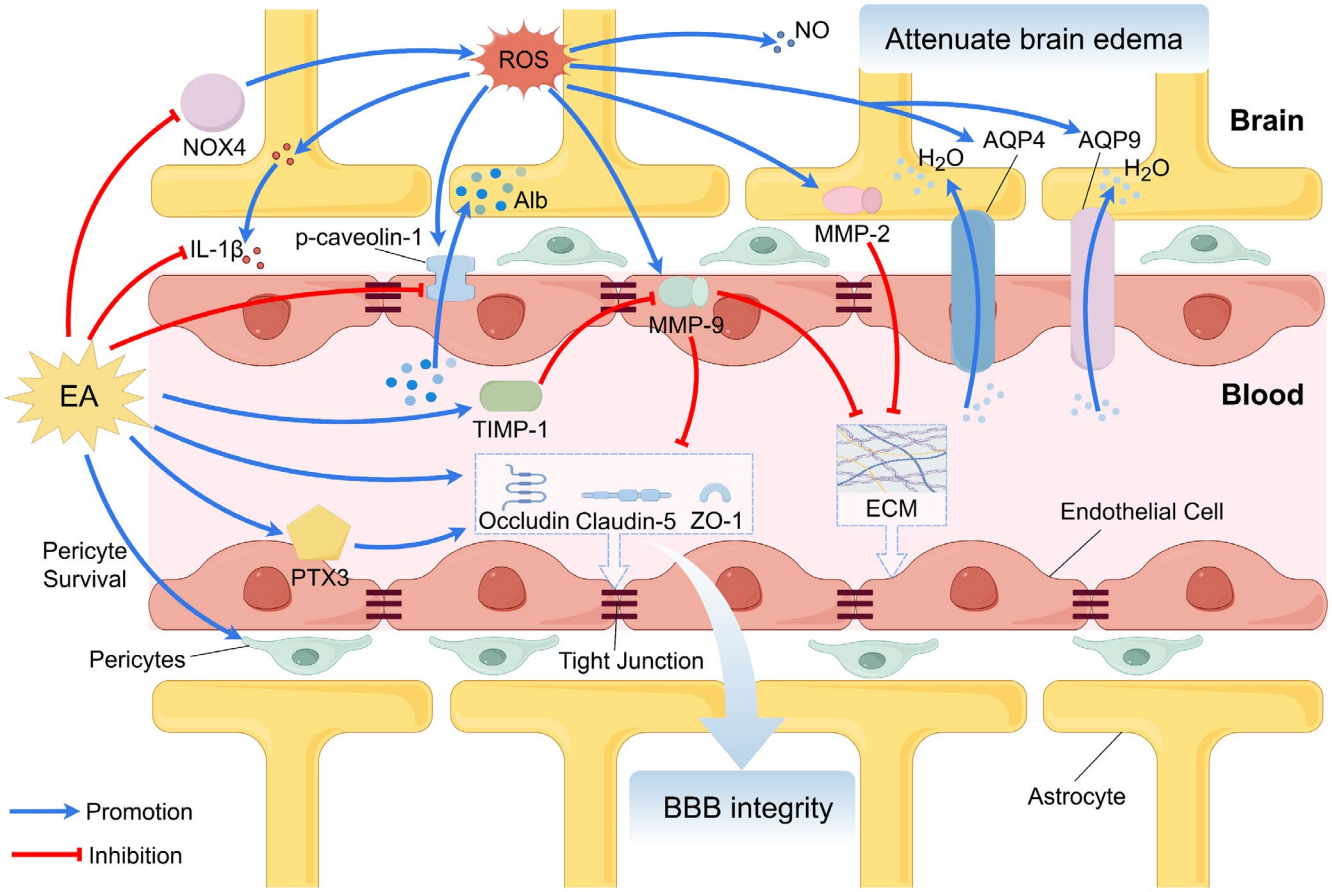
EA preserves BBB integrity in IS. EA alleviates BBB disruption and cerebral edema by upregulating tight junction proteins (e.g., claudin‐5, occludin, ZO‐1) while suppressing AQP4, AQP9, MMP‐2, and MMP‐9 expression.

The blood–brain barrier (BBB) is a critical structure that maintains central nervous system homeostasis by regulating the passage of substances from the bloodstream into the brain. Integrity of the BBB is severely compromised following IS, leading to increased permeability, vasogenic edema, and infiltration of peripheral inflammatory cells, all of which exacerbate brain injury. Importantly, clinical and experimental evidence demonstrates that poststroke hyperglycemia significantly potentiates BBB disruption while elevating the risk of hemorrhagic transformation [202]. EA plays a vital role in preserving BBB integrity in IS (Figure 9).

Existing studies suggest that acupuncture and EA interventions can significantly improve BBB function during IS. During acute ischemia, EA may mitigate BBB hyperpermeability and reduce brain edema. This protective effect is often associated with enhancing the expression of tight junction proteins, such as Zonula occludens‐1 (ZO‐1) and claudin‐5, which are crucial for maintaining the seal between endothelial cells [95, 96]. Concurrently, EA can reduce levels of ROS and astrocytic aquaporin‐4 (AQP4) in the affected area, further contributing to reduced edema and improved barrier function [95, 96]. Scalp acupuncture, a specific form of acupuncture application, has been shown to enhance neurological function and mitigate BBB damage in rats with acute ischemic cerebrovascular disease. This effect may be linked to its capacity to enhance the expression of pentraxin 3 (PTX3) and increase ZO‐1 and occludin mRNA levels [97]. Occludin is another key tight junction protein, and preserving its expression at the mRNA level supports barrier integrity.

Another mechanism by which EA preserves BBB integrity is by modulating the activity of matrix metalloproteinases (MMPs), enzymes that degrade the extracellular matrix and tight junction proteins. EA inhibits the expression and activity of MMP‐2 and MMP‐9, which are strongly linked to BBB permeability and disruption after stroke [98]. EA stimulation at GV20 specifically may modulate MMP‐9 and Tissue inhibitor of metalloproteinase‐2 (TIMP‐2) transcription via histone acetylation during the acute stroke phase, contributing to the preservation of BBB integrity in MCAO rats [99]. Conversely, EA enhances the expression of tissue inhibitors of metalloproteinases (TIMPs), such as TIMP‐1 [101], which are endogenous inhibitors of MMPs, further tipping the balance toward preserving BBB structure. Beyond tight junctions and MMPs, EA also inhibits the expression of aquaporins AQP4 and AQP9, which are involved in water transport and edema formation associated with BBB disruption [98].

Emerging research also highlights the protection of pericytes, cells essential for BBB structure and function, as a mechanism. EA preconditioning at GV26 and GV20 in rats subjected to cerebral ischemia–reperfusion yields EA serum that can reduce pericyte apoptosis and migration, enhance pericyte viability, and mitigate BBB damage induced by oxygen–glucose deprivation/reoxygenation (OGD/R) in vitro [100]. This suggests that EA may exert protective effects on the BBB through humoral factors. Furthermore, preserving BBB integrity is critical for extending the therapeutic window for thrombolytic treatment. EA's ability to inhibit MMP‐9 and enhance TIMP‐1 expression helps maintain BBB integrity, potentially allowing for later administration of thrombolytics [101]. Interestingly, EA has also been shown to reduce the expression of pyroptosis‐related proteins, such as GSDMD, IL‐1β, and interleukin‐18 (IL‐18) [101], potentially linking its anti‐pyroptotic effects (as discussed in Section 2.2.4.2) to reduced inflammatory damage to the BBB.

While most studies indicate that EA enhances tight junction proteins like ZO‐1 and Occludin, one study using specific mode electroacupuncture stimulation (SMES) reported preserving BBB permeability via activating p‐p65, which enhanced vascular endothelial growth factor A (VEGFA) expression while surprisingly *decreasing* the levels of tight junction proteins, such as Occludin and ZO‐1 [102]. This contradictory finding warrants further investigation to understand the specific conditions or mechanisms under which such a response might occur, but the preponderance of evidence supports EA's role in *enhancing* tight junction integrity. Overall, EA preserves BBB integrity through complex mechanisms involving modulation of tight junctions, MMPs, aquaporins, pericyte protection, and possibly influencing inflammatory cell death pathways.

### 2.3 Electroacupuncture Promotes Neurorepair in Ischemic Stroke

#### 2.3.1 Regulation of Cerebral Blood Flow

**FIGURE 10:**
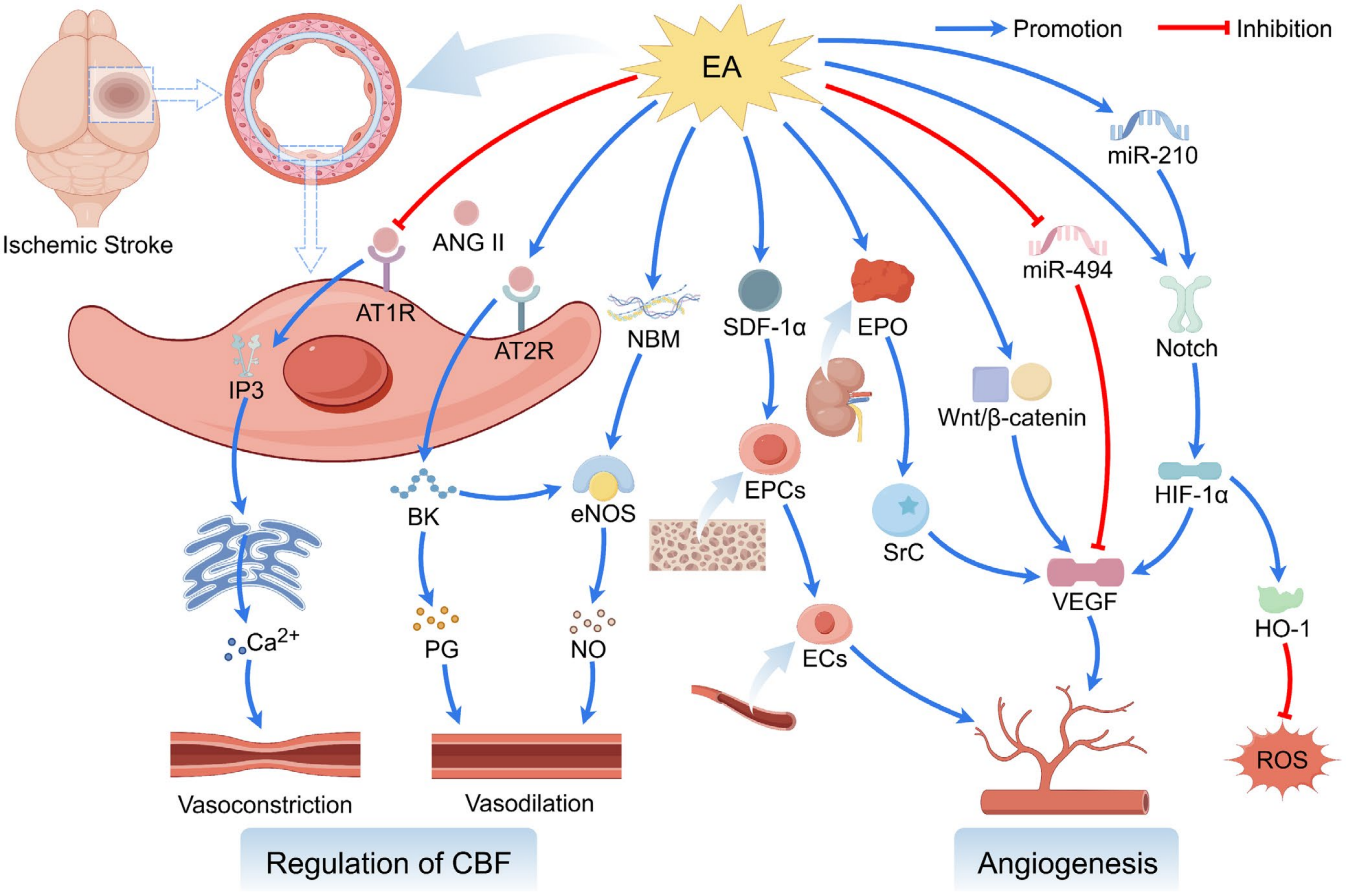
EA modulates CBF and promotes angiogenesis in IS. EA enhances cerebral hemodynamics by suppressing pathological vasoconstriction and promoting active vasodilation, while simultaneously stimulating vascular regeneration to improve cerebral blood volume.

Restoring adequate CBF to the ischemic penumbra is the primary goal of acute stroke management and is crucial for neuronal survival and functional recovery. IS results from the blockage of cerebral arteries, causing significant disruption of blood flow to brain tissue and leading to necrosis in the core and potential salvageable injury in the penumbra. EA plays an important role in CBF regulation and angiogenesis promotion in IS (Figure 10).

Studies utilizing acupuncture and EA have consistently demonstrated their capacity to enhance CBF and improve microcirculation in stroke models [103, 104, 203]. This improvement in blood flow can occur through various mechanisms. At the molecular level, EA potentially influences vasoactive pathways, such as the angiotensin II receptor‐mediated IP3 signaling pathway, by inhibiting its expression [105]. Modulation of such pathways can lead to vasodilation and improved blood flow. Additionally, EA may alter the expression of stroke‐related microRNAs (miRNAs) that are linked to cellular processes like proliferation and vascular tone, potentially decreasing vasoconstriction in ischemic regions and thereby enhancing CBF [106]. EA enhances hypoxia‐inducible factor (HIF)‐1α expression and decreases brain infarct size [107, 108]. EA combined with rehabilitation training may enhance angiogenesis‐related factor expression in ischemic brain tissue, decrease infarct volume, and improve local CBF in acute IS rats [109]. EA can enhance cerebral perfusion and increase the diameter of developed dural anastomoses by activating cholinergic neurons in the Meynert basal nucleus (NBM), thereby promoting collateral circulation in the dura mater [110].

#### 2.3.2 Promotion of Angiogenesis

Angiogenesis, the formation of new blood vessels from preexisting ones, is a crucial reparative process that occurs in the ischemic penumbra after stroke. It helps to restore blood flow, improve tissue perfusion, and deliver oxygen and nutrients to the compromised brain tissue, thereby promoting neuronal survival and functional recovery. EA has been widely demonstrated to promote angiogenesis in IS (Figure 10, also illustrating CBF regulation).

EA treatment in experimental stroke models consistently shows beneficial effects on the cerebrovascular system. For instance, EA treatment in MCAO rats increases endothelial cell (EC) proliferation within 12 h [103], a fundamental step in angiogenesis. EA intervention protects ischemic brain tissue by mitigating microvascular ultrastructure damage, enhancing the expression of vascular endothelial growth factor (VEGF) mRNA, and promoting capillary angiogenesis and the reconstruction of the microvascular network [111]. VEGF is a key angiogenic factor, and its upregulation is a common finding. EA enhances the expression of several other angiogenesis‐related factors, including basic fibroblast growth factor (bFGF), angiopoietin (Ang)‐1/2, and platelet‐derived growth factor b (PDGF‐b), all of which facilitate angiogenesis and support neural recovery [113]. EA stimulation at GV20 not only boosts regional CBF but also increases blood vessel density in the infarcted area. This enhanced vascularization is achieved, in part, by upregulating various angiogenic factors and the cell proliferation marker Ki67 [104].

The pro‐angiogenic effects of EA are mediated through the modulation of multiple signaling pathways. The HIF/VEGF/Notch signaling pathway is centrally involved in the response to hypoxia and subsequent angiogenesis. EA stimulation at GV20 and ST36 enhances angiogenesis in the ischemic penumbra and activates the HIF/VEGF/Notch signaling pathway, leading to improved neurological function and reduced infarct size in IS rats [114]. Similarly, EA at specific acupoints, including PC6, GV26, and SP6, can activate the VEGF/Notch signaling pathway. This activation results in the upregulation of key molecules such as VEGFA, Notch1, and its downstream target protein Hes1. This cascade promotes angiogenesis, enhancing the formation of new blood vessels, and also reverses EC death, contributing to improved tissue repair, reduced ischemic injury, and ultimately supporting functional recovery [20]. Furthermore, EA promotes angiogenesis under hypoxic conditions by activating the HIF‐1α/VEGF/Notch1 signaling pathway through exosome‐delivered microRNA‐210 (miR‐210) [118, 119]. This mechanism involves the transfer of pro‐angiogenic factors via exosomes, highlighting a potential paracrine effect of EA [118, 119].

Other pathways also contribute to EA‐induced angiogenesis. EA upregulates the expression of Irisin, a myokine, which activates the VEGF/Akt/eNOS signaling pathway, thereby promoting vascular remodeling following cerebral ischemia [115]. EA stimulation at GV26 may enhance the expression of miR‐142‐5p, which subsequently suppresses the expression of its target gene ADAMTS1. This process results in the upregulation of VEGF, activation of the PI3K/AKT signaling pathway, and the release of eNOS, all contributing to improved neurological injury, reduced infarct size, and promoted angiogenesis in rats with IS [116]. Scalp acupuncture has been shown to activate the Wnt/β‐catenin signaling pathway, thereby promoting the expression of various angiogenic factors and restoring blood perfusion in ischemic regions. This activation leads to a significant upregulation of the mRNA and protein levels of VEGF, fetal liver kinase 1 (FLK1), basic fibroblast growth factor (bFGF), and Ang2 in ischemic brain tissue [117]. These findings strongly suggest that the restoration of blood flow observed with scalp acupuncture is significantly attributed to enhanced angiogenesis [117]. EA stimulation at GV20 and GV26 also protects the neurovascular unit by modulating the canonical Wnt/β‐catenin signaling pathway, upregulating VEGF, glial fibrillary acidic protein (GFAP), neuronal nuclear antigen (NeuN), and β‐catenin while downregulating Axin2, leading to reduced infarct volume and improved neurological deficits [121].

Moreover, EA stimulation at GV26 has been shown to activate the erythropoietin (EPO)‐mediated signaling pathways, specifically involving Src (proto‐oncogene tyrosine‐protein kinase) and VEGF. This activation promotes angiogenesis, which is crucial for tissue repair, and alleviates ischemic damage by enhancing blood vessel formation. The upregulation of VEGF appears to be closely linked to the activation of Src kinase pathways by EPO, suggesting a key role for EPO in orchestrating the neurovascular recovery process following ischemic injury [120]. EA also enhances stromal cell‐derived factor‐1 (SDF‐1) production, promoting the mobilization of endothelial progenitor cells (EPCs) [112]. EPCs are circulating cells that can differentiate into endothelial cells and contribute to neovascularization, providing another mechanism by which EA supports vascular repair.

In conclusion, EA promotes angiogenesis after IS through a diverse array of mechanisms involving the upregulation of multiple growth factors (VEGF, bFGF, Ang, PDGF, SDF‐1, EPO), activation of key signaling pathways (HIF/VEGF/Notch, Wnt/β‐catenin, PI3K/Akt/eNOS, EPO/Src), modulation of microRNAs (miR‐142‐5p, miR‐210), mobilization of EPCs, and protection of the neurovascular unit. These multifaceted effects contribute significantly to vascular repair, improved blood supply to the ischemic tissue, and ultimately, better functional outcomes.

#### 2.3.3 Promotion of Neural Regeneration and Repair

**FIGURE 11:**
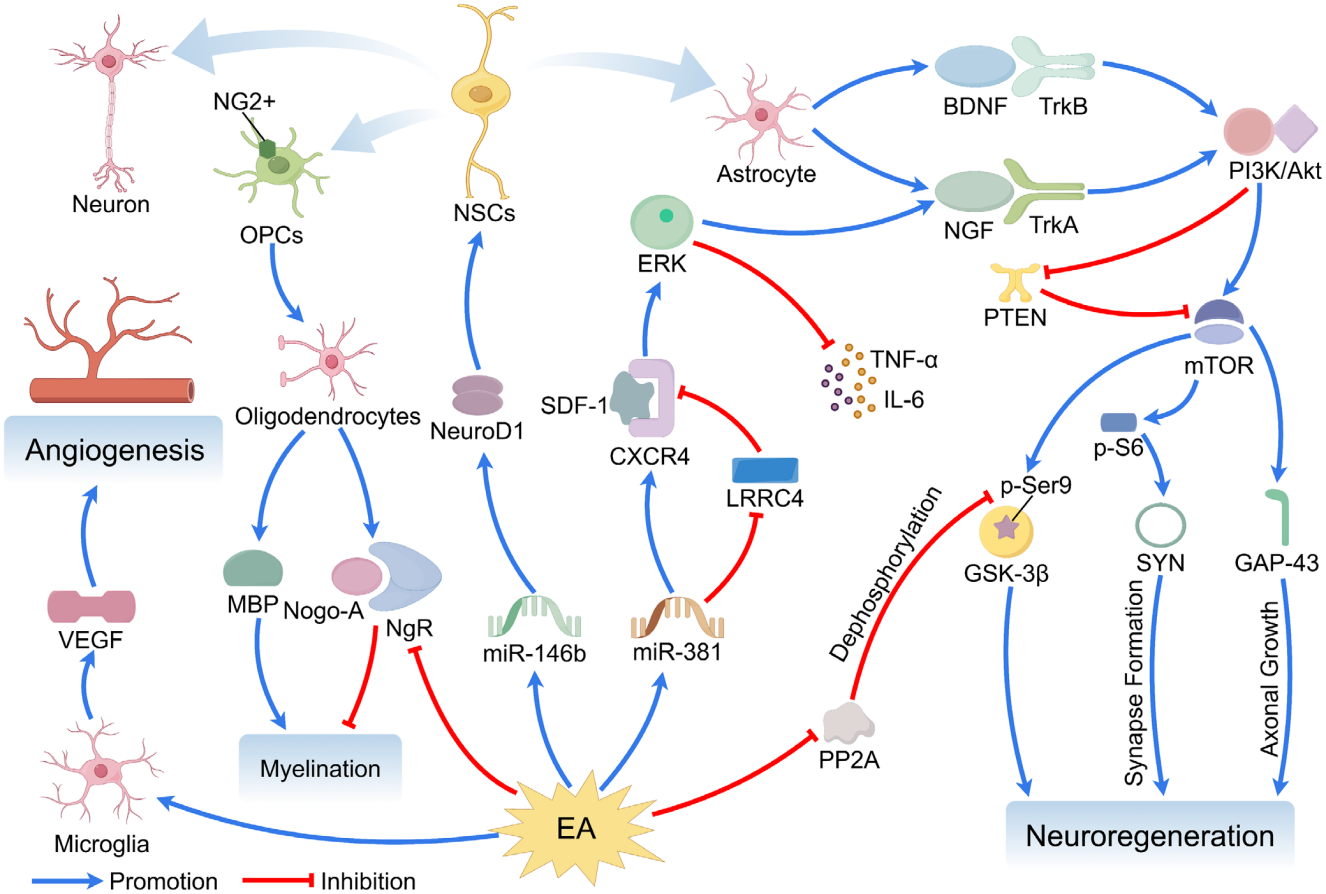
EA promotes neural regeneration and repair in IS. EA promotes the differentiation of neural stem cells into neurons and astrocytes, enhances the secretion of BDNF and NGF, and facilitates neural repair.

A critical aspect of recovery following IS involves intrinsic neural regeneration and structural and functional repair processes, collectively termed neuroplasticity. These processes include neurogenesis, axonal sprouting, synaptogenesis, myelination, and the reorganization of neural circuits. EA has been extensively investigated for its capacity to promote neural regeneration and repair in the ischemic brain (Figure 11).

One key mechanism by which EA facilitates recovery is through modulating neural oscillations and correcting abnormal brain electrical activity [204]. This electrophysiological modulation is thought to create a more favorable environment for neuronal function and contribute directly to the neural rehabilitation process [204]. Furthermore, acupuncture and EA interventions are known to stimulate resident brain cells, such as astrocytes and microglia, to release crucial neurotrophic factors [187]. These factors, including BDNF and Nerve Growth Factor (NGF), play vital roles in promoting neuronal survival, growth, and differentiation, thereby facilitating neural network remodeling and functional recovery in damaged brain areas [187].

Evidence from systematic reviews and experimental studies suggests that acupuncture and EA can promote endogenous neurogenesis, the process by which new neurons are generated from neural stem cells (NSCs) [205]. This encompasses the proliferation, migration, and differentiation of NSCs in specific brain regions affected by ischemia [205]. Different acupuncture stimulation methods and the selection of specific acupoints may target neurogenesis in distinct brain regions. For instance, EA at GV20 and GV14 notably elevates mRNA levels of BDNF and VEGF [122]. Both BDNF and VEGF signaling pathways are crucial for promoting the proliferation and differentiation of NSCs, particularly in the hippocampus and subventricular zone (SVZ) of the ipsilateral hemisphere following IS, ultimately aiding functional recovery [122]. EA also enhances serum and brain NGF levels while reducing serum Nogo‐A in IS rats, facilitating neural repair in ischemic brain tissue [123]. Nogo‐A is a protein known to inhibit axonal growth, so its reduction may help promote neural circuit rewiring. Acupuncture at the DU26 point has been suggested to boost self‐healing mechanisms by enhancing CBF (as discussed in Section 2.3.1), promoting neurogenesis, and potentially regulating gene transcription or the expression of glycogen synthase kinase‐3 (GSK‐3) and protein phosphatase 2A (PP2A) [124].

Beyond neurogenesis, EA also supports other aspects of neural repair, including myelin regeneration. EA enhances myelin basic protein (MBP) expression in white matter and suppresses the expression of the axonal growth inhibitors Nogo‐A and its receptor (NgR), thereby facilitating myelin regeneration around damaged axons [125, 126]. This is crucial for restoring efficient nerve signal transmission.

Molecular mechanisms involving exosomes and microRNAs also play a role in EA‐induced neurogenesis and repair. EA increases the expression of exosomal markers tumor susceptibility gene 101 (TSG101) and CD81 in the peri‐ischemic striatum. By upregulating exosomal microRNA‐146b (miR‐146b), EA enhances NeuroD1‐mediated differentiation of neural stem cells into neurons, providing a novel mechanism by which EA promotes neurogenesis and improves neurological deficits [127]. Furthermore, EA at GV20 and GV14 may facilitate neurological repair after IS by modulating the miR‐381/leucine‐rich repeat‐containing 4 (LRRC4) axis. EA upregulates miR‐381 expression, which selectively inhibits LRRC4 and subsequently activates the SDF‐1/C‐X‐C chemokine receptor 4 (CXCR4) signaling pathway [128]. The SDF‐1/CXCR4 axis is known to be involved in cell migration and repair processes, thus promoting recovery from neurological injury [128].

EA also influences the survival and differentiation of specific precursor cell populations. EA stimulation has been shown to enhance the survival of NG2‐expressing cells, a type of glial progenitor, in perilesional tissue by reducing the expression of inflammatory factors like TNF‐α (as discussed in Section 2.1.1) [130]. Furthermore, EA promotes the survival and differentiation of NG2‐expressing cells by activating BDNF and its associated signaling pathways, including tropomyosin receptor kinase B (TrkB) and GSK‐3β [130]. This process drives the differentiation of NG2‐expressing cells into various cell types, including oligodendrocytes (for myelination), endothelial cells (for angiogenesis), and even microglia/macrophages (for immune modulation) [130], highlighting their multipotency and EA's broad influence on brain cell populations.

Neurorepair is a highly complex and multilevel regulated process, in which neuroplasticity [206] is regarded as the core foundation of functional recovery [207]. Neuroplasticity not only encompasses axonal regeneration and remodeling but also critically depends on synaptogenesis [208], which refers to the formation, maturation, and stabilization of new synapses, that is, the establishment and functional integration of synaptic connections both between neurons and between neurons and their target cells. This process is highly dynamic and tightly regulated, occurring not only during early neural development but also throughout adulthood [209], and is robustly reactivated following neural injury [210].

Accumulating evidence indicates that synaptogenesis is a fundamental biological event in neurorepair and is indispensable for effective neural regeneration [207]. After neural injury, axonal regeneration alone is insufficient to restore neurological function; newly formed axons must establish stable and functional synaptic connections with appropriate target neurons to enable the reconstruction of neural circuits [211, 212]. Therefore, synaptogenesis is considered the final and decisive step by which neural regeneration transitions from “structural repair” to “functional recovery.” In the absence of proper synapse formation and stabilization, regenerated axons may remain functionally silent or form aberrant connections. This, in turn, limits the efficacy of neurorepair.

At the molecular level, synaptogenesis is a multistage and finely regulated process that includes synapse initiation, synaptic differentiation, and synaptic maturation [213]. This process is orchestrated by a variety of signaling molecules, including cell adhesion molecules [214] (such as neurexins and neuroligins), synaptic scaffold proteins (e.g., PSD‐95 [215, 216] and synaptophysin [SYP]), neurotrophic factors [217], and activity‐dependent signaling pathways. Following nervous system injury, the expression and function of these synapse‐related proteins are often disrupted, leading to synapse loss and impaired synaptic plasticity. Consequently, restoring synaptogenesis is critical for rebuilding functional neural networks and effective information transmission.

Notably, adeno‐associated virus (AAV)‐shROCK2–mediated inhibition of ROCK2 enhances monoaminergic axonal sprouting and promotes neurite outgrowth, synaptogenesis, and neurogenesis, thereby improving poststroke functional recovery [197]. Moreover, accumulating evidence suggests that EA can activate the BDNF/TRKB/CREB signaling pathway, upregulate the expression of synaptic plasticity–related proteins, and improve synaptic ultrastructure, thereby facilitating synaptogenesis and synaptic functional recovery and ultimately alleviating poststroke cognitive impairment in rats subjected to cerebral ischemia–reperfusion injury [198].

During neuroplasticity, axonal remodeling and synaptic alterations are interdependent and occur in a coordinated manner. EA stimulation at ST36 and PC6 has been shown to modulate the mechanistic target of rapamycin (mTOR) signaling pathway, which is critical for protein synthesis and cell growth. This modulation enhances the expression of neuroplasticity‐associated proteins, such as growth‐associated protein 43 (GAP‐43), involved in axonal growth, and SYP, a marker of synapses [131]. Additionally, EA increases the phosphorylation levels of key signaling molecules within this pathway, including AKT, mTOR, ribosomal protein S6 (S6), and phosphatase and tensin homolog (PTEN) [131]. These molecular changes contribute to the promotion of corticospinal tract (CST) axonal sprouting at the C1–C4 levels of the spinal cord [131], which in turn facilitates neuroprotection and enhances neural plasticity, supporting the restoration of motor and sensory functions [131]. In addition to the mTOR signaling pathway, regulation of actin filament dynamics represents a critical mechanism underlying synaptogenesis and structural plasticity. Neurite extension, presynaptic terminal formation, and stabilization of postsynaptic structures all depend on dynamic remodeling of the actin cytoskeleton. EA stimulation at LI11 and ST36 improves neurological outcomes and reduces infarct size, potentially associated with the upregulation of LIM domain kinase 1 (LIMK1) protein expression and the downregulation of Slingshot homolog 1 (SSH1) protein expression. These changes likely contribute to the inhibition of cofilin‐mediated actin filament dynamics [132], thereby promoting synapse formation, maintaining synaptic structural stability, and enhancing the efficacy of neurorepair [132]. Moreover, EA can modulate synaptic plasticity through epigenetic regulation. Evidence indicates that EA downregulates the expression of miR‐134, thereby relieving its negative regulation of LIMK1 and subsequently upregulating and activating LIMK1 signaling. This mechanism rescues synaptic–dendritic plasticity in the hippocampal CA1 region after IS, ultimately leading to improvements in learning and memory functions [218]. EA coordinately regulates axonal remodeling and synaptogenesis through multiple signaling pathways. It not only promotes neural repair at the structural level but also facilitates neural network reorganization by reconstructing functional synaptic connections, thereby improving neurological outcomes following IS.

Finally, EA promotes functional network reorganization, which underlies behavioral recovery. A study employing resting‐state functional magnetic resonance imaging (fMRI) has demonstrated that EA stimulation at ST36 and LI11 enhances cortico‐striatal network connectivity in individuals with IS. EA was observed to strengthen functional connections between the left striatum and various brain regions, suggesting an improvement in neural communication and integration within this critical motor circuit [133]. EA was also shown to reduce the characteristic path length and enhance the overall efficiency of the cortico‐striatal network [133]. Furthermore, EA stimulation at GV20 and GV14 has been shown to restore blood perfusion (as seen in Section 2.2.1) and improve neuronal interactions between the contralateral primary motor cortex (M1) and primary sensory cortex (S1) [134]. During the EA‐mediated recovery process, neuronal activity in the contralateral S1 plays a crucial role, highlighting the importance of the S1‐M1 circuit and its connectivity in poststroke functional recovery [134].

## 3 Electroacupuncture in Improving Symptoms and Sequelae of Ischemic Stroke

### 3.1 Improvement of Learning, Memory, and Cognitive Function

**FIGURE 12:**
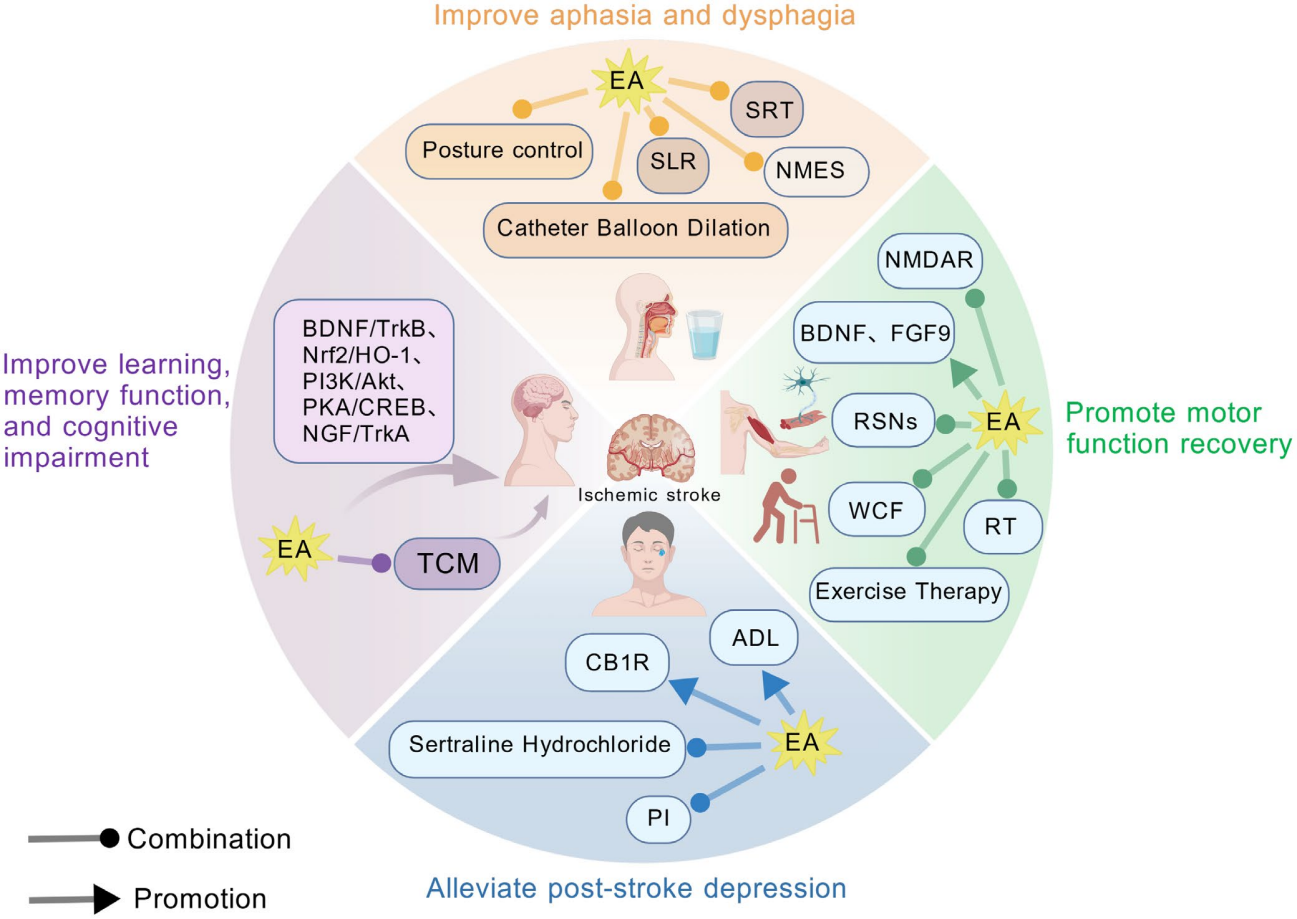
EA in improving symptoms and sequelae of IS. EA alleviates IS symptoms and sequelae, improving learning, memory, cognition, and motor function while promoting recovery from aphasia, dysphagia, and poststroke depression (created with BioGDP.com [12]).

Poststroke cognitive impairment (PSCI) and VaD are significant sequelae of IS, profoundly impacting patients' quality of life and increasing caregiver burden. These cognitive deficits often manifest as impairments in learning, memory, executive function, and attention. EA has demonstrated considerable potential in enhancing learning and memory functions and alleviating cognitive impairment following stroke (Figure 12, which also illustrates cognitive function recovery). EA's beneficial effects on cognition are multifaceted, involving the modulation of neuroplasticity, reduction of neuroinflammation, and protection of neuronal integrity through various pathways.

One key mechanism involves mitigating the detrimental effects of neuroinflammation and oxidative stress on cognitive circuits. EA improves learning and memory deficits in MCAO rats, partly by activating the Nrf2/HO‐1 signaling pathway, a major regulator of antioxidant responses. This activation facilitates the M2 polarization of microglia and macrophages, shifting these immune cells toward a pro‐resolving and anti‐inflammatory phenotype [135]. Furthermore, EA improves memory deficits by downregulating the expression of the purinergic receptor P2X7 (P2X7) and components of the inflammasome pathway like NLR Family Pyrin Domain Containing 3 (NLRP3), while concurrently upregulating Nrf2 and promoting M2 polarization of microglia [137]. These actions collectively reduce the neuroinflammatory milieu that impairs cognitive function. EA can also improve cognitive dysfunction induced by cerebral ischemia–reperfusion injury (CIRI) by inhibiting overall microglial polarization and neuroinflammation, a mechanism mediated through the phosphorylation of p38 mitogen‐activated protein kinase (MAPK) [138]. Specifically, EA stimulation at GV20 and GV24 has been shown to alleviate inflammation in the hippocampal CA1 region, a brain area critical for memory formation, through the inhibition of the NF‐κB signaling pathway. This reduction in inflammation in the hippocampus significantly contributes to improving memory and learning deficits after cerebral infarction [140].

EA's impact on neurotrophic factors and synaptic plasticity is crucial for cognitive recovery. EA stimulation at GV20 and Sishencong (EX‐HN 1) can activate the BDNF/TrkB signaling pathway, promoting the restoration of synaptic plasticity and improving learning and memory function in rats with CIRI [24]. Neurotrophic factors like BDNF and NGF are essential for neuronal survival, synapse formation, and function. EA may exert its therapeutic effects on learning and memory impairments through multiple signaling pathways known to regulate neuronal function and plasticity, including PI3K/Akt, PKA/CREB, NGF/TrkA, JAK2/STAT3, Notch, and Eph/hepatocyte growth factor signaling pathways [219]. While interactions between these pathways are complex, their collective modulation by EA suggests a broad impact on the molecular substrates underlying cognitive function [219].

Protecting mitochondrial integrity is another pathway contributing to EA's cognitive benefits. EA may alleviate PSCI by enhancing mitochondrial integrity in the hippocampus through increased expression of SIRT1, peroxisome proliferator‐activated receptor γ coactivator‐1α (PGC‐1α), and optic atrophy 1 (OPA1), key regulators of mitochondrial biogenesis and fusion, while downregulating the expression of dynamin‐related protein 1 (DRP1), involved in mitochondrial fission [136]. Maintaining healthy mitochondrial function is vital for the high energy demands of cognitive processes.

Furthermore, proteomic analyses provide insight into broader protein changes induced by EA. A proteomics analysis showed that EA stimulation at GV20 and GV24 can regulate dysregulated proteins associated with brain and neural development following CIRI, thereby improving cognitive deficits [139]. Specifically, EA was found to modulate components of the Pten/Akt signaling pathway by increasing the expression of p21‐activated kinase 4 (Pak4), protein kinase Bγ (Akt3), and ephrin‐B2 (Efnb2), highlighting potential mechanisms through which EA facilitates neural repair and cognitive recovery by influencing pathways involved in cell survival, growth, and synaptic signaling [139].

EA's influence on neurochemical systems also impacts cognition. EA stimulation at the GV20 and DU24 acupoints enhances the 5‐Hydroxytryptamine 1A (5‐HT1A) receptor‐mediated PKA signaling pathway and increases intracellular calcium levels regulated by NMDA receptor activation [142]. These neurochemical changes are known to play roles in synaptic transmission and plasticity crucial for learning and memory processes [142].

The complex relationship between autophagy and cognitive function is also influenced by EA. While the overall effect of EA on autophagy poststroke is debated, some studies link specific aspects of autophagy modulation to cognitive benefits. For example, EA at GV24 and GV20 has been shown to enhance autophagy and improve postischemic learning and memory deficits by activating the PI3K/Akt signaling pathway. In a MCAO rat model, EA treatment increased the mRNA and protein levels of Beclin‐1, mammalian target of rapamycin (mTOR), and phosphoinositide 3‐kinase (PI3K), promoted Akt phosphorylation, and suppressed p53 expression [141]. This suggests that specific patterns of autophagy modulation orchestrated by EA might contribute positively to cognitive outcomes.

Clinical evidence also supports the use of acupuncture‐based therapies for cognitive impairment. CT‐guided scalp ring acupuncture (SCN) combined with traditional Chinese herbal therapy has demonstrated promising therapeutic effects for postinfarct vascular dementia (PIVD), a common form of vascular cognitive impairment [143]. While this specific study involves a combined therapy and a different stroke subtype, it underscores the clinical relevance of cranial acupuncture approaches for vascular cognitive deficits.

In summary, EA improves learning, memory, and cognitive function after IS through a complex interplay of mechanisms, including the reduction of neuroinflammation and oxidative stress, enhancement of neurotrophic factor signaling and synaptic plasticity, preservation of mitochondrial integrity, modulation of key signaling pathways (e.g., Nrf2/HO‐1, BDNF/TrkB, PI3K/Akt, NF‐κB, mTOR, Pten/Akt), influence on neurochemical systems (e.g., 5‐HT1A, NMDA receptors), and potential modulation of autophagy. These multi‐target effects converge to protect neurons, promote neuronal repair, and facilitate the functional reorganization of cognitive circuits.

### 3.2 Improvement of Aphasia and Dysphagia

Aphasia and dysphagia are two of the most common and debilitating sequelae following IS, significantly impacting patients' communication abilities, nutritional status, and increasing the risk of complications like aspiration pneumonia. EA and traditional acupuncture approaches have shown promise in facilitating the recovery of language‐related neural pathways to improve poststroke aphasia and in enhancing the coordination and function of swallowing muscles to alleviate dysphagia (Figure 12, which also illustrates cognitive function recovery).

#### 3.2.1 Poststroke Aphasia

Aphasia, a disorder affecting the production or comprehension of language, results from damage to language centers in the brain. While the primary evidence base in the provided text focuses on dysphagia, there is some indication that acupuncture‐based therapies may support language recovery. Studies investigating the combined use of therapies show potential. Head acupuncture (HA), a specific application of acupuncture on the scalp, combined with Shur language rehabilitation (SLR) has been shown to significantly improve language abilities and enhance treatment efficacy in patients with poststroke aphasia [220]. This suggests that combining acupuncture approaches with targeted language therapy may offer synergistic benefits for language recovery, although the specific mechanisms of EA/acupuncture on language processing circuits warrant further dedicated investigation.

#### 3.2.2 Poststroke Dysphagia

Dysphagia, or difficulty swallowing, is a critical concern due to the risks of malnutrition, dehydration, and aspiration pneumonia. Acupuncture and EA have been extensively studied as interventions for poststroke dysphagia. A review highlights the simplicity, safety, and efficacy of acupuncture therapy for dysphagia. It has been shown to modify cortical neuron excitability, promote the recovery of swallowing and neurological functions, and improve dysphagia across different stages and types, without causing significant adverse effects [221]. This suggests broad applicability and safety.

Specific acupoint selections and techniques are employed for dysphagia. In an experiment by Qin et al. [22], acupuncture at Lianquan (CV23) and TE17, along with additional points, was found to be more effective than conventional swallowing rehabilitation training in enhancing local glossopharyngeal function following a stroke. This highlights the potential of specific acupoint prescriptions. A study comparing different ES parameters found that low‐frequency EA at Fengfu (GV16) and CV23 yields superior results in treating poststroke dysphagia compared to high‐frequency EA [222], suggesting that optimal stimulation parameters are important for efficacy. Ultrasound‐guided EA targeting the suprahyoid muscle group has emerged as a refined technique. It has been shown to be safer and more effective than both traditional MA and conventional EA, specifically by enhancing the movement of the hyoid‐laryngeal complex, a key action in swallowing, thus improving swallowing function in patients with poststroke pharyngeal dysphagia [223]. This indicates the potential benefit of precise targeting and potentially higher stimulation intensity safely delivered to swallowing muscles.

Acupuncture and EA are frequently integrated with conventional rehabilitation methods to enhance outcomes for dysphagia. EA combined with swallowing training has proven effective in improving cerebral microcirculation, enhancing nerve conduction velocity, and strengthening swallowing muscle function, ultimately facilitating the restoration of swallowing abilities after a stroke [144, 145]. Cervical and dorsal plexus acupuncture, when used alongside conventional rehabilitation training, may improve both swallowing and respiratory function in poststroke dysphagia patients [146]. This is likely achieved through enhanced cerebrovascular hemodynamics and the regulation of neurotrophic factors, both of which play critical roles in functional recovery [146]. The combination of EA with posture control and traditional rehabilitation techniques has been shown to be more beneficial than conventional rehabilitation alone. This integrated approach not only improves swallowing function but also significantly reduces the incidence of serious complications such as aspiration, aspiration pneumonia, and nutritional disorders, thus enhancing the patient's overall quality of life [147]. Combined acupuncture and catheter balloon dilation have demonstrated synergistic effects in treating cricopharyngeal achalasia (CPA), a specific swallowing difficulty after brainstem infarction [148]. This approach significantly improves swallowing function and reduces aspiration in complex cases [148]. Furthermore, a randomized controlled trial indicated that scalp and cervical acupuncture, when combined with neuromuscular electrical stimulation (NMES) and rehabilitation training, significantly improves pharyngeal swallowing difficulties in patients recovering from a stroke. This multimodal combination enhances treatment efficacy and improves both swallowing function and quality of life [149].

In summary, EA and various acupuncture techniques are valuable adjuncts for managing poststroke aphasia, particularly when combined with language therapy. Their role is even more strongly supported in the treatment of poststroke dysphagia, where they demonstrate efficacy in improving swallowing function, muscle coordination, and reducing complications. The mechanisms likely involve modulating cortical excitability, improving blood flow and neurotrophic support to swallowing‐related brain areas and nerves, enhancing swallowing muscle function, and potentially regulating local reflex arcs. The evidence suggests significant benefits, particularly when acupuncture/EA is integrated into comprehensive rehabilitation programs.

### 3.3 Promotion of Motor Function Recovery

Motor deficits, including weakness, paralysis, impaired coordination, and spasticity, are among the most prevalent and disabling sequelae of IS, severely limiting patients' functional independence and quality of life. EA is a widely used intervention aimed at promoting the recovery of motor functions by stimulating neuroplasticity, facilitating the reorganization of motor networks, improving synaptic transmission, and alleviating muscle spasticity (Figure 12). EA and acupuncture treatments have demonstrated significant efficacy in improving various aspects of motor function. Studies show improvements in forelimb muscle strength, sensorimotor function, and coordination in animal models following ischemia/reperfusion injury [129, 152, 153]. Notably, the therapeutic effects of acupuncture and EA at acupoints such as GV20 and ST36 were comparable to those of edaravone, a widely used stroke medication in clinical practice, in promoting motor recovery, vascular regeneration, and neural repair in rats [152]. This suggests EA/acupuncture could serve as an effective alternative or complementary therapy to conventional treatments [152]. Movement trajectory analysis has provided more granular insight, showing that EA stimulation at ST‐36 and Yinlingquan (SP‐9) can normalize the movement patterns of ischemic gerbils [153], indicating recovery of coordinated motor control. EA treatment has also been shown to alleviate depressive symptoms, enhance overall neurological function, and improve the ability to perform activities of daily living (ADLs), reflecting broad benefits that include motor improvements [150, 151].

The promotion of neuroplasticity and repair within motor pathways is a core mechanism. Therapeutic ES, which encompasses approaches like transcranial cortical stimulation [224] and peripheral somatosensory stimulation, has been shown to exert synergistic effects in improving motor function after stroke [225]. The underlying mechanisms involve modulating inflammatory cytokines through the regulation of STAT1 and NF‐κB activation and promoting the release of neurotrophic factors, such as BDNF and fibroblast growth factor 9 (FGF9) [225]. These molecular changes enhance neuronal survival and facilitate recovery, contributing to the restoration of motor abilities [225]. EA stimulation directly modulates neuronal activity within motor circuits, for example, by reducing the activity of pyramidal neurons and enhancing the activity of parvalbumin (PV) interneurons. This regulation of neuronal excitability and inhibition is achieved through the modulation of NMDAR expression, ultimately improving sensory‐motor function [154]. Neuroplasticity also involves structural changes at the cellular and synaptic levels. EA treatment reduces cofilin rod formation and microtubule‐associated protein 2 (MAP2) degradation in the cortical penumbra, indicating improved neuronal structural integrity [157]. Cofilin and MAP2 are critical components of the cytoskeleton, essential for neuronal morphology and connectivity. Additionally, EA inhibits the mitochondrial translocation of cofilin and the activation of caspase‐3, thereby suppressing apoptosis [157]. Research also indicates that EA and rehabilitation training (RT) enhance motor function recovery by increasing the expression of neuroplasticity markers like GAP‐43 and SYP in regions like the hippocampal CA3 [159], reflecting enhanced axonal sprouting and synaptogenesis. The modulation of actin filament dynamics, crucial for neurite outgrowth and synaptic remodeling, through regulation of proteins like LIM domain kinase 1 (LIMK1) and Slingshot homolog 1 (SSH1) and their effect on cofilin also contributes to EA's neuroprotective and recovery‐promoting effects on motor function [132].

Functional reorganization of brain networks is a critical aspect of motor recovery. A study using resting‐state functional MRI (fMRI) combined with independent component analysis (ICA) demonstrated that EA treatment can enhance motor recovery by modulating the functional architecture of resting‐state networks (RSNs) [156]. Specifically, EA was found to promote the reorganization of key neural networks, including the sensorimotor network (SMN), interoceptive network (IN), default mode network (DMN), and salience network (SN). This remodeling of neural connectivity is thought to improve the efficiency of information processing and integration within these networks, thereby enhancing motor function and overall neurological recovery [156].

Spasticity, characterized by increased muscle tone and exaggerated reflexes, is a common and disabling motor sequela. EA and acupuncture are frequently used to alleviate spasticity. EA preconditioning may alleviate poststroke spasticity by inhibiting the NF‐κB/NLRP3 signaling pathway, thereby attenuating IS‐induced inflammation and apoptosis [161]. Furthermore, EA may modulate the gut‐brain axis by elevating the levels of specific metabolites like propionyl acetate and butyl acetate in the intestine [161], suggesting a potential systemic link for spasticity modulation. Clinical evidence supports these findings, showing that combining EA with conventional routine care can reduce spasticity within 180 days poststroke, leading to improvements in overall and lower limb motor function as well as daily living activities in patients with spasticity [226].

Combining EA/acupuncture with conventional rehabilitation therapies often yields superior results for motor recovery. Integrating EA with constraint‐induced movement therapy (CIMT) improves functional recovery in rats after IS [158]. Similarly, EA and rehabilitation training (RT) enhance motor function recovery in rats with CIRI [159]. In stroke patients, the integration of EA with cycling training has been shown to significantly enhance motor function, leading to substantial improvements in muscle contraction and strength [160]. This combined approach facilitates a more efficient recovery rate, promotes greater independence in daily activities, reduces disability, and improves the overall quality of life [160]. An external application of the wet compress formula (WCF) has also been shown to significantly enhance the therapeutic efficacy of EA in improving upper limb motor dysfunction (ULMD) induced by IS without causing severe side effects [155], highlighting the potential for synergistic effects with topical treatments.

In summary, EA promotes motor function recovery through a complex interplay of mechanisms that target the brain, spinal cord, and muscles. These include enhancing neuroplasticity and neural repair pathways (neurotrophic factors, synaptic proteins, axonal sprouting, cytoskeletal dynamics), modulating neuronal circuit activity and functional connectivity, and specifically addressing disabling sequelae like spasticity through anti‐inflammatory and potentially systemic effects. Combined therapy approaches, integrating EA with various rehabilitation techniques, show particular promise in maximizing motor recovery and improving functional outcomes.

### 3.4 Alleviate of Poststroke Depression

Poststroke depression (PSD) is a common and often debilitating neuropsychiatric sequela of IS, significantly impacting recovery, cognitive function, and quality of life. EA has shown considerable efficacy in alleviating PSD by modulating neurobiological pathways involved in emotional regulation, including the regulation of neurotransmitter levels and enhancement of endorphin release, thereby improving emotional health (Figure 12).

EA's antidepressant effects are mediated through several mechanisms. EA alleviates depressive‐like behavior in PSD by activating the Sonic hedgehog (Shh) signaling pathway, which contributes to reducing inflammation and oxidative stress [162]. These pathways are increasingly recognized for their roles in the pathophysiology of depression. EA also alleviates depressive‐like behavior and cognitive dysfunction in mice with PSD by activating CB1R [163], suggesting a role for the endocannabinoid system in mediating EA's effects on mood and cognition. Furthermore, EA improves neurological deficits and enhances spontaneous activity and exploratory behavior, commonly impaired in depression, in PSD rats [164]. It exerts neuroprotective effects by reducing iron deposition, inhibiting lipid peroxidation, and enhancing antioxidant defenses, thereby suppressing ferroptosis in neurons of the prefrontal cortex, a brain region critical for mood regulation, and consequently improving depressive‐like behavior [164].

EA is often used in combination with other therapies to manage PSD. The Tiaoshen Jieyu acupuncture method (“Regulating Shen and Relieving Depression”) is an established approach in traditional Chinese medicine for emotional dysregulation. When combined with conventional antidepressant medication like sertraline hydrochloride tablets, EA targeting specific acupoints has been shown to effectively reduce the severity of depression, enhance neurological function, improve activities of daily living (ADLs), and promote sleep quality in patients with PSD [28]. This combined treatment demonstrates superior clinical efficacy compared to sertraline hydrochloride alone and may mitigate the adverse side effects typically associated with the medication [28]. Similarly, a controlled trial indicates that combining EA with psychological intervention (PI) is a safe and effective approach for treating PSD, suggesting synergistic benefits when addressing both the biological and psychological aspects of depression [165].

## 4 Integration of EA With Other Approaches

While EA demonstrates significant standalone potential as a complementary therapy for managing IS and its sequelae, combining EA with other therapeutic modalities offers a powerful strategy to leverage synergistic effects and achieve more comprehensive patient benefits. This integration seeks to address the multifaceted pathophysiology of stroke more effectively than single interventions alone.

### 4.1 Combination With Pharmacological and Herbal Agents

Integrating EA with pharmacological or traditional herbal medicines represents a common combined approach. EA combined with oxygen therapy (OM) effectively regulates the balance between proapoptotic (Bax) and anti‐apoptotic (Bcl‐2) protein expression in the hippocampal CA1 region of rats with CIRI [166], potentially enhancing ischemic outcomes by reducing cell death. EA combined with conventional Western medicine, broadly defined, has been shown to improve neurological function and ADLs in patients with acute ischemic stroke (AIS) by regulating the imbalance between pro‐inflammatory (IL‐17) and anti‐inflammatory (IL‐10) cytokines [167]. The clinical efficacy of this combined treatment approach has been reported to surpass that of conventional Western medicine alone, suggesting that EA may enhance therapeutic outcomes in AIS patients through immunomodulation and facilitation of functional recovery [167].

Traditional herbal components combined with acupuncture/EA also show promise. Studies have shown that natural products exert neuroprotective effects in stroke by multitarget regulation of mitochondrial dysfunction, including mitochondrial biogenesis, dynamics, autophagy, and apoptosis [227]. Ginsenoside Rb3 directly targets indoleamine 2,3‐dioxygenase 1 (Ido1) and activates Ido1/Opa1‐mediated mitochondrial fusion, thereby promoting neural stem cell migration, proliferation, and neurogenesis after IS, ultimately improving long‐term neurological function [228]. In focal cerebral ischemia rats, the combination of acupuncture and Gastrodin significantly increases Nestin‐ and Neuron‐specific enolase‐immunoreactive (NSE‐IR) positive cell counts while decreasing GFAP‐positive cell counts in the CA1 and CA3 hippocampal regions [170]. This suggests enhanced neurogenesis and neuronal differentiation alongside reduced glial scarring, potentially leading to improved brain function [171]. Similarly, the combination of EA and Polysaccharide Gastrodin (PGB) significantly increases Nestin and Stem Cell Factor (SCF) expression in the dentate gyrus of ischemic rats [172], further supporting the promotion of neurogenesis by this combination. Another herbal compound, tenuigenin (TE), when combined with EA, enhances the survival, migration, and differentiation of human mesenchymal stem cells (hMSCs) into neural cell lineages [173]. This combination also inhibits astrocyte formation, promotes neurogenesis, and facilitates the recovery of both motor and cognitive functions [173].

### 4.2 Combination With Cellular and Biologics Therapies

The integration of EA with cellular therapies, particularly stem cell‐based approaches, is an area of growing interest. A comprehensive review highlights the multifaceted potential of stem cell therapy itself in mitigating the devastating consequences of stroke, emphasizing its immunomodulatory properties, trophic effects, and neuroprotective capabilities [229]. Combining stem cell transplantation with acupuncture has been proposed to improve the survival rate, homing efficiency, and functional differentiation of implanted stem cells, enhancing the effects of each therapy and addressing the limitations of individual treatments [182]. Indeed, integrating bone marrow stromal cell (BMSC) transplantation with EA treatment has been shown to markedly enhance neurological recovery in animal stroke models [174].

Beyond cell transplantation, combining EA with biologically active factors or extracellular vesicles shows promise. A randomized controlled trial indicated that the combination of SMES and NGF is a safe and effective treatment for promoting recovery from IS [168]. Investigating the mechanism, it was found that during the brain ischemia repair phase, SMES can enhance BBB permeability [223]. This transient increase in permeability may facilitate the entry of therapeutic agents like NGF into the brain [169], thereby enhancing its neurotrophic effects. The combination of EA and small extracellular vesicles derived from induced pluripotent stem cells (iPSC‐EVs) has demonstrated neuroprotective effects by modulating immune responses and inhibiting glial activation [175]. This combined approach suppressed pro‐inflammatory Th1 and Th17 responses while enhancing anti‐inflammatory Treg activity. Additionally, it regulated the interleukin‐33 (IL‐33)/suppression of tumorigenicity 2 (ST2) signaling axis, leading to reduced activation of microglia and astrocytes [175]. These effects contributed to improved neurological function, a decrease in infarct volume, and reduced neuronal apoptosis in MCAO mice [175], suggesting that EA combined with iPSC‐EVs may enhance poststroke recovery through coordinated immunomodulation and neuroprotection.

In summary, the integration of EA with various pharmacological agents, herbal compounds, and cellular/biologics therapies offers a powerful avenue to enhance poststroke recovery. These combinations leverage the complementary mechanisms of each modality, such as EA's neuroprotective, anti‐inflammatory, and pro‐regenerative effects, alongside the specific actions of the combined agent (e.g., drug effects, stem cell trophic support, exosome cargo delivery). This synergistic approach holds significant potential for developing more effective and comprehensive stroke treatments.

## 5 Neurobiological Basis and Methodological Challenges of Electroacupuncture in Ischemic Stroke

To advance mechanistic understanding and clinical translation of EA in IS, it is essential to integrate evidence from neurobiological research with methodological analysis. Particularly in the context of multicenter randomized controlled trial (RCT) design, mechanistic plausibility and reproducibility of intervention parameters are equally critical. Accordingly, this section first reviews recent anatomical and neuroimaging evidence supporting potential structure–function relationships underlying acupoint‐based stimulation (Section 5.1). Building on these mechanistic insights, we then address a major translational challenge—namely, the substantial heterogeneity of EA intervention parameters—and discuss stage‐oriented standardization strategies informed by existing preclinical and clinical evidence (Section 5.2).

### 5.1 Anatomical and Neuroimaging Evidence Supporting Meridian–Neuroanatomical Associations

Although the traditional concepts of acupoints and meridians originate from an experience‐based medical system, contemporary research has increasingly sought to explore their possible biological underpinnings and structure–function correlates from anatomical and neuroimaging perspectives. Advances in high‐resolution imaging technologies have provided new tools for investigating these associations.

At the local anatomical level, accumulating evidence suggests that certain acupoint regions may correspond to specific tissue characteristics. For example, ultrasound imaging studies have demonstrated that acupoint sites often exhibit lower electrical impedance, with imaging features correlated with the density of hyperechoic collagen bands, suggesting that interactions between acupuncture needles and local connective tissue may influence electrical properties [230, 231]. In addition, it has been proposed that meridian networks may have physical continuity with interstitial connective tissue networks [232, 233, 234]. Such structures may provide a substrate for acupoint‐specific neuromodulatory effects; however, their generalizability, specificity, and precise physiological significance remain to be further clarified.

At the systemic and central levels, advanced neuroimaging techniques have provided objective evidence for how acupuncture modulates brain function. Functional magnetic resonance imaging (fMRI) studies indicate that stimulation of specific acupoints can modulate brain activity and connectivity in poststroke patients. Specifically, acupuncture has been shown to selectively activate ipsilesional brain regions [176], regulate functional connectivity within networks related to motor and language functions [235, 236, 237], and influence limbic–paralimbic–neocortical networks, which may underlie its multimodal effects, including analgesia [238]. Resting‐state fMRI analyses further demonstrate that acupuncture can alter the network integration of regions such as the parahippocampal gyrus [239] and reshape interactions among multiple resting‐state networks, including the default mode network and sensorimotor network, thereby facilitating reorganization of aberrant brain networks after stroke [195]. Moreover, diffusion tensor imaging studies suggest that acupuncture may be associated with improvements in the structural integrity of major white matter tracts, such as the corticospinal tract and superior longitudinal fasciculus, providing a potential structural basis for its role in promoting neurological recovery [235].

Taken together, contemporary anatomical and multimodal neuroimaging studies provide increasingly robust scientific clues for understanding the pathways through which acupuncture, including EA, exerts its effects. Investigations of local tissue properties suggest possible structural substrates of acupoints, while central neuroimaging evidence directly demonstrates the broad modulation of poststroke brain activity, network connectivity, and even white matter structure by peripheral stimulation. These findings help bridge traditional empirical concepts with modern neuroscience and offer partial explanations for the central mechanisms of acupuncture. Nevertheless, current studies remain limited by substantial heterogeneity, and most reported associations do not yet establish causality. Future research should therefore adopt more rigorous designs (e.g., sham‐controlled paradigms), integrate imaging techniques with high spatial and temporal resolution, and employ longitudinal approaches spanning different stages of stroke. Such efforts are needed to more precisely delineate the causal chain from “acupoint stimulation → neural pathways → brain functional remodeling → clinical recovery,” thereby providing a stronger evidence base for optimizing clinical acupoint selection and therapeutic strategies.

### 5.2 Heterogeneity of Electroacupuncture Parameters and Stage‐Oriented Standardization

In IS research, a major challenge to the reproducibility of EA effects and their clinical translation lies in the substantial heterogeneity of intervention parameters, including stimulation frequency, current intensity, session duration, treatment frequency, and acupoint selection. As summarized in Table 1, EA protocols vary considerably across both preclinical and clinical studies. Nevertheless, certain convergent patterns can be identified. These patterns provide a preliminary empirical basis for exploring relatively optimized parameter combinations tailored to different pathological stages of stroke (e.g., acute vs. recovery phases). Below, we examine key parameters, highlighting current heterogeneity and potential directions for stage‐specific standardization.

#### 5.2.1 Stimulation Frequency

Based on the preclinical and clinical studies summarized in Table 1, stimulation frequency represents one of the most heterogeneous EA parameters, ranging from low frequencies (e.g., 2 Hz) to high frequencies (e.g., 100 Hz), as well as disperse‐dense alternating waveforms (e.g., 2/15 Hz, 2/100 Hz). Notably, studies targeting different therapeutic objectives tend to adopt different frequencies. Low‐frequency or alternating‐frequency stimulation (e.g., 2/15 Hz) is most commonly used in acute and early subacute IS models and is often associated with outcomes related to neuroprotection, inflammatory modulation, and infarct volume reduction. In contrast, higher frequencies (e.g., 2/100 Hz) are more frequently applied in studies focusing on poststroke pain or functional symptoms during the recovery phase. These differences suggest that frequency selection should be closely aligned with the pathological stage of stroke and the primary therapeutic goal, with low‐frequency paradigms more suited to acute neuroprotection and high‐frequency paradigms potentially targeting symptom management during recovery.

#### 5.2.2 Current Intensity

Although current intensity varies across studies, the most commonly adopted adjustment criteria are the induction of mild, visible local muscle contraction in animal models or a strong but tolerable sensory response in clinical settings. As shown in Table 1, most studies employ low‐to‐moderate intensities (typically in the range of 0.5–3 mA), suggesting the existence of an empirically convergent safety–efficacy window, despite inconsistencies in reporting standards. This convergent range provides a practical foundation for defining baseline intensity references across studies and potentially across disease stages. However, future work should more precisely characterize stage‐specific intensity requirements, for example, distinguishing between periods of impaired consciousness and phases of active rehabilitation.

#### 5.2.3 Session Duration and Treatment Course

Single‐session duration varies from brief stimulation (5–10 min) to as long as 40 min, while treatment courses range from single interventions to daily repeated treatments lasting 1–2 weeks or longer. Despite this variability, synthesis of preclinical and clinical IS studies in Table 1 indicates that once‐daily EA sessions of approximately 20–30 min represent the most commonly employed regimen. This pattern may be regarded as an empirical “core protocol.” However, optimization of treatment frequency and total course length across different stages—such as the hyperacute versus chronic phase—remains an important area for future research to support stage‐specific recommendations.

#### 5.2.4 Acupoint Selection

Acupoint selection constitutes another major source of heterogeneity. Nevertheless, several acupoints or acupoint combinations—notably GV20, GV26, PC6, ST36, and LI11—are repeatedly used across independent studies and across different stroke stages. This recurrent use suggests experiential convergence despite the absence of formal standardization. Identification of these core acupoints provides a critical foundation for constructing an evidence‐informed acupoint repertoire that allows flexible, stage‐oriented combinations—for example, emphasizing arousal and consciousness recovery in the acute phase and limb function restoration in the recovery phase.

In summary, EA parameters used in IS exhibit a pattern of “heterogeneity with convergence.” Across stimulation frequency, current intensity, session duration, and acupoint selection, empirically derived convergent patterns have emerged, and these patterns appear to be associated with different pathological stages of stroke and corresponding therapeutic objectives. An important future direction is therefore to systematically explore and validate differentiated, stage‐specific parameter schemes—covering acute, subacute, recovery, and sequelae phases—while acknowledging the need for flexibility. Such efforts will help advance EA toward more reproducible and precision‐oriented clinical application.

Based on the synthesis of shared parameter features identified across existing studies (Table 1), we further propose a phase‐specific reference framework of core EA parameters (Table S1) to provide methodological support for the design of future standardized research. It should be emphasized that this framework is not derived from head‐to‐head, evidence‐based guideline comparisons, nor is it a prescriptive “optimal” regimen. Rather, it represents an empirical synthesis and consensus portrayal of parameters most frequently used in the existing literature. Its aims are twofold: (1) to provide a pragmatic starting reference that may reduce heterogeneity and improve cross‐study comparability in future experimental designs; and (2) to propose a set of testable hypotheses—namely, whether particular parameter combinations are more advantageous at specific stages of stroke—thereby encouraging rigorous factorial designs to optimize and validate these parameters.

## 6 From Preclinical Evidence to Clinical Application: A Translational Perspective and Biomarker Exploration

Although this review systematically summarizes the complex molecular mechanisms of EA in IS, most of the evidence is derived from rodent models. A central question remains: can these findings be translated to, and validated in, human stroke patients—and how? Direct confirmation of specific molecular pathways in the human brain (e.g., Nrf2 nuclear translocation or NLRP3 inflammasome activation in brain tissue) is limited by technical and ethical constraints. Therefore, bridging preclinical research and clinical application must rely on surrogate, human‐observable biomarkers.

At present, several indirect but compelling lines of evidence support the translational relevance of animal mechanisms to humans. First, peripheral blood biomarkers provide the most accessible molecular evidence. Clinical studies have shown that EA can modulate the imbalance between pro‐inflammatory (e.g., IL‐1β, TNF‐α) and anti‐inflammatory (e.g., IL‐10) cytokines in patients with acute IS [167], which is highly consistent with the anti‐inflammatory effects observed in animal models [32, 33, 34]. Similarly, changes in serum neurotrophic factors (e.g., BDNF) after EA directly echo its pro‐plasticity actions in animal studies [24, 122]. Second, multimodal neuroimaging offers macroscopic evidence for EA effects in the human brain. Functional MRI studies demonstrate that EA modulates functional connectivity within key resting‐state networks, such as the sensorimotor network and the default mode network, in poststroke patients [133, 194, 195]. These network‐level plasticity changes are considered systems‐level correlates of synaptic remodeling and neurogenesis observed in animal models. Diffusion tensor imaging further suggests that EA is associated with improved structural integrity of white matter tracts, such as the corticospinal tract [235], providing clinical structural support for mechanisms involving axonal growth and remyelination reported in preclinical studies [125, 126].

Based on this evidence, we propose that future clinical translation should focus on developing and validating a multimodal “EA‐response biomarker panel,” rather than relying on a single indicator. This panel could include: (1) Neuroimaging biomarkers, such as functional connectivity strength within specific networks (e.g., the sensorimotor network), as dynamic indicators of neuroplastic reorganization; (2) Peripheral molecular biomarkers, capturing an “anti‐inflammatory signature” (e.g., decreased IL‐1β with increased IL‐10) and a “neurotrophic signature” (e.g., BDNF levels), reflecting EA‐induced modulation of systemic and central microenvironments; and (3) Emerging liquid‐biopsy biomarkers, such as specific microRNAs carried by circulating exosomes (e.g., miR‐146b [127], miR‐210 [118]), which may serve as fingerprints of particular reparative processes, including neurogenesis and angiogenesis.

Establishing clear associations between these biomarkers and clinical outcomes (e.g., motor recovery, cognitive improvement) is a critical step toward precision EA therapy. Future studies should adopt prospective clinical trial designs and integrate multi‐omics analyses with artificial intelligence approaches [240] to synthesize multidimensional biomarker data. This strategy will not only help validate the relevance of preclinical mechanisms in human biology, but also enable patient stratification, personalized EA treatment planning, and real‐time monitoring of therapeutic response—thereby advancing EA from experience‐based practice to evidence‐based, precision translational medicine.

## 7 Prospects

Looking forward, the field is moving toward optimizing EA therapy through personalized and precision medicine approaches. Future research should focus on identifying potential biomarkers that can predict individual responses to EA therapy. Based on each patient's specific clinical condition, genetic characteristics, underlying stroke pathology, and physiological responses, personalized EA treatment plans should be tailored to maximize efficacy and reduce the variability in treatment outcomes. Multi‐omics analysis offers a promising strategy for uncovering these individual differences and identifying predictive biomarkers. Metabolomics, for example, can identify dynamic metabolic changes associated with stroke and its recovery, providing insights for early diagnosis and evaluating treatment efficacy [241]. The combination of multi‐omics approaches, such as genomics, transcriptomics, proteomics, and metabolomics [240], can provide a comprehensive view of the biological state and response to therapy. In recent years, single‐cell sequencing has emerged as a powerful tool to dissect the role of specific cell populations, such as microglia, at a high resolution, revealing their key functions in immune responses and potentially their heterogeneous responses to EA [242]. Integrating vast datasets from multi‐omics and clinical sources presents a significant challenge, which is where advanced computational tools like machine learning (ML) and artificial intelligence (AI) come into play. Future studies could employ ML and AI to systematically analyze the complex data generated by omics network models, exploring the dynamic regulatory mechanisms of different acupuncture parameters on various biological pathways in a personalized manner. This would help to clarify the dose‐effect relationship and potential molecular mechanisms of acupuncture in treating IS, and critically identify personalized acupuncture targets, thus advancing the development of precision acupuncture strategies tailored to individual patient needs.

Compared to conventional pharmacological and surgical treatments, acupuncture generally provides a convenient, cost‐effective, and safe adjunctive therapy for IS. As research progresses, addressing the challenges in standardization and leveraging cutting‐edge technologies for personalized approaches will be key to fully realizing EA's potential in stroke care [243].

## 8 Conclusion

This review has systematically explored the multifaceted mechanisms underlying the beneficial effects of EA. These mechanisms can be broadly categorized into two major aspects: reducing ischemic damage and promoting neural repair and functional recovery. EA attenuates damage by modulating key pathological processes including the reduction of neuroinflammation and oxidative stress, the inhibition of excitotoxicity, and the suppression of various forms of regulated cell death such as apoptosis and ferroptosis. Furthermore, EA actively promotes recovery by enhancing beneficial processes including the regulation of autophagy, the preservation of BBB integrity, the regulation of CBF, and crucially, the promotion of angiogenesis and diverse aspects of neural regeneration and repair, including neurogenesis, neuroplasticity, and functional network reorganization.

Clinically, these complex biological effects translate into tangible improvements in stroke‐associated symptoms and sequelae. EA has demonstrated efficacy in alleviating cognitive impairment, improving aphasia and dysphagia, promoting motor function recovery, and alleviating poststroke depression. These benefits are often further enhanced when EA is integrated with other therapeutic modalities, such as pharmacological agents, herbal compounds, and cellular or biologics therapies, highlighting the potential for synergistic combination strategies.

In conclusion, IS remains a leading cause of global disability and mortality, necessitating the development of effective therapeutic strategies to mitigate injury and promote recovery. EA is a multi‐target therapeutic intervention that addresses the complex pathophysiology of IS from multiple angles. By simultaneously reducing injury and fostering the brain's intrinsic repair capabilities, EA offers a valuable addition to the therapeutic arsenal for improving outcomes and enhancing the quality of life for stroke survivors. Continued research and rigorous clinical validation will be essential to fully realize its potential in modern stroke care.

## Author Contributions

**Mingyue Zhao:** methodology, writing – original draft preparation. **Hangyu Shen:** methodology. **Xu Yan:** methodology. **Xiang Gao:** methodology. **Yuchun Liu:** methodology. **Shengjun Zhou:** methodology. **Jingxian Sun:** methodology. **Jie Sun:** conceptualization, writing – review and editing. **Yi Huang:** conceptualization, writing – review and editing, project administration. All authors have read and agreed to the published version of the manuscript.

## Funding

This study was supported by grants from National Natural Science Foundation of China (82405549), Ningbo Youth Science and Technology Innovation Leading Talent Project (2025QL015), Ningbo Top Medical and Health Research Program (2022020304), Zhejiang Province Traditional Chinese Medicine Project (2024ZL908), Ningbo Clinical Research Center for Emergency and Critical Diseases (2024L003).

## Ethics Statement

The authors have nothing to report.

## Consent

The authors have nothing to report.

## Conflicts of Interest

The authors declare no conflicts of interest.

## Supporting information


**Table S1:** Descriptive summary of commonly used EA parameters stratified by stroke stage.

## Data Availability

Data sharing is not applicable to this article as no datasets were generated or analyzed during this study.
