# Supplementary material for: Electroacupuncture Modulates Multiple Pathways for Neuroprotection and Neurorepair in Ischemic Stroke
Source: CNS Neurosci Ther. 2026 Apr 7;32(4):e70862. doi: 10.1002/cns.70862 (PMC13055200; doi:10.1002/cns.70862)
Supplement: Supplementary file 1 — Table S1: Descriptive summary of commonly used EA parameters stratified by stroke stage. [file CNS-32-e70862-s001.docx]

Supplementary Table 1. Descriptive summary of commonly used EA parameters stratified by stroke stage.

| Stroke stage | Primary therapeutic goals | Core EA parameter set | Rationale |
| --- | --- | --- | --- |
| Acute / Early Subacute Phase  (Animal models and early clinical stage) | Neuroprotection; suppression of inflammatory responses; reduction of cerebral edema; improvement of cerebral perfusion. | Stimulation frequency: Disperse–dense alternating waveform, predominantly 2/15 Hz.  Current intensity: Adjusted to induce mild, visible local muscle contraction in animal models; in clinical settings, set at a strong but tolerable sensory stimulation level.  Session duration: 20–30 minutes per session.  Treatment frequency and course: Typically one session per day for 7–14 days.  Core acupoints: Baihui (GV20), Shuigou (GV26), and Neiguan (PC6). | Disperse–dense low‑frequency stimulation (e.g., 2/15 Hz) represents one of the most commonly employed paradigms in preclinical ischemic stroke research and is frequently associated with outcomes related to neuroprotection, inflammatory modulation, apoptosis, and autophagy. GV20 and GV26 are repeatedly used in studies focusing on cerebral perfusion and disorders of consciousness, while PC6 is commonly applied to modulate autonomic function and the heart–brain axis. |
| Recovery / Chronic Phase  (Functional rehabilitation stage) | Promotion of neuroplasticity and angiogenesis; facilitation of functional reorganization; alleviation of post‑stroke symptoms. | Stimulation frequency: Selected according to specific functional goals. Frequencies of 2/10 Hz or 2/20 Hz are commonly applied for motor or swallowing rehabilitation, whereas 2/100 Hz is more frequently used for post‑stroke pain (e.g., hemiplegic shoulder pain).  Current intensity: Adjusted to elicit a comfortable deqi sensation or rhythmic contraction of the target muscle groups.  Session duration: Approximately 30 minutes per session.  Treatment frequency and course: Once daily or once every other day, 3–5 sessions per week, for 4–8 weeks.  Core acupoints: Based on the primary acupoints used during the acute phase, supplemented with meridian‑based point selection, such as Jianyu (LI15), Quchi (LI11), Zusanli (ST36), and Sanyinjiao (SP6). | Different stimulation frequencies may preferentially target distinct rehabilitation‑related mechanisms. At this stage, most studies combine body acupuncture, scalp acupuncture (e.g., MS6), and functional training, which may exert synergistic effects. Parameter selection should therefore be individualized according to the specific type of functional impairment, including motor, swallowing, cognitive, or affective deficits. |

Note: This table represents a descriptive synthesis and qualitative consensus derived from convergent parameter patterns identified in the preclinical and clinical studies summarized in Table 1. It reflects the most frequently used parameter combinations at each stroke stage in the literature, rather than results from head to head efficacy comparisons.
